# Cytotoxic Triterpenes from *Salacia crassifolia* and Metabolite Profiling of Celastraceae Species

**DOI:** 10.3390/molecules23061494

**Published:** 2018-06-20

**Authors:** Laila S. Espindola, Renata G. Dusi, Daniel P. Demarque, Raimundo Braz-Filho, Pengcheng Yan, Heidi R. Bokesch, Kirk R. Gustafson, John A. Beutler

**Affiliations:** 1 Laboratório de Farmacognosia, Universidade de Brasília, Campus Universitário Darcy Ribeiro, Brasília 70910-900, Brazil; renatadusi@hotmail.com (R.G.D.); dpdemarque@gmail.com (D.P.D.); 2 Molecular Targets Program, National Cancer Institute, Frederick, MD 21702, USA; yanpc@wzmc.edu.cn (P.Y.); bokeschh@mail.nih.gov (H.R.B.); gustafki@mail.nih.gov (K.R.G.); beutlerj@mail.nih.gov (J.A.B.); 3 FAPERJ/Departamento de Química, Universidade Federal Rural do Rio de Janeiro, Seropédica, RJ and Laboratório de Ciências Químicas, Universidade Estadual do Norte Fluminense, Campos dos Goytacazes, Rio de Janeiro 28013-602, Brazil; braz@uenf.br; 4 School of Pharmaceutical Sciences, Wenzhou Medical University, Wenzhou 325035, China; 5 Basic Science Program, Leidos Biomedical Research, Inc., Frederick National Laboratory for Cancer Research Sponsored by the National Cancer Institute, Frederick, MD 21702, USA

**Keywords:** cytotoxic activity, NCI-60 cancer cell line, pristimerin, *Salacia crassifolia*, Celastraceae, Brazilian Cerrado biome, *Salacia elliptica*, *Cheiloclinium cognatum*, *Plenckia populnea*

## Abstract

The new pentacyclic triterpene 11*β*-hydroxypristimerin (**1**), along with the known metabolites pristimerin (**2**), 6-oxopristimerol (**3**) and vitideasin (**4**), were isolated from a *Salacia crassifolia* root wood extract, following a bioassay-guided fractionation approach. Both the extract and the purified triterpenes displayed pronounced cytotoxic activity against human cancer cell lines. The NCI-60 cell line screen revealed that compound **2** was the most active, with a mean GI_50_ of 0.17 μM, while compound **1** had a mean GI_50_ of 8.7 μM. A COMPARE analysis of the screening results showed that pristimerin is likely to be the main compound responsible for the cytotoxic activity of the extract (mean GI_50_ of 0.3 μg·mL^−1^). A targeted search for pristimerin and related derivatives using LC-MS/MS revealed the presence of pristimerin (**2**) and 6-oxopristimerol (**3**) in all Celastraceae species examined and in all plant parts tested, while vitideasin (**4**) was only detected in the genus *Salacia*.

## 1. Introduction

Anticancer drugs derived from plants such as vinblastine, vincristine, etoposide, teniposide, paclitaxel (Taxol^®^), docetaxel, topotecan and irinotecan continue to have significant therapeutic value [1]; however, cancer remains a leading cause of death worldwide. In the research described below, we utilized bioassay-guided isolation of the cytotoxic constituents in selected plant extracts, the same approach that led to the discovery of some of these antitumor agents.

The Cerrado biome of Brazil has proven to be a rich source of cytotoxic plant extracts [2]. *Salacia crassifolia* (Mart. ex Schult.) G. Don, Celastraceae, popularly known as Bacupari-do-Cerrado, has been used by traditional communities to treat cancer [3]. It is a tree from the Cerrado biome adapted to the region’s dry climate with a well-developed root system. This species is cultivated for its edible fruit, used as an ornamental plant, and used for reforestation and restoration of degraded environments. Beyond its use against cancer, *S. crassifolia* is utilized in traditional medicine as an antimicrobial and anti-inflammatory agent [3]. Previous phytochemical investigations of the hexane extract of the leaves of *S. crassifolia* led to the isolation of friedelanes [4], commonly found in the leaves of Celastraceae species, and known as precursors of quinone methide triterpenoids found in the roots of these species [5,6]. Twenty-one extracts from *S. crassifolia*, *Salacia elliptica* (Mart.) G. Don, *Cheiloclinium cognatum* (Miers) A.C. Sm., and *Plenckia populnea* Reissek were analyzed by mass spectrometry to verify the distribution of pentacyclic triterpenoids in different plant parts of various Celastraceae species. In order to probe the cytotoxic potential and chemical profile of *S. crassifolia*, the hexane extract of its root wood was evaluated by high-throughput screening (HTS) against diverse tumor cells, together with the NCI-60 cell line screen [7]. Herein, we report the bioassay-guided isolation and structure elucidation of compounds **1**–**4** and their cytotoxic activity.

## 2. Results and Discussion

In our search for new cytotoxic agents, the hexane extract of *S. crassifolia* root wood was selected by preliminary high-throughput screening of Cerrado plant extracts, tested at 10 µg·mL^−1^ in eight cancer cell lines: Colo205 and KM12 (colon cancer), A498 and U031 (renal cancer), HEP3B and SKHEP (liver cancer), and MG63 and MG63.3 (osteosarcoma). While cytotoxic activity was observed in most cell lines, the extract did not inhibit the growth of either of the hepatic cancer cell lines. Dose-response experiments were then conducted with the root wood extract (N192803) in the colon, renal, and osteosarcoma cell lines. Based on its ^1^H NMR profile and selective cytotoxic activity, this extract was subsequently submitted for testing in the NCI-60 tumor cell assay [7].

### 2.1. Dose-Response Results

The extract of *S. crassifolia* root wood was cytotoxic against the KM12 colon cancer cell line (IC_50_ 1.7 μg·mL^−1^), but had no activity against Colo205 colon cancer cells at 20 μg·mL^−1^. Likewise, it was active against the A498 renal cancer cell line (IC_50_ 1.6 μg·mL^−1^), but showed no inhibitory activity at 20 μg·mL^−1^ for U031 renal cancer cells. Divergent activity in two renal cancer cell lines and two colon cancer cell lines provided evidence of potential selectivity among other cancer cell types, which can be further assessed in the NCI-60 cell screen. We considered this a favorable sign to justify a detailed study of this extract. When tested against osteosarcoma MG63 (non-metastatic) and MG63.3 (metastatic) cells, the extract inhibited cell growth with an IC_50_ of 3.7 μg·mL^−1^ and 5.7 μg·mL^−1^, respectively. Since these two related cell lines differ in their metastatic potential, the similar potency indicated that the sample does not differentially target metastatic osteosarcoma cells.

### 2.2. Extract NCI-60 Results

**Figure 1:**
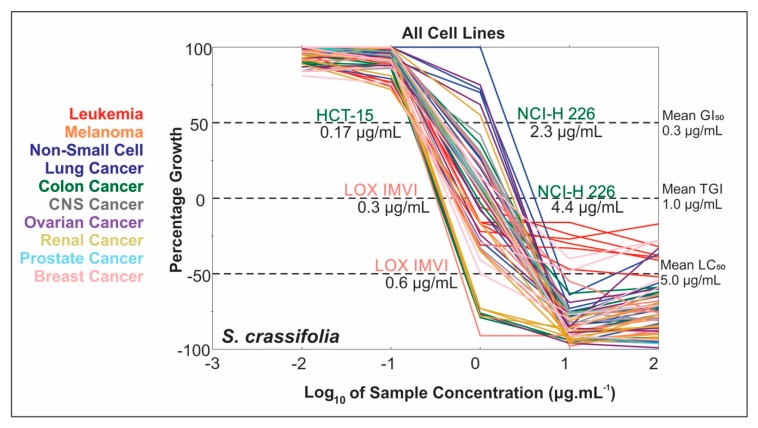
NCI-60 dose-response curves for the *Salacia crassifolia* root wood extract. Percentage growth of 100: cell growth is comparable to the control; percentage growth of 0: cells are still viable but no net cell growth relative to time zero; percentage growth of −100: complete cell killing, no cells are viable.

The hexane extract of *S. crassifolia* root wood (N192803) was submitted to both the NCI-60 cell line one-dose and five-dose screens. The mean GI_50_ (50% growth inhibition) was 0.3 μg·mL^−1^, a 3-fold lower concentration than its mean total growth inhibition (TGI) and 15-fold lower than the mean LC_50_ (Figure 1). The GI_50_ ranged from a low of 0.17 μg·mL^−1^ for the HCT-15 colon cancer cell line, to a high of 2.3 μg·mL^−1^, and growth inhibitory activity was observed in leukemia, melanoma, lung, colon, brain, ovarian, breast, prostate, and kidney cancer cell lines (Supplementary Materials).

### 2.3. Bioassay-Guided Fractionation of S. crassifolia

In order to identify the active compounds, the root wood hexane extract was initially fractionated on diol Solid Phase Extraction cartridges to give fractions of increasing polarity (A–E). Fraction B was eluted with CH_2_Cl_2_-EtOAc (20:1) and it showed an IC_50_ of 3.8 μg·mL^−1^ and 1.9 μg·mL^−1^ in renal (A498 and UO31), and 14.9 μg·mL^−1^ and 16.5 μg·mL^−1^ in osteosarcoma (MG63 and MG63.3) cancer cell lines. It was further fractionated on a Sephadex LH-20 column, giving 11*β*-hydroxypristimerin (**1**) in fraction B3 after final purification on HPLC; pristimerin (**2**) in fraction B5 and 6-oxopristimerol (**3**) in fraction B9. Another portion of fraction B3 after separation on a silica gel column gave vitideasin (**4**) in fraction B3-1, and compound **2** in B3-2. Fractions A and C from the initial diol fractionation also showed cytotoxicity in renal and osteosarcoma cell lines (Supplementary Materials). Investigation of these fractions showed that **2** is also a major component, thus explaining their growth inhibitory activity.

**Figure 2:**
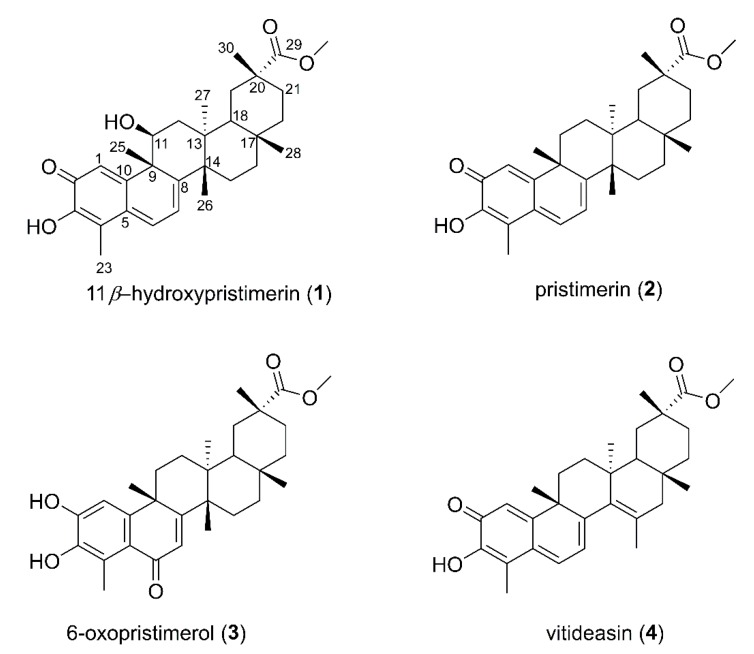
The structure of compounds isolated from *Salacia crassifolia*. 11*β*-hydroxypristimerin (**1**), pristimerin (**2**), 6-oxopristimerol (**3**) and vitideasin (**4**).

Compound **2** is the major secondary metabolite of the *S. crassifolia* extract, accounting for 69% of fraction B3, and it was present in fractions B2 to B7. Gonçalves et al. [8] reported the presence of **2** in a mixture containing another colorless compound that was obtained from the roots of *S. crassifolia*, and this mixture showed notably weaker antimicrobial activity than commercially available pristimerin. In this current study, the availability of high resolution chromatographic and spectroscopic techniques allowed the isolation and characterization of structurally similar compounds (**1**–**4**) (Figure 2) with differential cytotoxic potencies, as seen between **1** and **2**. Carvalho et al. [9] reported that Gonçalves et al. [10] isolated maytenin from *S. crassifolia*, but, in fact, Gonçalves described the isolation of maytenin from *Maytenus ilicifolia*.

### 2.4. Identification of Isolated Compounds from Salacia crassifolia

From a portion of the diol fraction B, 11*β*-hydroxypristimerin (**1**), a previously unreported compound, was isolated (see experimental for more details). The mass spectrum of **1** helped establish the molecular formula as C_30_H_38_O_4_ (*m*/*z* 463.2849; [M-H_2_O + H]^+^; error = 0.2 ppm). In the ^1^H NMR data (Supplementary Materials), we observed signals for three olefinic protons (H-1, H-6 and H-7) and, in the ^13^C spectrum, we observed eight olefinic carbons (C-1, C-3, C-4, C-5, C-6, C-7, C-8 and C-10) and a carbonyl carbon (C-2, δ 178.8). H-1, an α-carbonyl olefinic proton, was observed as a singlet (δ 7.32). H-6 and H-7 signals were observed as doublets at δ 6.88 and δ 6.28 (*J* = 7.2 Hz), respectively. Hence, compound **1** was characterized as a quinonemethide triterpene with a methyl ester group substituted in ring-E (carbonyl C-29 δ 178.7; methoxyl C-31 δ 51.8, H-31 δ 3.60)*.* The NMR data for **1** closely matched those reported for pristimerin **2** [11,12]. The only significant difference was an oxymethine assigned at C-11 (H-11, δ 4.57; dd, *J* = 12.2, 6.4 Hz) based on its coupling with H_2_-12. Molecular formula requirements established a hydroxy substituent at this position and the large vicinal coupling constants required an axial (*β*) orientation of H-11. Our spectroscopic analyses along with biogenetic considerations allowed assignment of the structure **1** as 11*β*-hydroxypristimerin.

**Figure 3:**
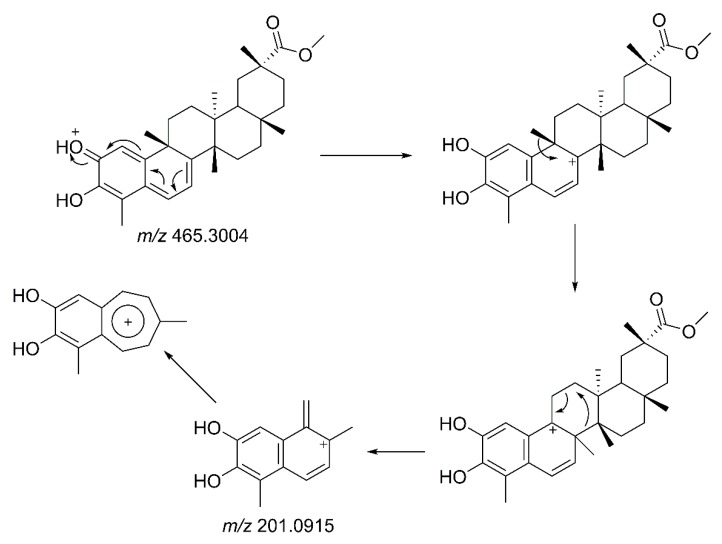
Proposed fragmentation for pristimerin (**2**).

Pristimerin (**2**) was identified in fraction B5, and 6-oxopristimerol (**3**) was detected in fraction B9. The ^1^H and ^13^C NMR data of these compounds were compared with literature values [11] to assure proper identification of the structures (NMR spectra and data are available in Supplementary Materials). LC-MS/MS analysis of pristimerin (**2**) indicated a molecular formula of C_30_H_40_O_4_ with the presence of a protonated ion ([M + H]^+^ *m*/*z* 465.3004; error: 0.1 ppm) and a sodiated adduct ([M + Na]^+^ *m*/*z* 487.2823; error: 0.4 ppm). When submitted to collisional activation, the protonated molecular ion generated a highly intense fragment at *m*/*z* 201.0915. The proposal for this fragmentation involves carbocation formation at C-8, followed by a methyl shift and a pericyclic fragmentation giving rise to *m*/*z* 201.0915 (Figure 3). This type of fragmentation pathway for pristimerin (**2**) was used to identify and confirm the presence of related triterpenoids in the extracts and fractions being studied.

LC-MS/MS analysis of 6-oxopristimerol (**3**) generated both the protonated molecular ion ([M + H]^+^ C_30_H_41_O_5_ *m*/*z* 481.2949; error: 1 ppm) and the sodiated adduct ([M + Na]^+^ C_30_H_40_NaO_5_ *m*/*z* 503.2771; error 0.5 ppm). The fragmentation of **3** follows the same pattern as pristimerin (**2**), although, due to the additional oxygen at C-6, the intense fragment ion is at *m*/*z* 217.0860 [12].

The mass spectrum of vitideasin (**4**) showed a protonated molecular ion ([M + H]^+^ C_30_H_39_O_4_ *m*/*z* 463.2878; error: 6.4 ppm) and its NMR spectroscopic data was compared with literature values [13] (NMR data and spectra are available in Supplementary Materials). Fragmentation of the protonated ion gave rise to a *m*/*z* 201.0899 ion, the same fragment observed with pristimerin (**2**).

In the LC-MS/MS analysis of the *S. crassifolia* root extract, it was possible to identify other triterpene quinonemethide derivatives, such as 6-oxopristimerol derivatives with an extra double bond ([M + H]^+^ C_30_H_39_O_5_ *m*/*z* 463.2847; error: 0.3 ppm) compared to 6-oxopristimerol (**3**). Fragmentation of the protonated molecule gave rise to an ion at *m*/*z* 217.0853, as seen with **3**. Due to the low yield of this metabolite, NMR analysis for structural assignment was not possible. Another compound identified by MS was a 11*β*-hydroxypristimerin derivative ([M + H]^+^ C_30_H_39_O_5_ *m*/*z* 479.2713; error: 17.6 ppm), which also had an extra unsaturation compared with 11*β*-hydroxypristimerin (**1**). Fragmentation of the protonated molecule gave rise to a *m*/*z* 201.0917 fragment. These results indicate that *S. crassifolia* is a rich source of additional quinonemethide triterpenoids and their derivatives.

### 2.5. Biological Activity of Isolated Compounds

**Figure 4:**
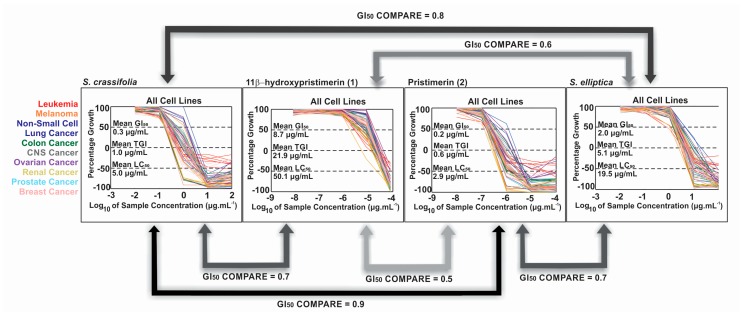
COMPARE correlations between **1**, **2** and *Salacia* extracts.

Compounds **1**, **2**, and **3** were submitted to the NCI-60 cell screen. In this National Cancer Institute screen, which utilizes 60 different human tumor cell lines, **2** exhibited a mean GI_50_ value of 0.23 μM, with individual values ranging from 0.12 μM (UO-31 renal and T-47D breast) to 1.2 μM (A549 non-small cell lung). Compound **1** was less potent, with a mean GI_50_ value of 8.7 μM, with the renal cancer cell line 786-O being the most sensitive. A COMPARE bioinformatic analysis resulted in a Pearson correlation factor of 0.9 between the crude *S. crassifolia* extract and compound **2**, indicating that it is likely the major compound responsible for the extract activity. A weaker correlation factor of 0.7 was found between the extract and **1** (Figure 4). Although compounds **1** and **2** are structurally similar, their mutual correlation factor was only 0.5, suggesting that a *β*-hydroxyl group at C-11 altered the mode of action and reduced the potency. In addition, oxidation of C-6 and loss of the quinone moiety in compound **3** drastically reduced its cytotoxic activity, making it ineligible to be tested in a full dose response in the NCI-60 screen. In accordance with the literature, triterpenes with a quinonemethide moiety in the A-ring display more potent cytotoxic activity in cancer cells [14].

### 2.6. LC-MS/MS Analysis of Extracts of Species from the Celastraceae Family

**Figure 5:**
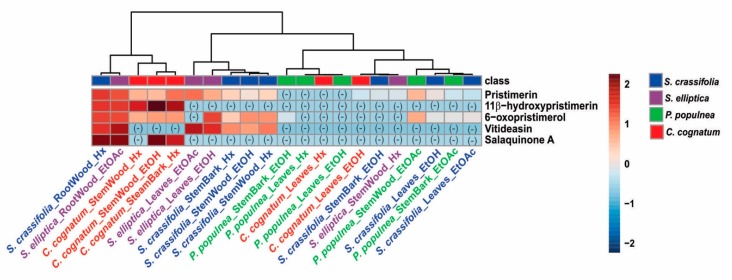
Heatmap and similarity analysis obtained using the MetaboAnalyst platform to analyze the metabolite profile of twenty-one different extracts from leaves, stem and root of *S. crassifolia*, *S. elliptica*, *C. cognatum* and *P. populnea*, obtained using hexane, ethyl acetate and ethanol as extraction solvents.

*S. crassifolia* root is a rich source of compound **2** and other pristimerin-like triterpenes. In order to identify the presence of quinonemethide triterpenes in other Celastraceae species, we performed targeted metabolite analysis of twenty-one different extracts from the leaves, stems, and roots of *S. crassifolia*, *S. elliptica*, *C. cognatum* and *P. populnea*, obtained using hexane, ethyl acetate or ethanol as extraction solvents. The compounds 11*β*-hydroxypristimerin (**1**), pristimerin (**2**), 6-oxopristimerol (**3**) and vitideasin (**4**) were monitored by LC-MS/MS, as well as the quinonemethide triterpenes isoiguesterin ([M + H]^+^ 403.3 > 201.3), a salaquinone A derivative ([M + H]^+^ 451.3 > 201.3), a tingenin B derivative ([M + H]^+^ 437.3 > 201.3), and tingenone ([M + H]^+^ 407.3 > 201.3). Of these latter compounds, only salaquinone A was detected in any of the extracts. In Figure 5, we summarize the findings of these analyses in a heatmap generated using the MetaboAnalyst platform [15].

Pristimerin (**2**) is a naturally occurring quinonemethide triterpenoid, restricted to plants of the Celastraceae [16]. Previously, it has shown cytotoxic [17], antimicrobial [4], anti-inflammatory [18], and antioxidant [9] activities. Earlier studies showed that pristimerin is found mainly in the roots of Celastraceae species, while its precursor friedelin, and other similarly cyclized intermediates, are found in the leaves [5,6]. A study of *S. campestris* and *Maytenus aquifolium* suggested a compartmentalized biosynthesis of quinonemethide triterpenoids in the root bark of Celastraceae species [6]. However, we detected pristimerin (**2**) and 6-oxopristimerol (**3**) in all plant parts of all species studied (Figure 5), including leaves, albeit at a lower concentration than in the roots. Detection of pristimerin (**2**) in the hydroalcoholic leaf extract of *Maytenus aquifolium* has previously been reported [19]. Salaquinone A and 11*β*-hydroxypristimerin were detected in *C. cognatum*, *S. crassifolia* and *S. elliptica* extracts, while vitideasin (**4**) was only detected in *Salacia* extracts (Figure 5).

The ethyl acetate extract of *S. elliptica* root wood showed a mean GI_50_ in the NCI-60 of 2.04 μg·mL^−1^, 6-fold greater than the GI_50_ of the *S. crassifolia* root wood hexane extract. The leukemia cell line MOLT-4 and breast cancer line T-47D were the most sensitive to this *S. elliptica* extract with a GI_50_ of 0.7 μg·mL^−1^. The *S. elliptica* extract had only 40% (LC-MS/MS peak area) of **2** relative to *S. crassifolia* and, indeed, it showed a weaker COMPARE correlation coefficient of 0.7 with **2** (Figure 4). The correlation coefficient between the two *Salacia* extracts was 0.8. The only two extracts that met the criteria for NCI-60 dose response testing were from the roots of *S. elliptica* and *S. crassifolia*, where **2** is more abundant, reinforcing that **2** is the predominant active compound in the extracts. Carneiro et al. [20] showed that the methanolic extract of *S. crassifolia* stem bark had no in vivo cytotoxic activity. In a similar finding from our study, the ethanolic extract of *S. crassifolia* stem bark was not active in any of the eight cancer cell lines in preliminary HTS. The lack of cytotoxic activity of these polar extracts is consistent with the low abundance of **2**, as seen in Figure 5. The *C. cognatum* stem bark hexane extract selectively inhibited 87% of MG63 cells at 10 μg·mL^−1^, and it had only 12.5% (LC-MS/MS peak area) of the pristimerin content, relative to the *S. crassifolia* extract (Figure 5).

In summary, the Brazilian Cerrado environment provides a rich source of bioactive plant metabolites. In particular, species of the Celastraceae offer numerous bioactive quinonemethide triterpenes, some of which have selective effects on cancer cell growth. In the case of *Salacia* species, the roots were rich in pristimerin (**2**). Compound **2** showed selective inhibitory activity towards a variety of human tumor cell lines and it was primarily responsible for the cytotoxic activity of the crude extracts. A co-occurring metabolite was identified as 11*β*-hydroxypristimerin (**1**), which is a new member of this triterpenoid class. These results demonstrate that the combined use of a targeted search for pristimerin and derivatives, cytotoxicity screening, and COMPARE analysis can efficiently identify the plant species and plant parts containing these and other growth inhibitory compounds.

## 3. Materials and Methods

### 3.1. General Experimental Procedures

UV absorption spectra were obtained using a Varian Cary 50 Bio UV−visible spectrophotometer (Agilent, Santa Clara, CA, USA). IR spectra were measured using a Perkin-Elmer FT-IR Spectrometer, SPECTRUM 2000 (Waltham, MA, USA). NMR spectra were acquired on a Bruker Avance III spectrometer (Billerica, MA, USA) operating at 600 MHz for ^1^H and 150 MHz for ^13^C and equipped with a 3 mm cryogenically cooled probe. ^1^H and ^13^C spectra were referenced to the residual deuterated solvent peaks. HRESIMS data were acquired on an Agilent 6530 Accurate Mass Q-TOF instrument (Santa Clara, CA, USA) and on a TripleTOF 5600 mass spectrometer (AB SCIEX, Framingham, MA, USA). Diol SPE fractionation of the extract was performed on DIO Spe-ed SPE cartridges (Applied Separations, Allentown, PA, USA), and subsequent fractions were separated on Sephadex LH-20 (GE Healthcare, Chicago, IL, USA). These fractions were then separated on silica gel. Sephadex LH-20 and silica gel columns were attached to a UA-6 UV detector and Foxy 200 fraction collector (Teledyne Isco, Lincoln, NE, USA). Final purifications were performed using a Varian ProStar HPLC with the indicated gradient and column. All solvents and chemicals used were of analytical grade.

### 3.2. Plant Extracts

*Salacia crassifolia* (Mart. ex Schult.) G. Don, Celastraceae, identified by esteemed Cerrado specialist botanist Prof. José Elias de Paula, was collected in August 2007, from the Cerrado *sensu stricto*-*campo aberto* of the Cerrado biome, Santo Antônio do Descoberto, Goiás State, at an altitude of 1169 m; 15°47′51.8″ S 48°17′44.3″ W. A voucher specimen was deposited in the Herbarium of the Universidade de Brasília under the accession number (UB) 3776. *Salacia elliptica* (Mart.) G. Don was collected in August 2007 at 1112 m; 15°49′00.5″ S 48°19′14.2″ W, Cerrado *campo sujo* ecosystem, voucher (UB) 3819. *Plenckia populnea* Reissek was collected in June 2007 at 1.243 m; 15°39′939″ S 47°53′195″ W, voucher (UB) 3747. *Cheiloclinium cognatum* (Miers) A.C. Sm. was collected in February 2009 at 961 m; 15°46′32.7″ S 47°38′59.5″ W, voucher (UB) 3805.

### 3.3. Antiproliferative Bioassay

The formazan endpoint assay developed by the Assay Development and Screening Section of the Molecular Targets Laboratory was used for the antiproliferative bioassays. The tests were carried out as described previously [21]. HTS was used to screen the hexane extract of *S. crassifolia* root wood at 10 μg·mL^−1^ in eight cancer cell lines: Colo205 and KM12 (colon cancer), A498 and U031 (renal cancer), HEP3B and SKHEP (liver cancer) and MG63 and MG63.3 (osteosarcoma). The extract was then further tested in dose response, from 20 μg·mL^−1^ to 1.3 μg·mL^−1^, in colon, renal and osteosarcoma cell lines, as well as in the NCI-60 cell assay. This screen utilizes 60 different human tumor cell lines, representing leukemia, melanoma and cancers of the lung, colon, brain, ovary, breast, prostate, and kidney. Three endpoints are calculated: (1) GI_50_, the concentration yielding a growth percent of 50 (i.e., 50% growth inhibition), (2) TGI, the concentration yielding a growth percent of 0, or Total Growth Inhibition, and (3) LC_50_, the concentration yielding a growth percent of −50, or lethality in 50% of the starting cells [7].

### 3.4. Bioassay-Guided Isolation of Compounds

The dried and powdered root wood (315.4 g) of *S. crassifolia* was subjected to repeated maceration with hexane, and the solvent was concentrated to dryness under reduced pressure at 30 °C yielding crude extract (3.2977 g). The extract was fractionated by solid phase extraction chromatography using a stepwise fractionation (2 g, 6 mL Diol cartridge, Applied Separations, Allentown, PA, USA). For the procedure, 864.3 mg of extract was dissolved in 12.8 mL of 1:1 CH_2_Cl_2_/MeOH and the soluble portion applied to 8 diol cartridges. After drying overnight, each cartridge was sequentially eluted with 6 mL of the following: 9:1 Hexane/CH_2_Cl_2_ (fraction A = 217.1 mg), 20:1 CH_2_Cl_2_/EtOAc (fraction B = 527.7 mg), EtOAc (fraction C = 82.7 mg), 5:1 EtOAc/MeOH (fraction D = 25.5 mg) then MeOH (fraction E = 9.9 mg). The fractions collected were dried under nitrogen gas, submitted for bioassay and analyzed by ^1^H NMR. Renal (UO-31 and A498) and osteosarcoma (MG63 and MG63.3) cancer cell lines were used for bioassay-guided isolation. Based on the biological results, a portion (373.65 mg) of fraction B was solubilized in 7.5 mL of hexane/CH_2_Cl_2_/MeOH (2:5:1) and fractionated on 3 Sephadex LH-20 columns using the same solvent mixture. For each column, 72 fractions were collected and combined in 10 fractions (B1 to B10) based on the UV chromatogram. The fractions collected were dried under nitrogen gas and analyzed by ^1^H NMR. A portion of the combined B3 fraction (71.4 mg) was subjected to silica gel column chromatography eluted with mixtures of CH_2_Cl_2_/EtOAc (92:8, 90:10, 85:15, 80:20, 70:30, 50:50, 40:60, 0:100) to yield 5 fractions: B3-1 (4.6 mg), B3-2 (49.29 mg), B3-3 (1.8 mg), B3-4 (8.05 mg) and B3-5 (7.1 mg). Another portion of the B3 fraction was purified using a Phenomenex Luna C8 (5 µ, 300 Å, 250 × 4.6 mm) column with 80% to 92% acetonitrile in water gradient (0–30 min), monitoring at 254 nm.

**11*β*-Hydroxypristimerin (1):** UV (MeOH) λ (log ε) 205 (4.34), 280 (3.54), 400 (3.30) nm; ^1^H NMR (CDCl_3_, 600 MHz) δ 7.32 (1H, s, H-1), 6.88 (1H, d, *J* = 7.2 Hz, H-6), 6.28 (1H, d, *J* = 7.2 Hz, H-7), 4.57 (1H, dd, *J* = 12.2, 6.4 Hz, H-11), 3.60 (3H, s, OC*H_3_*), 2.40, 1.74 (each 1H, m, H_2_-19), 2.38, 2.01 (each 1H, m, H_2_-12), 2.24, 1.40 (each 1H, m, H_2_-21), 2.19 (3H, s, H_3_-23), 2.00, 0.98 (each 1H, m, H_2_-22), 1.87, 1.51 (each 1H, m, H_2_-16), 1.64 (3H, s, H_3_-25), 1.62, 1.58 (each 1H, m, H_2_-15), 1.59 (1H, m, H-18), 1.30 (3H, s, H_3_-26), 1.20 (3H, s, H_3_-30), 1.10 (3H, s, H_3_-28), 0.61 (3H, s, H_3_-27), ^13^C NMR (CDCl_3_, 125 MHz) δ 178.8 (C, C-2), 178.7 (C, C-29), 167.2 (C, C-8), 161.6 (C, C-10), 145.9 (C, C-3), 132.2 (CH, C-6), 128.6 (C, C-5), 121.6 (CH, C-1), 118.8 (C, C-7), 118.0 (C, C-4), 65.3 (CH, C11), 51.8 (CH_3_, COOCH_3_), 48.1 (C, C-9), 44.9 (C, C-14), 43.9 (CH, C-18), 43.4 (CH_2_, C-12), 40.6 (C, C-13), 40.4 (C, C-20), 36.2 (CH_2_, C-16), 34.4 (CH_3_, C-25), 34.3 (CH_2_, C-22), 32.7 (CH_3_, C-30), 31.4 (CH_3_, C-28), 30.87 (C, C-17), 30.81 (CH_2_, C-19), 29.7 (CH_2_, C-21), 28.7 (CH_2_, C-15), 21.4 (CH_3_, C-26), 18.5 (CH_3_, C-27), 10.4 (CH_3_, C-23). HRESIMS *m*/*z* 463.2849 (calcd. for C_30_H_38_O_4_ + H^+^; [M − H_2_O + H]; error = 0.2 ppm).

### 3.5. LC-MS/MS Analysis of Isolated Compounds

For analysis of isolated compounds, an HPLC ekspert ultraLC 100 coupled with a TripleTOF 5600 mass spectrometer (AB SCIEX, Framingham, MA, USA) was used. For chromatographic separation, a Shim-pack XR-ODS C_18_ column (5 cm × 2.0 mm, 2.2 µm particle size) (Shimadzu, Kyoto, Japan) was used, coupled with a pre-column with the same material. The mobile phases were water and methanol (both with 0.1% of formic acid). The elution method initiated with 5% of methanol was kept isocratic for 1.0 min, which was then increased to 95% over 10 min. Four minutes were added to the method to wash and stabilize the column. The injection volume was 2 µL, and the column temperature was 40 °C. The positive ionization mode was employed and the ionization source parameters were CUR 22 (Curtain Gas), source temperature 450 °C, ionization voltage (IS) 5500 V, Gas1 (nebulization gas) 45, Gas2 (turbo heaters gas) 45 and DP 100 (declustering potential).

### 3.6. LC-MS/MS Analysis of Extracts of Species from the Celastraceae Family: Targeted Search for Pristimerin and Derivatives

Twenty-one different extracts from *P. populnea*, *C. cognatum*, *S. crassifolia* and *S. elliptica* were analyzed using an HPLC (Shimadzu LC-20AD, Kyoto, Japan) coupled with an ESI triple quadrupole mass spectrometer (ABSciex API 3200). For chromatographic separation, a Supelco Ascentis Express C_18_ column (15 cm × 4.6 mm, 2.7 µm particle size) (Bellefonte, PA, USA) was used. The mobile phases were water and methanol (both with 0.1% of formic acid). The elution method initiated with 70% of methanol, which was then increased to 100% for 5 min and kept isocratic until 11 min. Four minutes were added to stabilize the column. The injection volume was 10 µL, the column temperature was 45 °C and the autosampler temperature was 15 °C. The positive ionization mode was employed and the ionization source parameters were CUR 12 (Curtain Gas), source temperature 500 °C, ionization voltage (IS) 4500 V, CAD gas (collisionally-activated dissociation gas) 5, Gas1 (nebulization gas) 40 and Gas2 (turbo heaters gas) 40. The parameters DP (declustering potential) was 45, EP (entrance potential) 10, CE (collision energy) 25, CXP (collision cell exit potential) 3 and DT (Dwell time) 100. The transitions monitored were 465.3 > 201.2 (pristimerin), 481.3 > 201.2 (11*β*-hydroxypristimerin), 481.3 > 217.3 (6-oxopristimerol), 463.3 > 201.3 (vitideasin). Other quinonemethides triterpenes were also monitored, as 403.3 > 201.3 (isoiguesterin), 451.3 > 201.3 (salaquinone A derivative), 437.3 > 201.3 (tingenin B derivative) and 407.3 > 201.3 (tingenone). The area values were analysed in MetaboAnalyst 4.0 [15] (McGill University, Montreal, Canada) using log transformation and autoscaling for data scaling (mean-centered and divided by the standard deviation of each variable). The heatmap was built using Euclidean distance measure and Ward clustering algorithm. The data was grouped according to the species.

## Supplementary Materials

The following are available online: Detailed biological results, COMPARE correlations between the NCI-60 tested compounds and extracts; and the spectroscopic data of Compounds **1**, **2**, **3** and **4**.
