# Supplementary material for: Cytotoxic Triterpenes from Salacia crassifolia and Metabolite Profiling of Celastraceae Species
Source: Molecules. 2018 Jun 20;23(6):1494. doi: 10.3390/molecules23061494 (PMC6099938; doi:10.3390/molecules23061494)
Supplement: Supplementary file 1 [file molecules-23-01494-s001.pdf]

Supplemental Materials

Cytotoxic triterpenes from *Salacia crassifolia* and metabolite profiling of Celastraceae species

Laila S. Espindola [1,2,*], Renata G. Dusi [1,2], Daniel P. Demarque [1], Raimundo Braz-Filho [3], Pengcheng Yan [2,4], Heidi R. Bokesch [2,5], Kirk R. Gustafson [2] and John A. Beutler [2]

1 Laboratório de Farmacognosia, Universidade de Brasília, Campus Universitário Darcy Ribeiro, Brasília 70910-900, Brazil; renatadusi@hotmail.com (R.G.D.); dpdemarque@gmail.com (D.P.D.)
2 Molecular Targets Program, National Cancer Institute, Frederick, MD 21702, USA; yanpc@wzmc.edu.cn (P.Y.); bokeschh@mail.nih.gov (H.R.B.); gustafki@mail.nih.gov (K.R.G.); beutlerj@mail.nih.gov (J.A.B.)
3 FAPERJ/Departamento de Química, Universidade Federal Rural do Rio de Janeiro, Seropédica, RJ and Laboratório de Ciências Químicas, Universidade Estadual do Norte Fluminense, Campos dos Goytacazes, Rio de Janeiro, Brazil; braz@uenf.br
4 School of Pharmaceutical Sciences, Wenzhou Medical University, Wenzhou 325035, China
5 Basic Science Program, Leidos Biomedical Research, Inc., Frederick National Laboratory for Cancer Research sponsored by the National Cancer Institute, Frederick, MD 21702, USA
* Correspondence: darvenne@unb.br; Tel.: +55-61-3107-2016

List of Supporting Information

(S1) Cytotoxicity of *S. crassifolia* root wood hexane extract in colon COLO205 and KM12; renal A498 and U031; osteosarcoma MG63 and MG63.3 cancer cell lines.

(S2) NCI-60 single dose bar graph of *S. crassifolia* root wood extract.

(S3) NCI-60 single dose mean bar graph of *S. crassifolia* root wood extract.

(S4) NCI-60 dose response curves for *S. crassifolia* root wood extract.

(S5) NCI-60 5-dose mean bar graph of *S. crassifolia* root wood extract.

(S6) NCI-60 cumulative dose response curves for *S. crassifolia* root wood extract.

(S7) Cytotoxicity of DIOL fractions A-E of *S. crassifolia* root wood hexane extract in renal A498 and U031 cancer cell lines.

(S8) Cytotoxicity of DIOL fractions A-E of *S. crassifolia* root wood hexane extract in osteosarcoma MG63 and MG63.3 cell lines.

(S9) Cytotoxicity of DIOL fractions A-E of *S. elliptica* root wood ethyl acetate extract in renal A498 and U031 cancer cell lines.

(S10) Cytotoxicity of DIOL fractions A-E of *S. elliptica* root wood ethyl acetate extract in osteosarcoma MG63 and MG63.3 cell lines.

(S11) HRESIMS spectrum of 11*β*-hydroxypristimerin (**1**).

(S12) Infrared spectrum of 11*β*-hydroxypristimerin (**1**).

(S13) UV spectrum of 11*β*-hydroxypristimerin (**1**).

(S14) ^{1}H NMR spectrum (600 MHz) of 11*β*-hydroxypristimerin (**1**) in $CDCl_3$.

(S15) ^{13}C NMR spectrum (150 MHz) of 11*β*- hydroxypristimerin (**1**) in $CDCl_3$.

(S16) COSY spectrum of 11*β*-hydroxypristimerin (**1**) in $CDCl_3$.

(S17) HSQC spectrum of 11*β*-hyroxypristimerin (**1**) in $CDCl_3$.

(S18) HMBC spectrum of 11*β*-hydroxypristimerin (**1**) in $CDCl_3$.

(S19) NMR Spectroscopic Data (^{1}H 600 MHz, ^{13}C 150 MHz) for 11*β*-hydroxypristimerin (**1**) in $CDCl_3$.

(S20) Chromatogram, MS1 and MS2 spectra of the LC-MS/MS analysis of pristimerin (**2**).

(S21) ^{1}H NMR spectrum (600 MHz) of pristimerin (**2**) in $CDCl_3$.

(S22) ^{13}C NMR spectrum (150 MHz) of pristimerin (**2**) in $CDCl_3$.

(S23) COSY spectrum of pristimerin (**2**) in $CDCl_3$.

(S24) HSQC spectrum of pristimerin (**2**) in $CDCl_3$.

(S25) NMR spectroscopic data (^{1}H 600 MHz, ^{13}C 150 MHz) for pristimerin (**2**) in $CDCl_3$

(S26) Chromatogram, MS1 and MS2 spectra of the LC-MS/MS analysis of 6-oxopristimerol (**3**).

(S27) ^{1}H NMR spectrum (600 MHz) of 6-oxopristimerol (**3**) in $CDCl_3$.

(S28) ^{13}C NMR spectrum (150 MHz) of 6-oxopristimerol (**3**) in $CDCl_3$.

(S29) COSY spectrum of 6-oxopristimerol (**3**) in $CDCl_3$.

(S30) HSQC spectrum of 6-oxopristimerol (**3**) in $CDCl_3$.

(S31) HMBC spectrum of 6-oxopristimerol (**3**) in $CDCl_3$.

(S32) NMR spectroscopic data (^{1}H 600 MHz, ^{13}C 150 MHz) for 6-oxopristimerol (**3**) in $CDCl_3$

(S33) MS2 Mass spectrum of vitideasin (**4**).

(S34) ^{1}H NMR spectrum (600 MHz) of vitideasin (**4**) in $CDCl_3$.

(S35) ^{13}C NMR spectrum (150 MHz) of vitideasin (**4**) in $CDCl_3$.

(S36) COSY spectrum of vitideasin (**4**) in $CDCl_3$.

(S37) HSQC spectrum of vitideasin (**4**) in $CDCl_3$.

(S38) HMBC spectrum of vitideasin (**4**) in $CDCl_3$.

(S39) NMR spectroscopic data (^{1}H 600 MHz, ^{13}C 150 MHz) for vitideasin (**4**) in $CDCl_3$.

(S40) COMPARE between *S. crassifolia* and *S. elliptica* extracts.

(S41) COMPARE between *S. elliptica* extracts, pristimerin (**2**) and 11*β*-hydroxypristimerin (**1**).

(S42) NCI-60 single dose bar graph of *S. elliptica* root wood extract.

(S43) NCI-60 single dose mean bar graph of *S. elliptica* root wood extract.

(S44) NCI-60 dose response curves for *S. elliptica* root wood extract.

(S45) NCI-60 5-dose mean bar graph of *S. elliptica* root wood extract.

(S46) NCI-60 cumulative dose response curves for *S. elliptica* root wood extract.

(S47) NCI-60 dose response curves for 11*β*-hydroxypristimerin (**1**).

(S48) NCI-60 5-dose mean bar graph of 11*β*-hydroxypristimerin (**1**).

(S49) NCI-60 cumulative dose response curves for 11*β*-hydroxypristimerin (**1**).

(S50) NCI-60 dose response curves for pristimerin (**2**).

(S51) NCI-60 5-dose mean bar graph of pristimerin (**2**).

(S52) NCI-60 cumulative dose response curves for pristimerin (**2**).

(S53) NCI-60 single dose bar graph of 6-oxopristimerol (**3**).

(S54) NCI-60 single dose mean bar graph of 6-oxopristimerol (**3**).

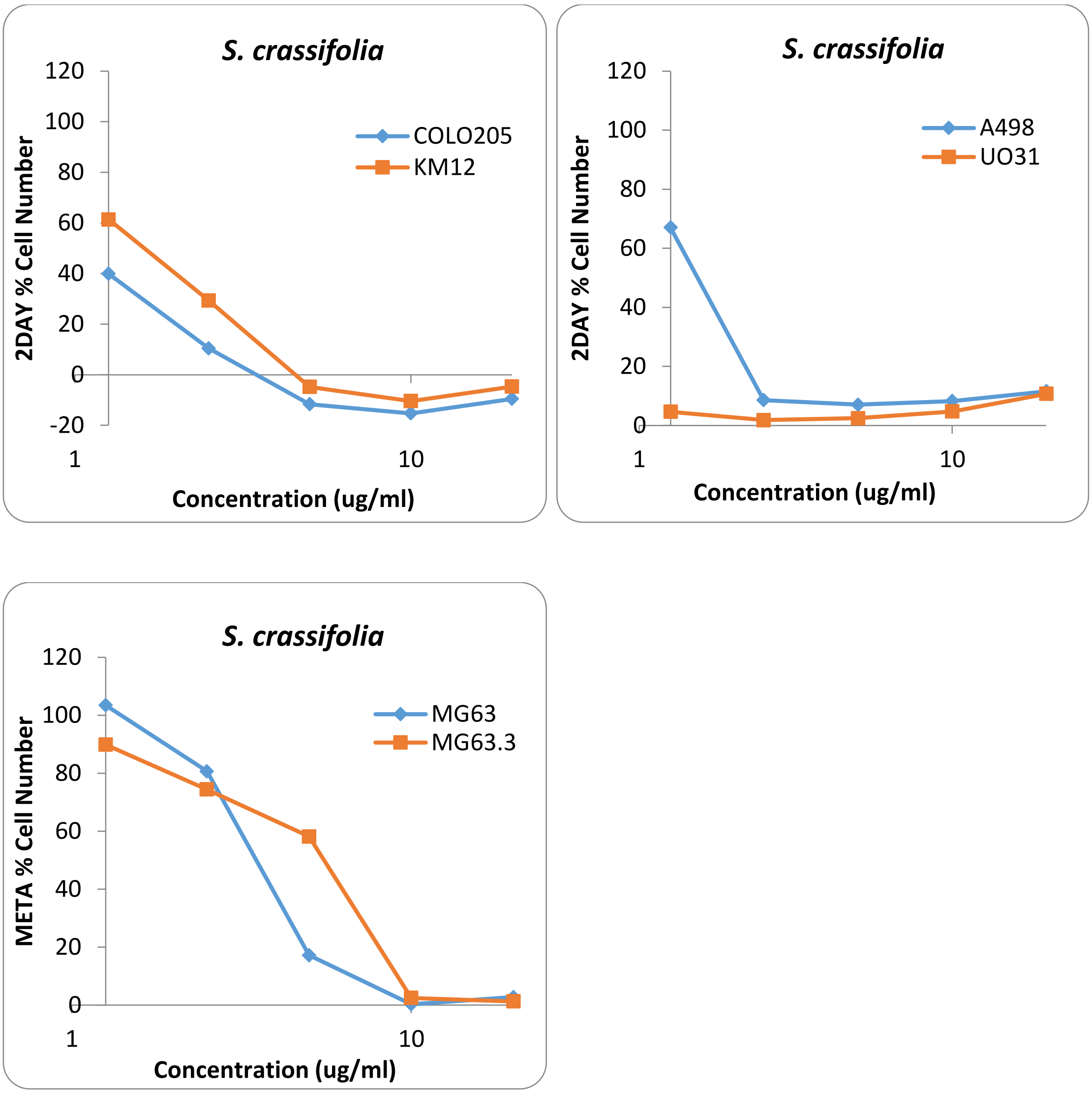

(S1) Cytotoxicity of *S. crassifolia* root wood hexane extract in colon COLO205 and KM12; renal A498 and U031; osteosarcoma MG63 and MG63.3 cancer cell lines.

Developmental Therapeutics Program

One Dose Bar Graph

NSC: D-N192803 / 1 | **Conc:** 1.00E2 ug/ml | **Test Date:** Mar 16, 2015

Experiment ID: 1503OS49 | **Report Date:** Jul 01, 2015

Panel/Cell Line	Growth Percent
Leukemia	
HL-60(TB)	-52.15
MOLT-4	-38.11
SR	-27.28
Non-Small Cell Lung Cancer	
A549/ATCC	-63.86
EKVX	-85.85
HOP-62	-79.95
HOP-92	-89.81
NCI-H226	-26.46
NCI-H23	-70.52
NCI-H322M	-69.77
NCI-H460	-54.96
NCI-H522	-66.50
Colon Cancer	
HCC-2998	-80.46
HCT-116	-73.21
HCT-15	-72.98
HT29	-62.99
KM12	-73.56
SW-620	-46.25
CNS Cancer	
SF-268	-63.45
SF-539	-77.61
SNB-19	-45.63
SNB-75	-76.78
U251	-70.72
Melanoma	
LOX IMVI	-72.14
MALME-3M	-63.74
M14	-51.75
MDA-MB-435	-66.00
SK-MEL-2	-73.79
SK-MEL-28	-45.37
SK-MEL-5	-66.39
UACC-62	-76.39
Ovarian Cancer	
OVCAR-3	-83.21
OVCAR-4	-69.22
OVCAR-5	-61.93
OVCAR-8	-74.14
NCI/ADR-RES	-61.94
SK-OV-3	-94.15
Renal Cancer	
786-0	-67.47
A498	-80.35
ACHN	-74.26
CAKI-1	-83.57
RXF 393	-51.57
SN12C	-33.64
TK-10	-84.64
UO-31	-77.35
Prostate Cancer	
PC-3	-83.42
DU-145	-83.72
Breast Cancer	
MCF7	-68.31
HS 578T	-30.17
BT-549	-82.02
T-47D	-34.20
MDA-MB-468	-39.23

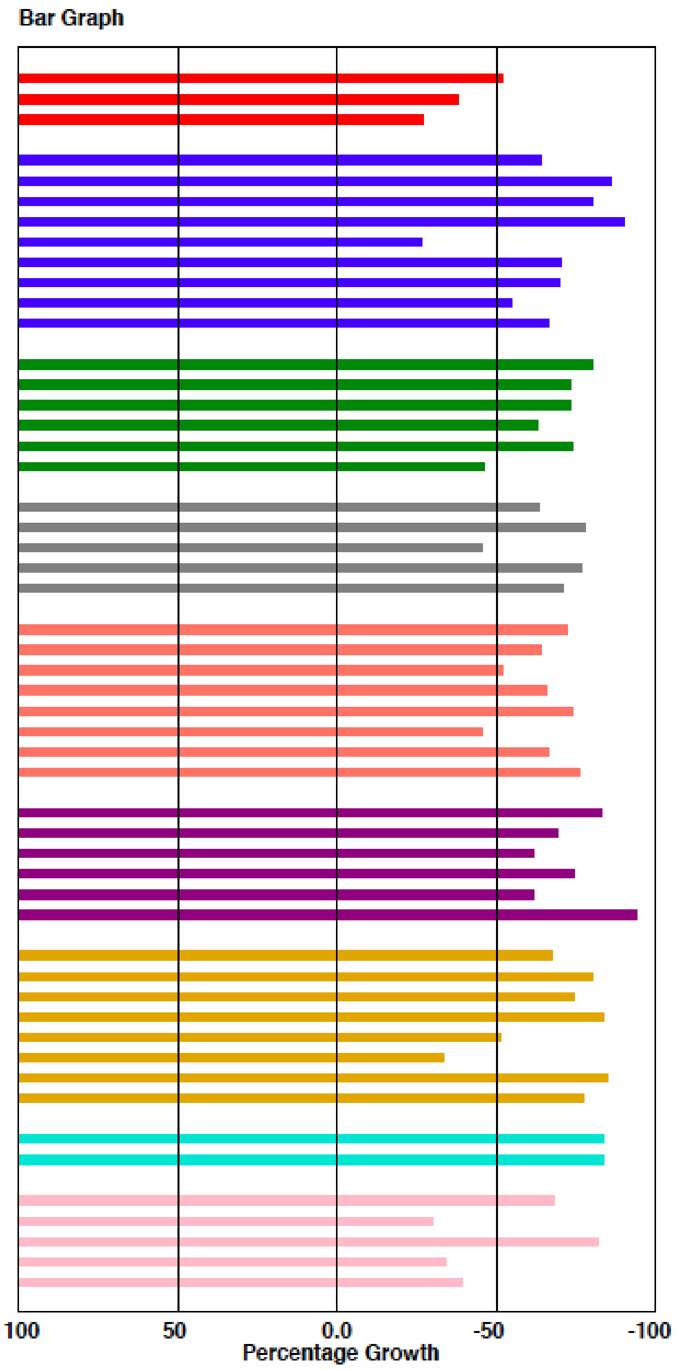

(S2) NCI-60 single dose bar graph of *S. crassifolia* root wood extract.

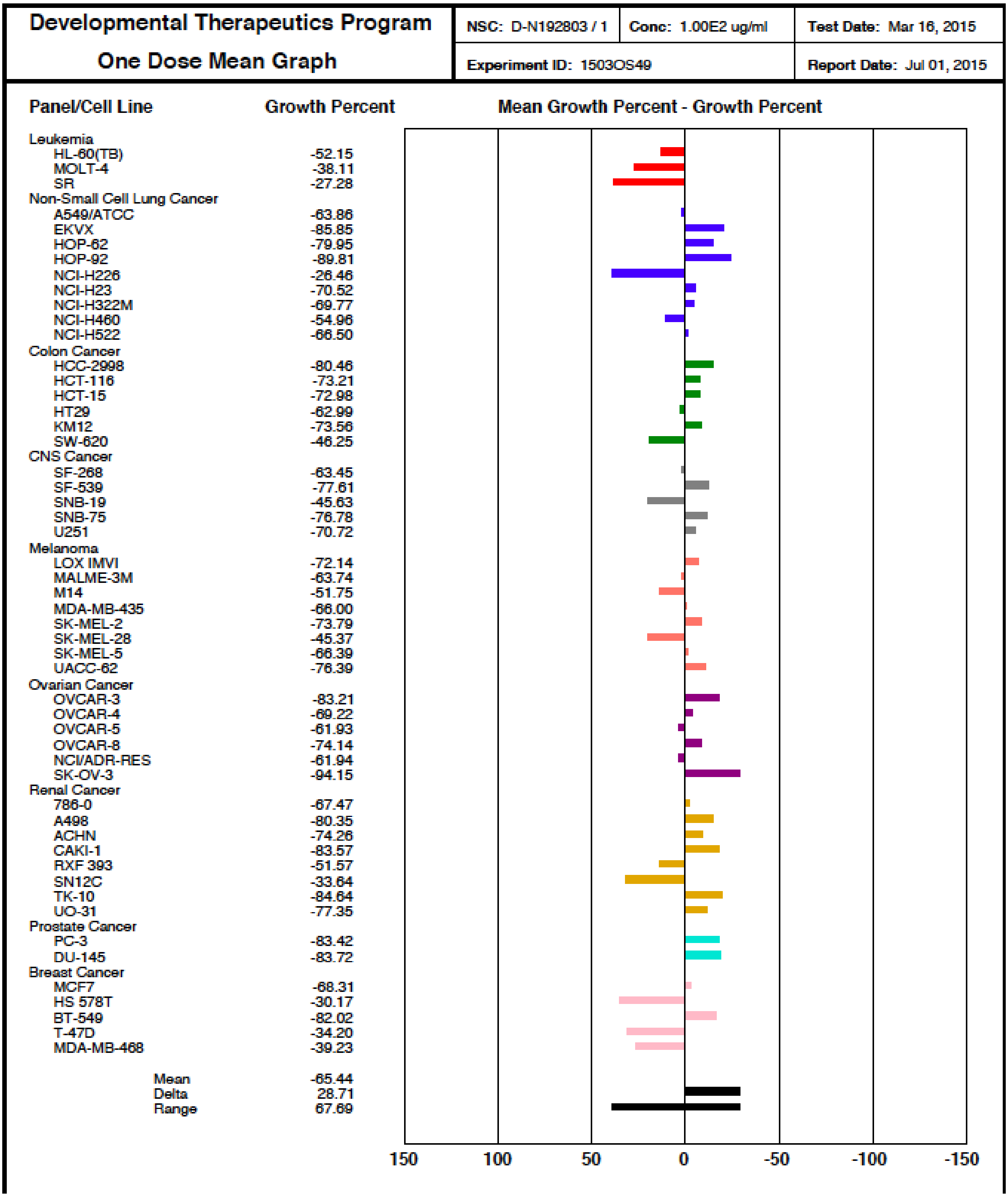

(S3) NCI-60 single dose mean bar graph of *S. crassifolia* root wood extract.

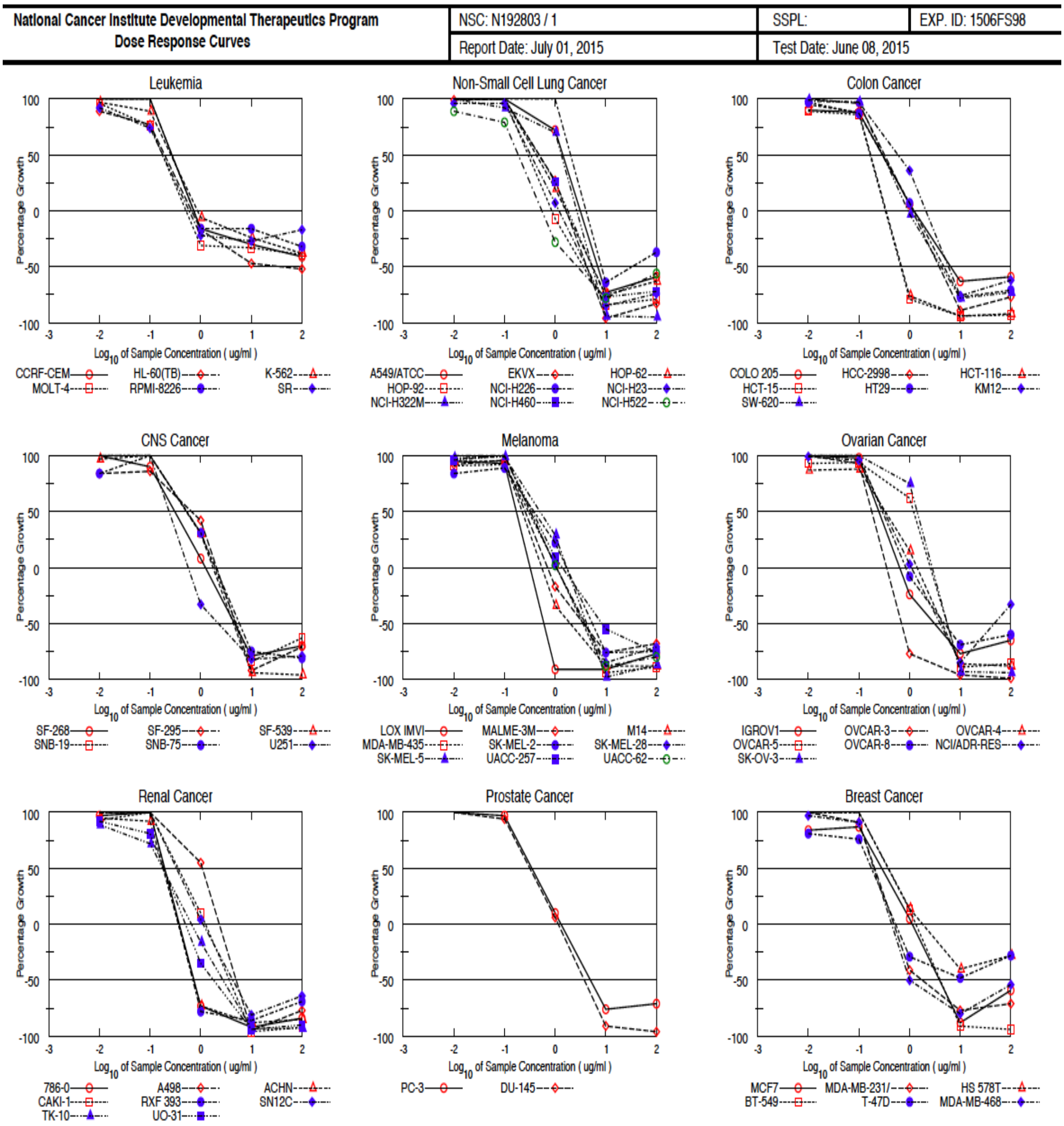

(S4) NCI-60 dose response curves for *S. crassifolia* root wood extract.

National Cancer Institute Developmental Therapeutics Program Mean Graphs	NSC :N192803/1	Units :ug/ml	SSPL :	EXP. ID :1506FS98
	Report Date :July 01, 2015		Test Date :June 08, 2015	

Panel/Cell Line	Log_{10}GI50	Log_{10}TGI	Log_{10}LC50
**Leukemia**			
CCRF-CEM	-0.56	-0.14	> 2.00
HL-60(TB)	-0.71	-0.18	1.64
K-562	-0.59	-0.06	> 2.00
MOLT-4	-0.76	-0.29	> 2.00
RPMI-8226	-0.57	-0.14	> 2.00
SR	-0.75	-0.23	> 2.00
**Non-Small Cell Lung Cancer**			
A549/ATCC	0.15	0.50	0.84
EKVX	-0.29	0.22	0.63
HOP-62	-0.34	0.21	0.74
HOP-92	-0.46	-0.06	0.55
NCI-H226	0.36	0.64	
NCI-H23	-0.49	0.07	0.62
NCI-H322M	0.12	0.43	0.73
NCI-H460	-0.32	0.25	0.74
NCI-H522	-0.73	-0.26	0.44
**Colon Cancer**			
COLO 205	-0.54	0.08	0.81
HCC-2998	-0.49	0.05	0.58
HCT-116	-0.77	-0.46	-0.16
HCT-15	-0.78	-0.48	-0.17
HT29	-0.54	0.09	0.68
KM12	-0.23	0.32	0.77
SW-620	-0.53	-0.03	0.62
**CNS Cancer**			
SF-268	-0.51	0.10	0.67
SF-295	-0.17	0.31	0.69
SF-539	-0.26	0.24	0.65
SNB-19	-0.27	0.27	0.71
SNB-75	-0.23	0.29	0.76
U251	-0.58	-0.23	0.34
**Melanoma**			
LOX IMVI	-0.77	-0.50	-0.22
MALME-3M	-0.59	-0.15	0.57
M14	-0.65	-0.26	0.29
MDA-MB-435	-0.53	0.03	0.55
SK-MEL-2	-0.42	0.22	0.73
SK-MEL-28	-0.47	0.02	0.60
SK-MEL-5	-0.30	0.23	0.62
UACC-257	-0.43	0.15	0.93
UACC-62	-0.48	0.03	0.58
**Ovarian Cancer**			
IGROV1	-0.61	-0.20	0.49
OVCAR-3	-0.75	-0.45	-0.16
OVCAR-4	-0.48	0.15	0.64
OVCAR-5	0.08	0.41	0.74
OVCAR-8	-0.50	-0.07	0.69
NCI/ADR-RES	-0.50	0.03	
SK-OV-3	0.15	0.45	0.74
**Renal Cancer**			
786-0	-0.71	-0.42	-0.13
A498	0.04	0.37	0.70
ACHN	-0.74	-0.44	-0.14
CAKI-1	-0.44	0.09	0.57
RXF 393	-0.62	-0.38	-0.14
SN12C	-0.47	0.05	0.63
TK-10	-0.75	-0.18	0.44
UO-31	-0.73	-0.30	0.25
**Prostate Cancer**			
PC-3	-0.46	0.11	0.69
DU-145	-0.50	0.06	0.57
**Breast Cancer**			
MCF7	-0.55	0.05	0.59
MDA-MB-231/ATCC	-0.69	-0.31	0.26
HS 578T	-0.41	0.26	> 2.00
BT-549	-0.36	0.13	0.61
T-47D	-0.75	-0.27	> 2.00
MDA-MB-468	-0.71	-0.36	
MID	-0.47	0.0	0.7
Delta	0.31	0.5	0.92
Range	1.14	1.14	2.22

(S5) NCI-60 5-dose mean bar graph of *S. crassifolia* root wood extract.

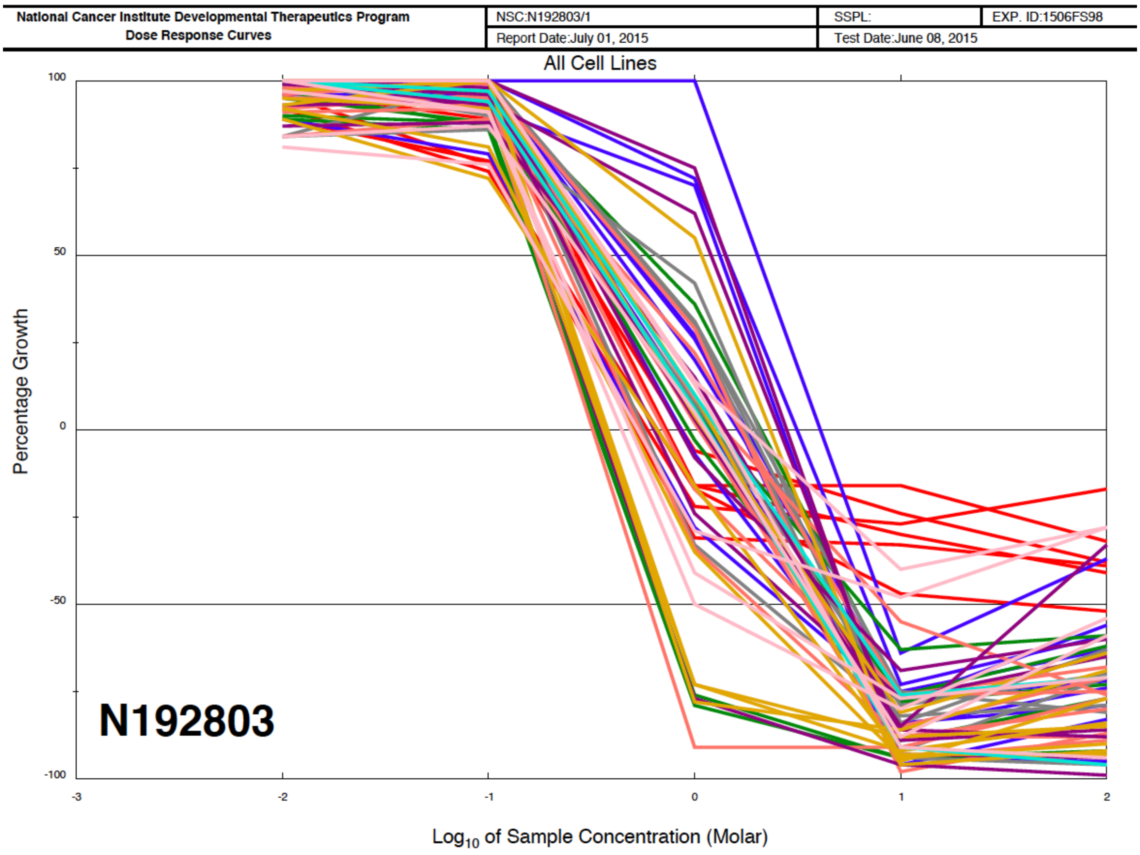

(S6) NCI-60 cumulative dose response curves for *S. crassifolia* root wood extract.

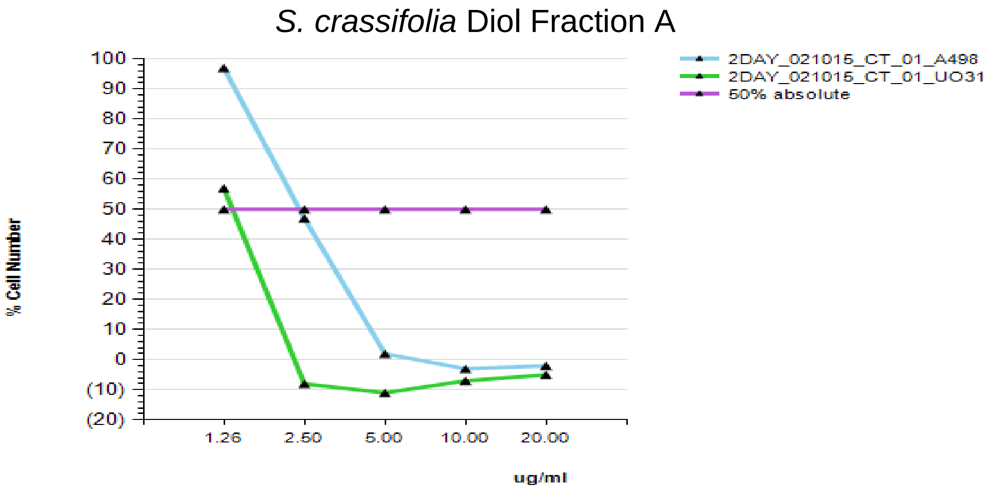
S. crassifolia Diol Fraction A
2DAY_021015_CT_01_A498
2DAY_021015_CT_01_UO31
50% absolute
% Cell Number
100
90
80
70
60
50
40
30
20
10
0
(10)
(20)
1.26
2.50
5.00
10.00
20.00
ug/ml

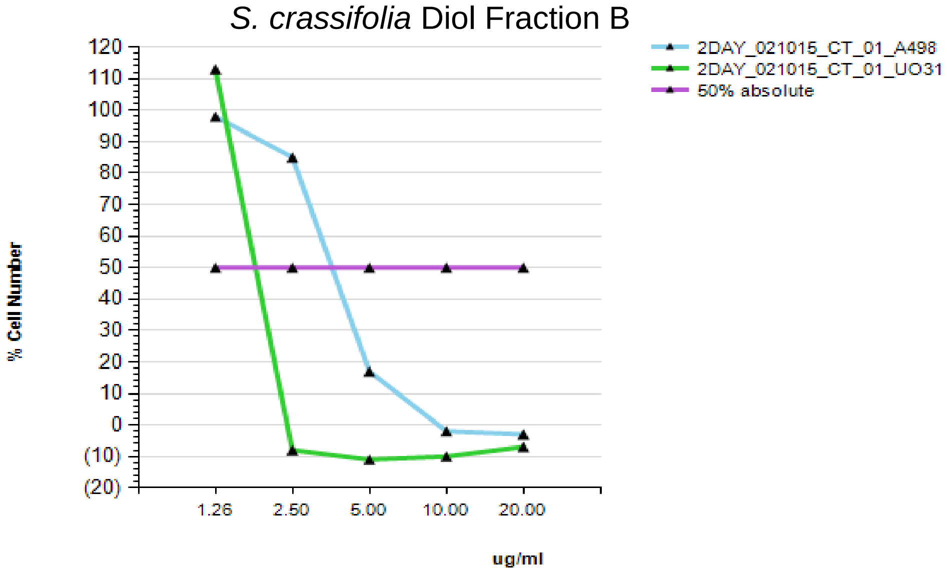
S. crassifolia Diol Fraction B
2DAY_021015_CT_01_A498
2DAY_021015_CT_01_UO31
50% absolute
% Cell Number
120
110
100
90
80
70
60
50
40
30
20
10
0
(10)
(20)
1.26
2.50
5.00
10.00
20.00
ug/ml

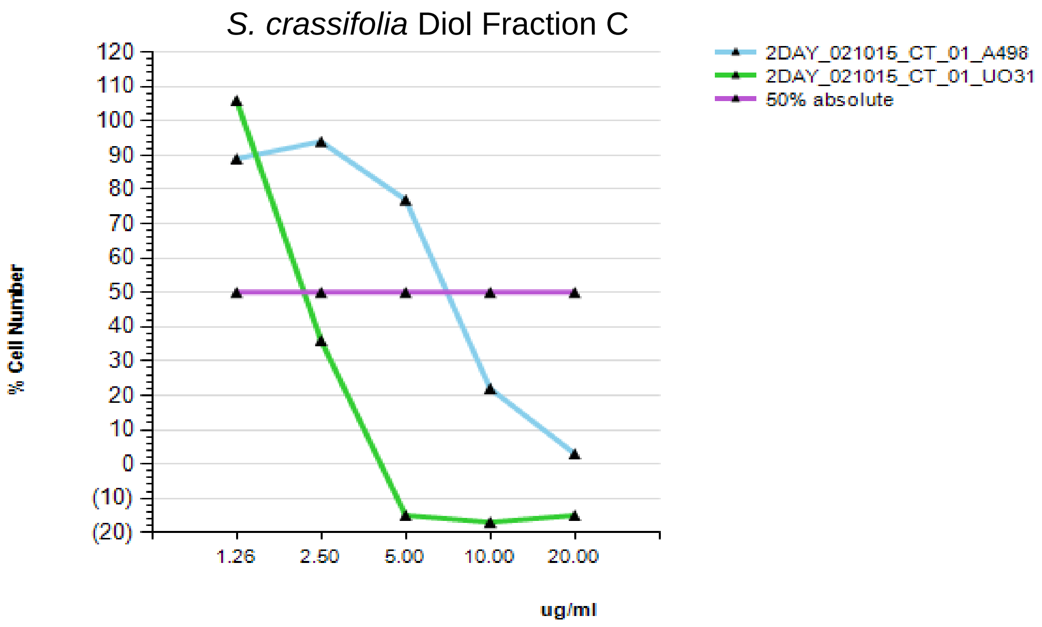

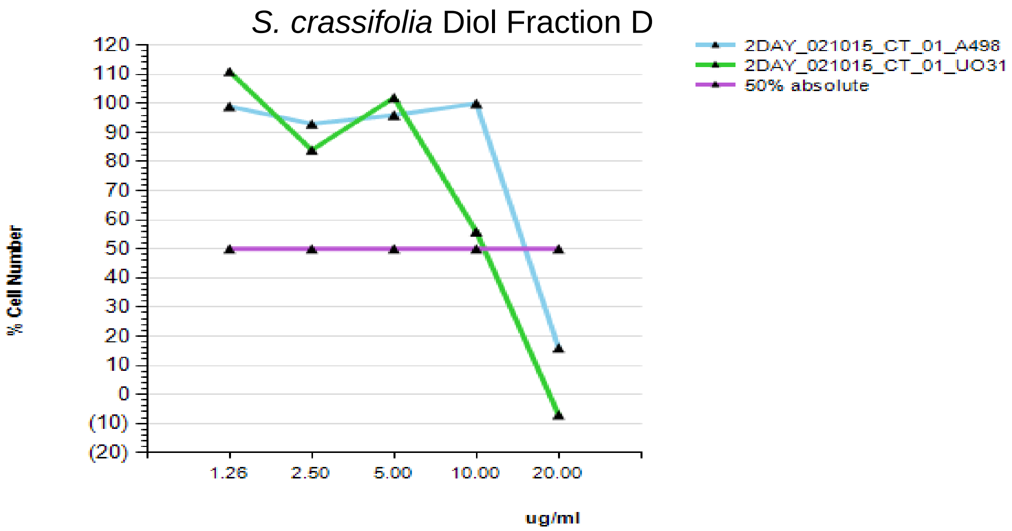

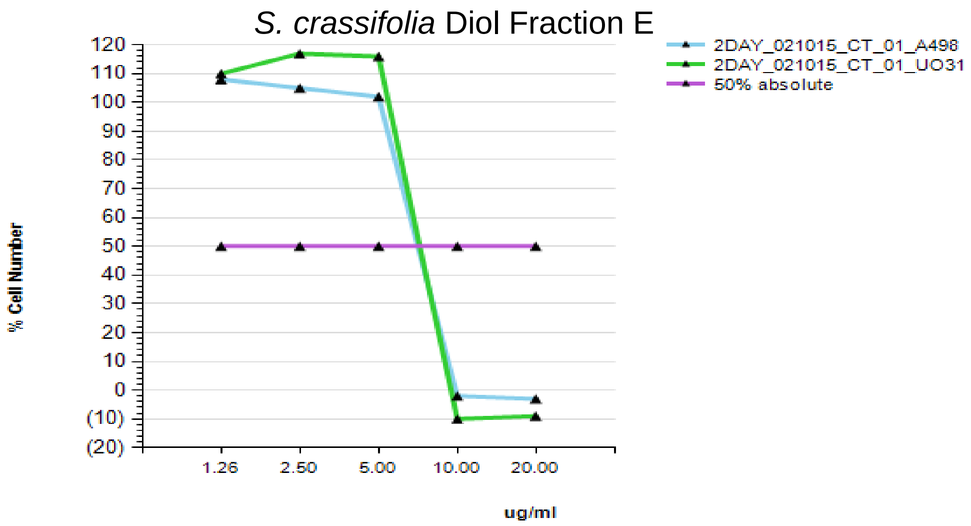

(S7) Cytotoxicity of DIOL fractions A-E of *S. crassifolia* root wood hexane extract in renal A498 and U031 cancer cell lines.

S. *crassifolia* Diol Fraction A

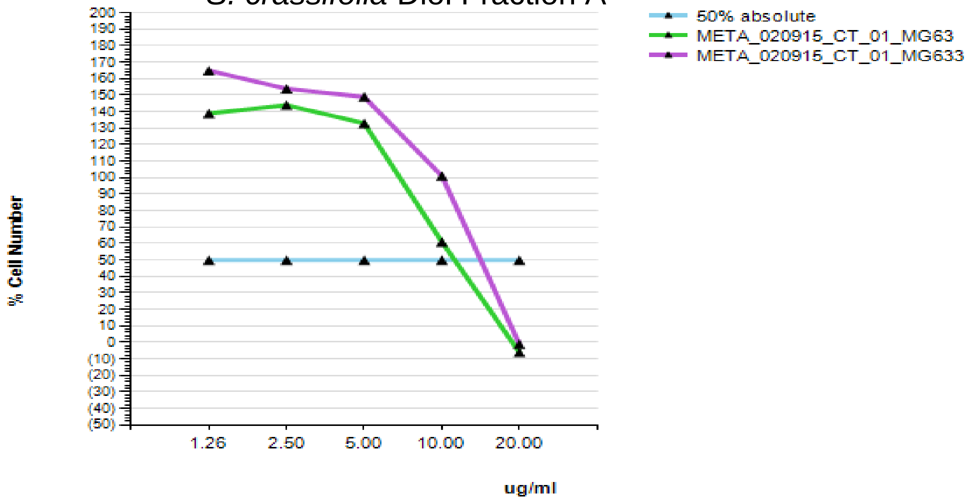

S. *crassifolia* Diol Fraction B

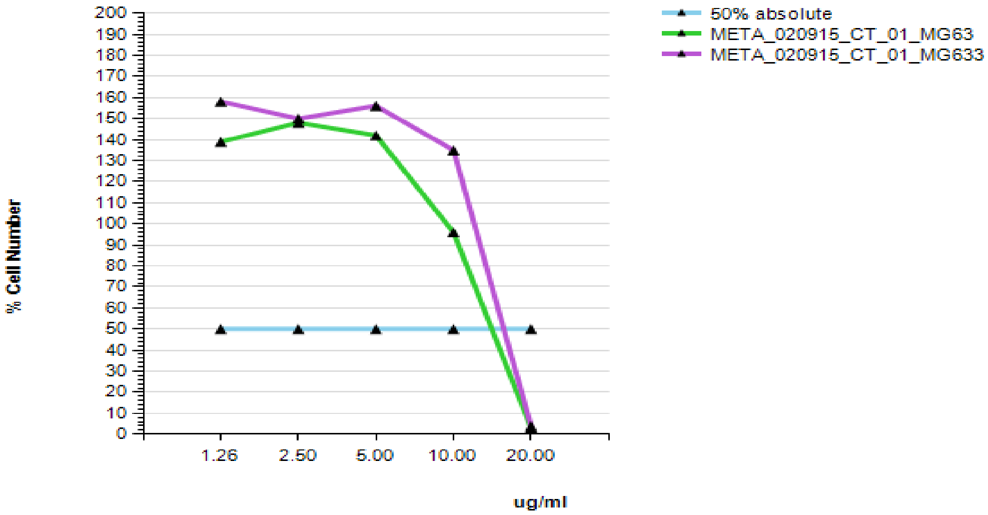

S. crassifolia Diol Fraction C

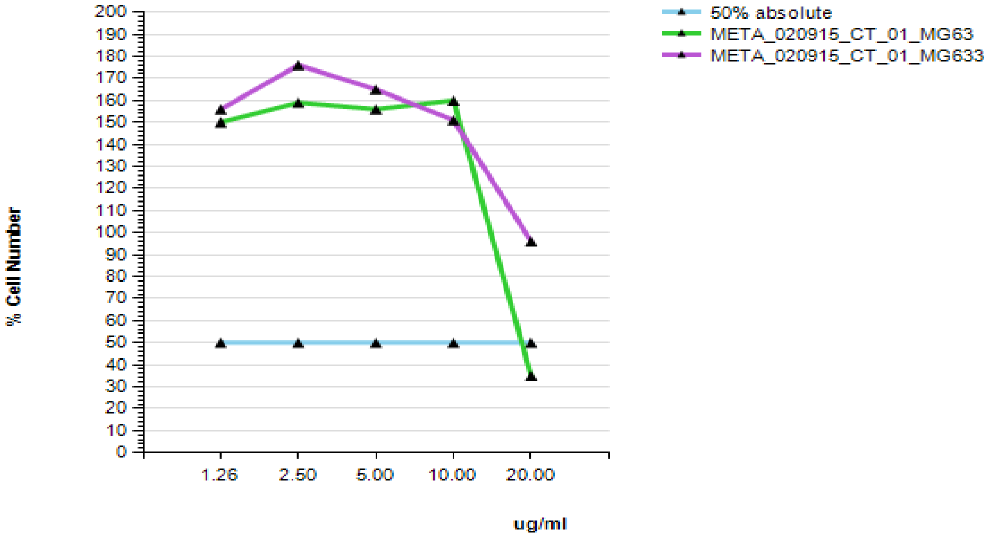

S. crassifolia Diol Fraction D

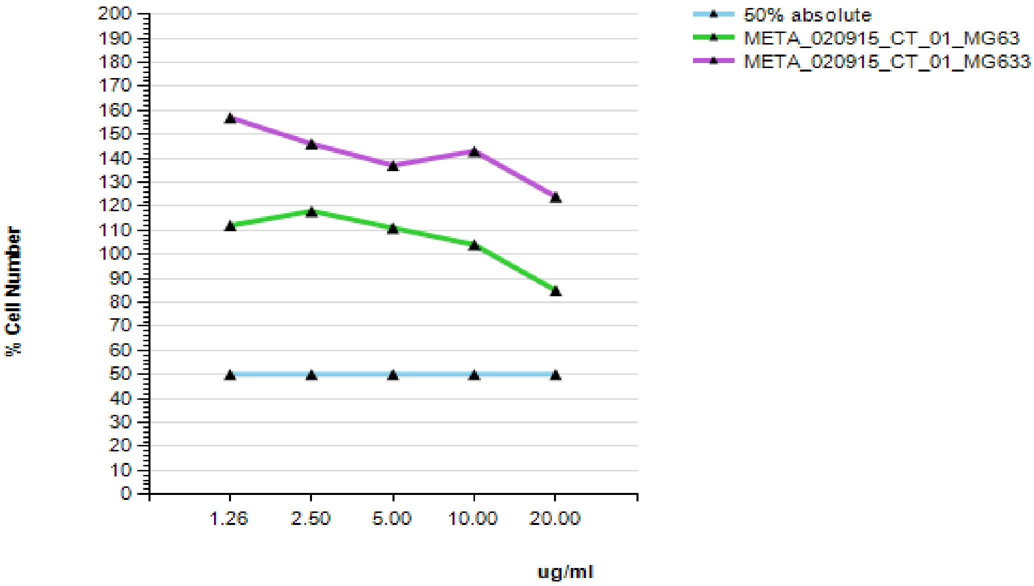

S. crassifolia Diol Fraction E

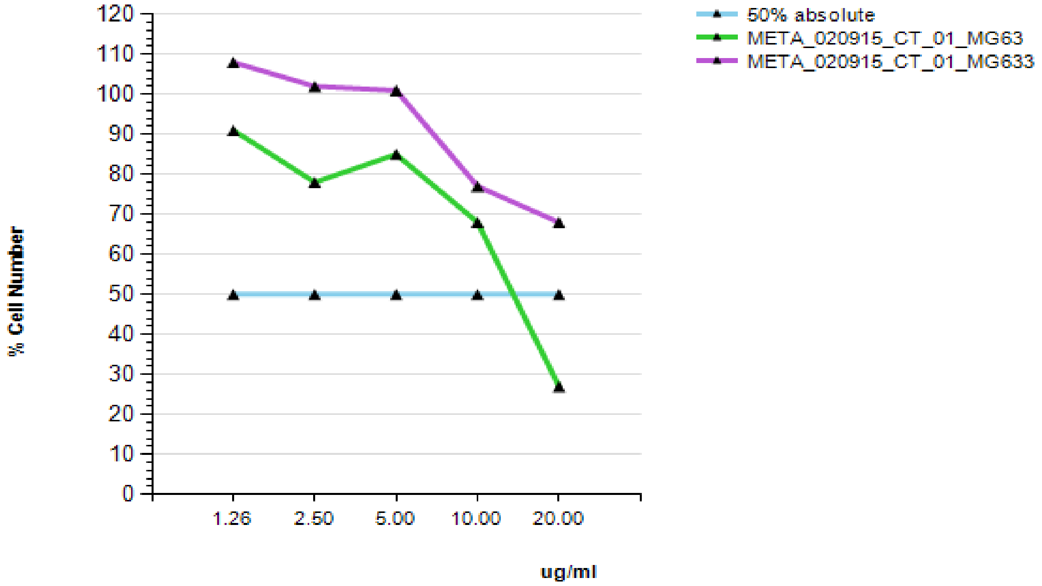

(S8) Cytotoxicity of DIOL fractions A-E of *S. crassifolia* root wood hexane extract in osteosarcoma MG63 and MG63.3 cell lines.

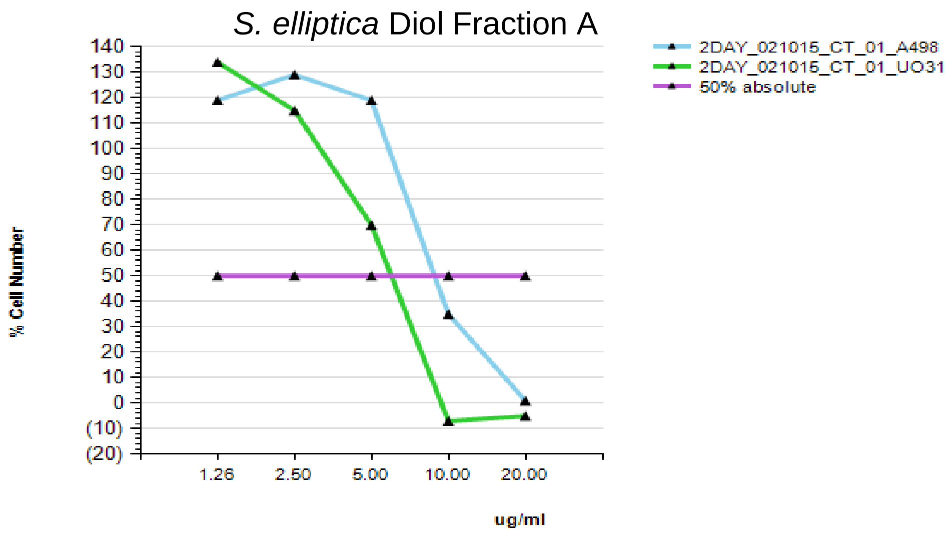

S. elliptica Diol Fraction B

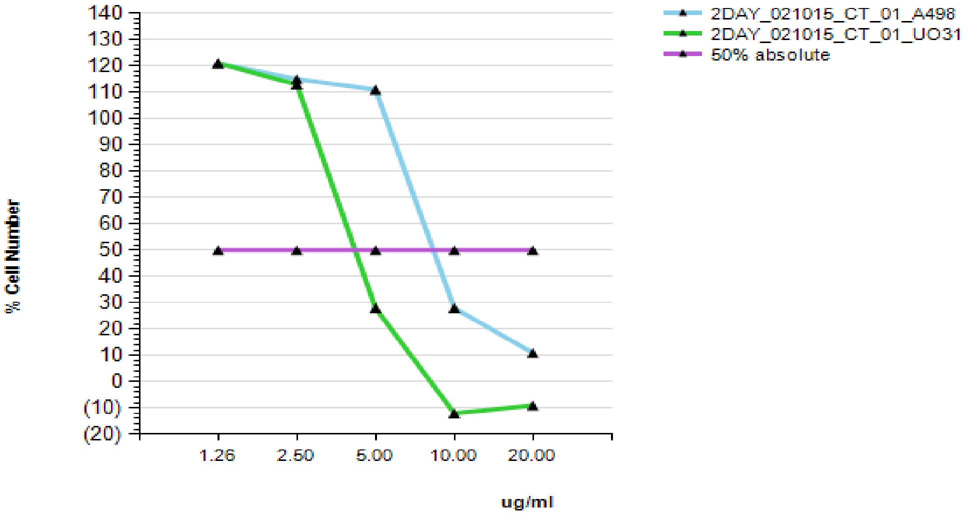

S. elliptica Diol Fraction C

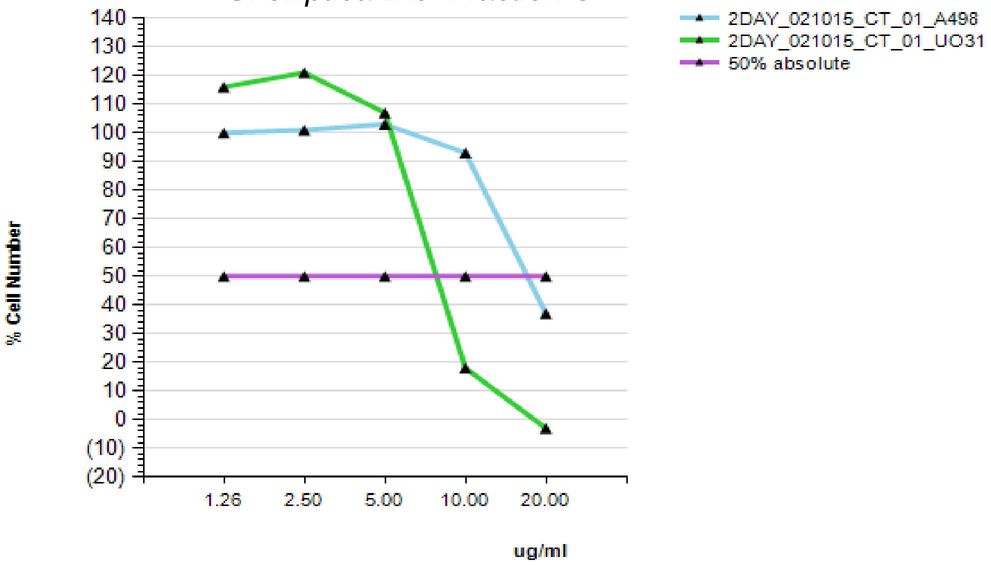

S. elliptica Diol Fraction D

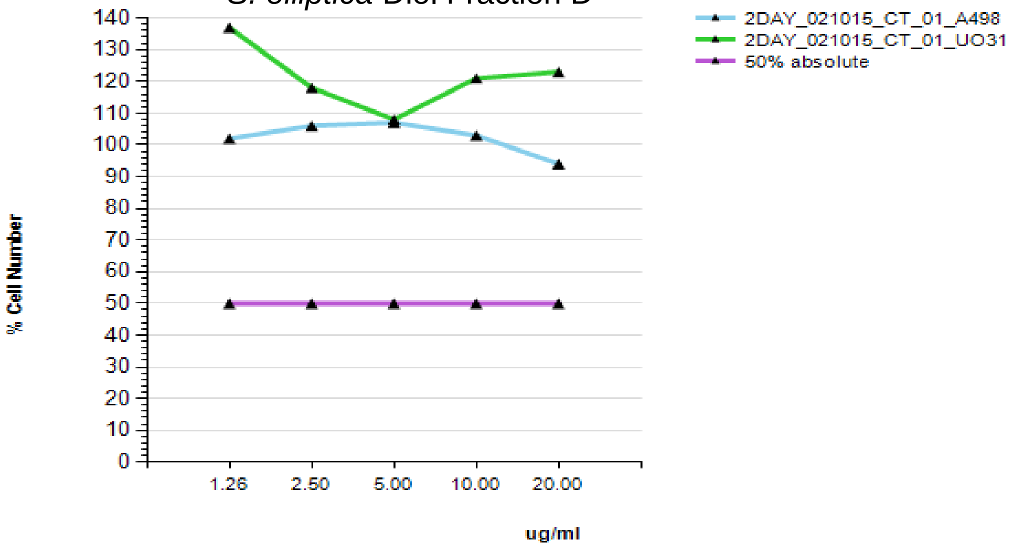

S. elliptica Diol Fraction E

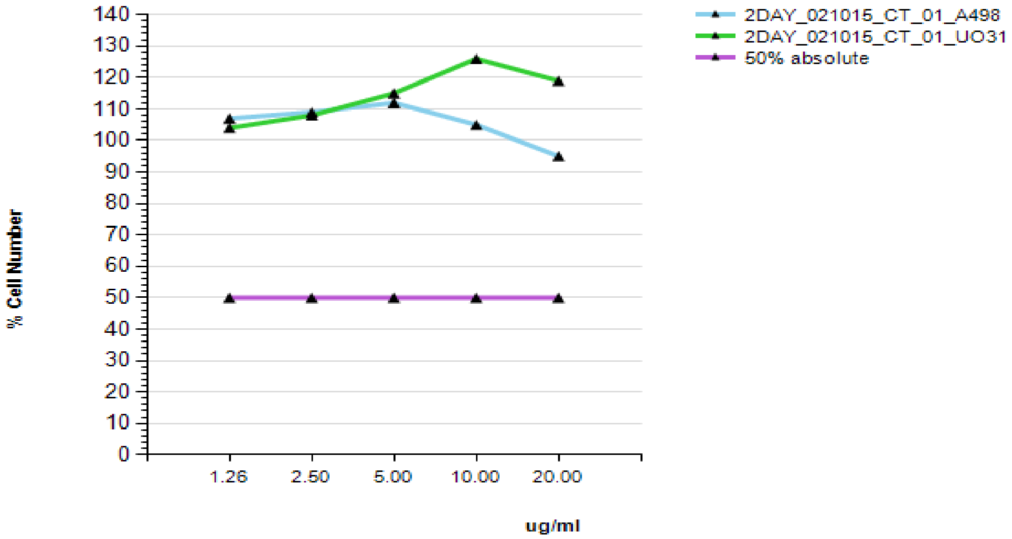

(S9) Cytotoxicity of DIOL fractions A-E of *S. elliptica* root wood ethyl acetate extract in renal A498 and U031 cancer cell lines.

S. elliptica Diol Fraction A

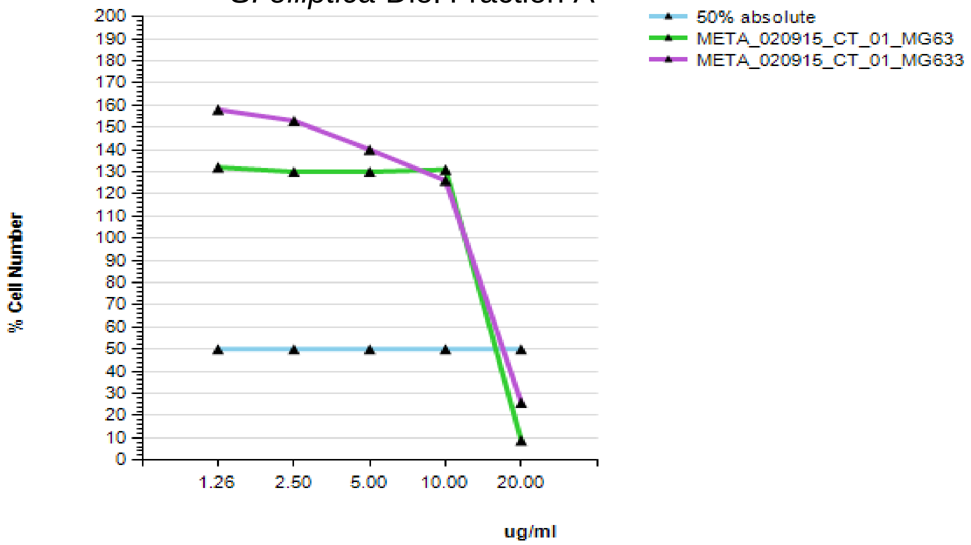

S. elliptica Diol Fraction B

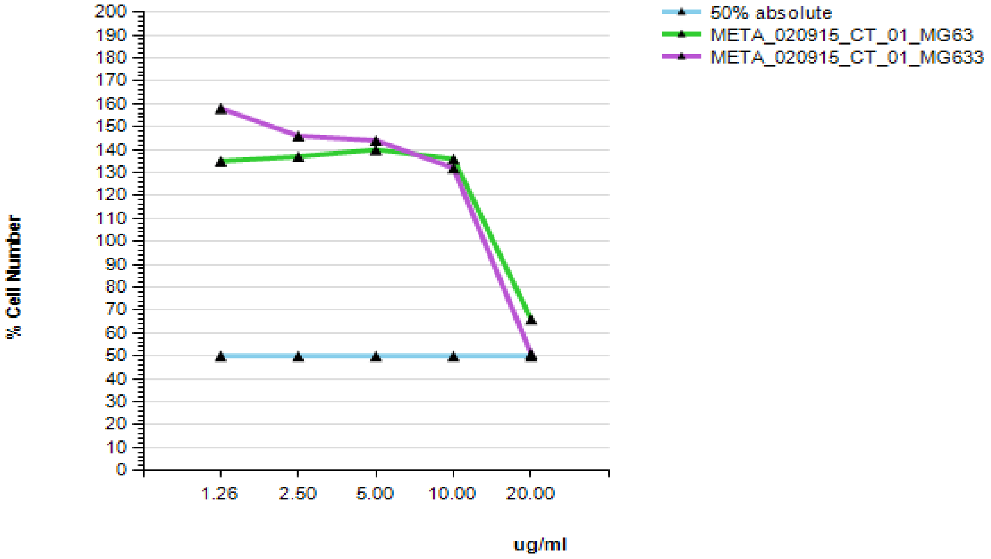

S. elliptica Diol Fraction C

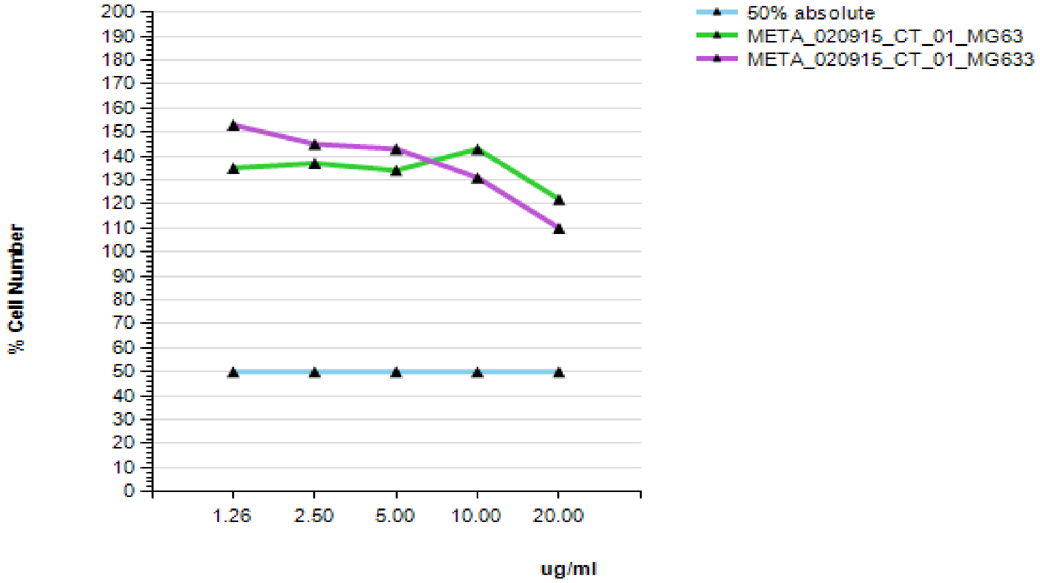

S. elliptica Diol Fraction D

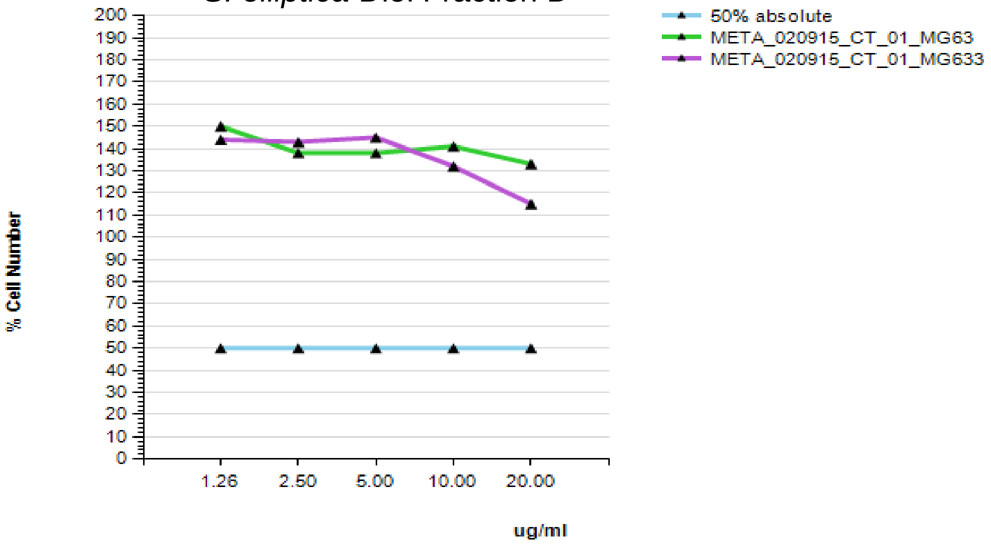

S. elliptica Diol Fraction E

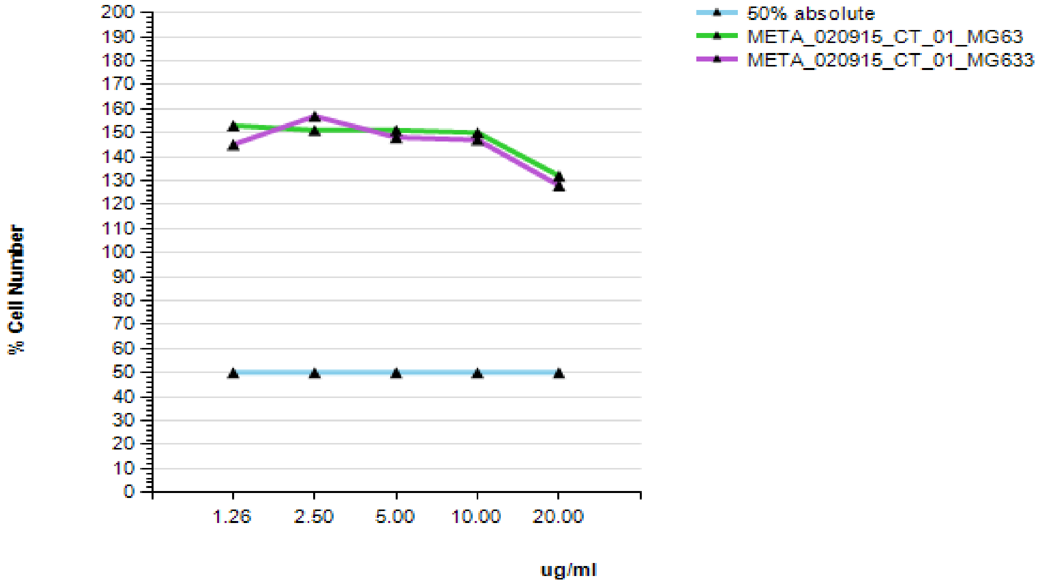

(S10) Cytotoxicity of DIOL fractions A-E of *S. elliptica* root wood ethyl acetate extract in osteosarcoma MG63 and MG63.3 cell lines.

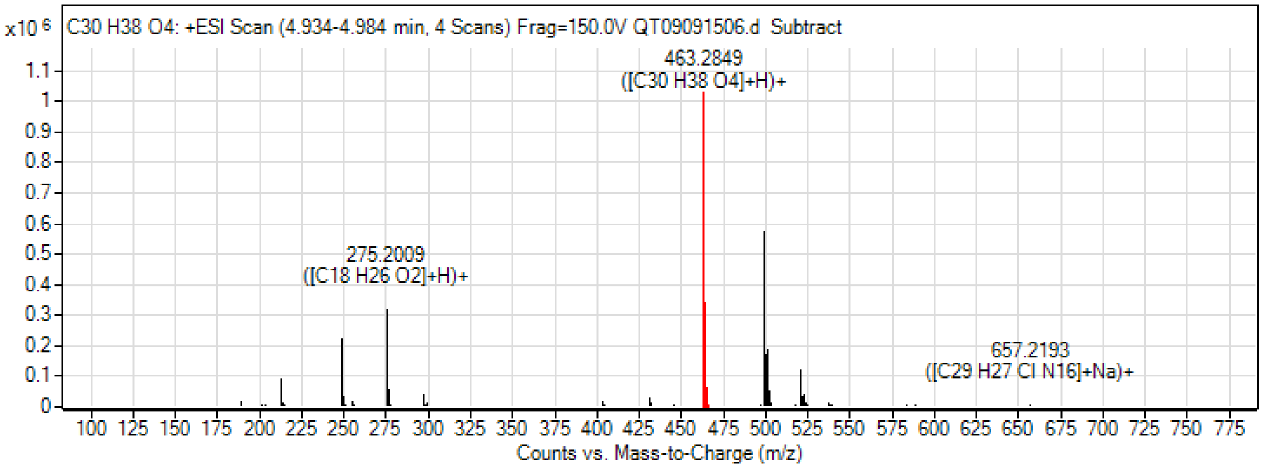

(S11) HRESIMS spectrum of 11β-hydroxypristimerin (**1**).

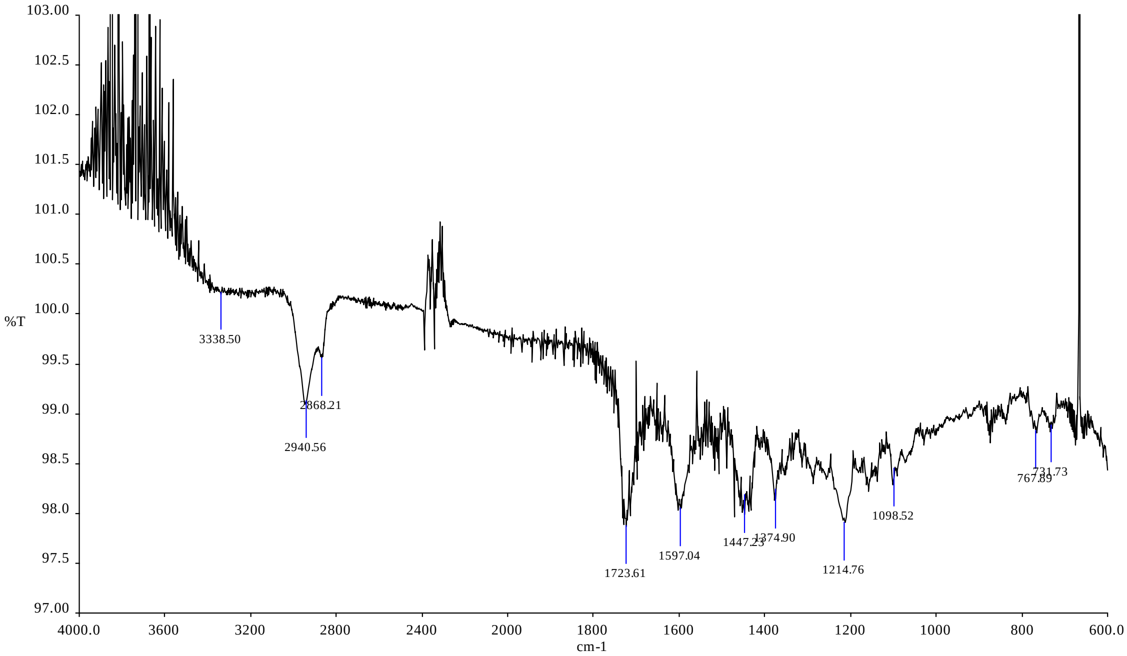

(S12) Infrared spectrum of 11β-hydroxypristimerin (**1**).

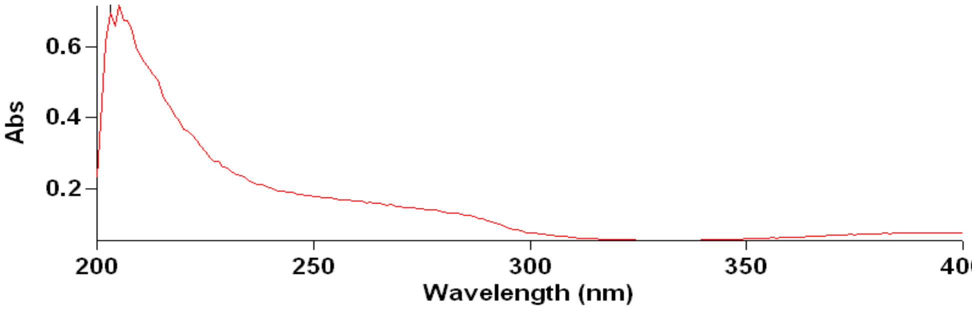

(S13) UV spectrum of 11β-hydroxypristimerin (**1**).

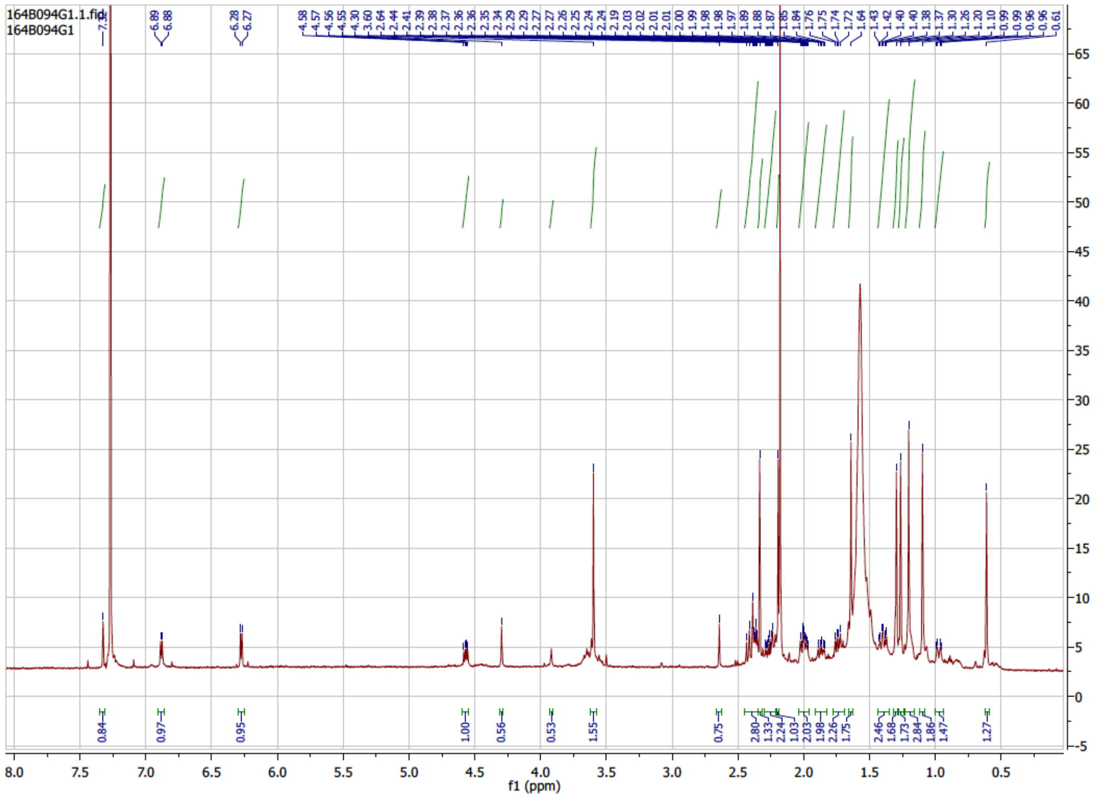

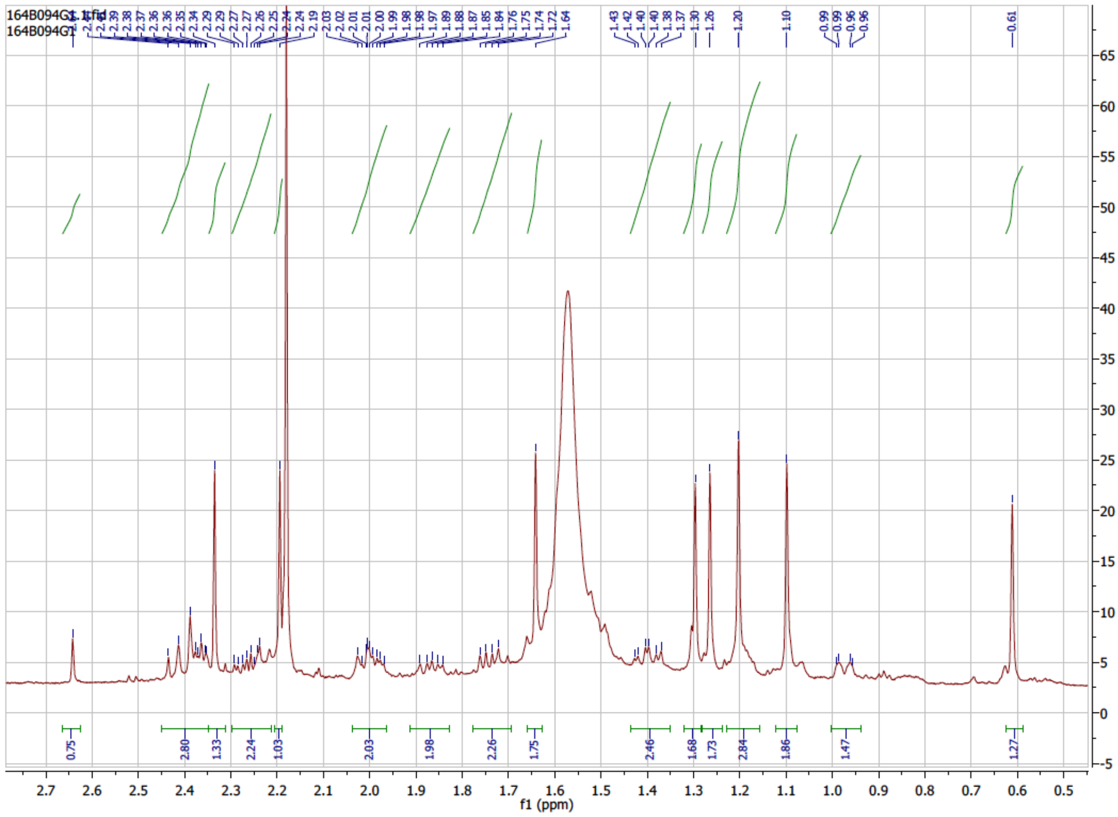

(S14) ^{1}H NMR spectrum (600 MHz) of 11β-hydroxypristimerin (**1**) in $CDCl_3$.

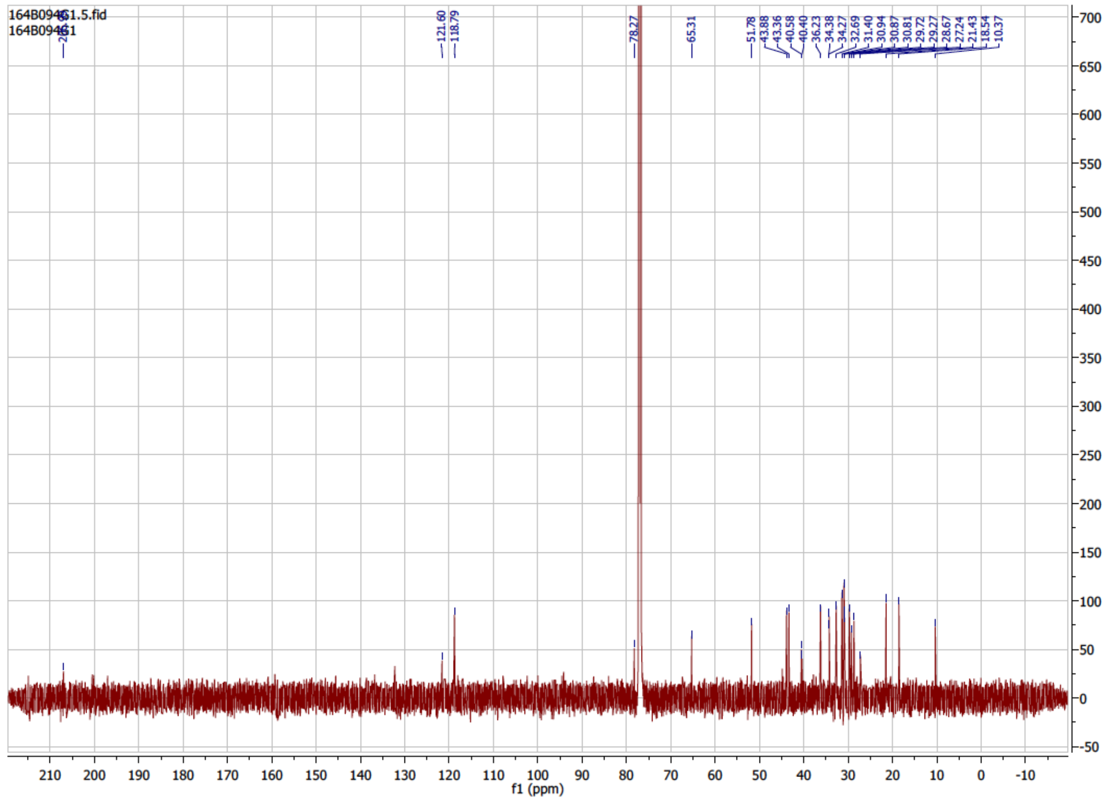

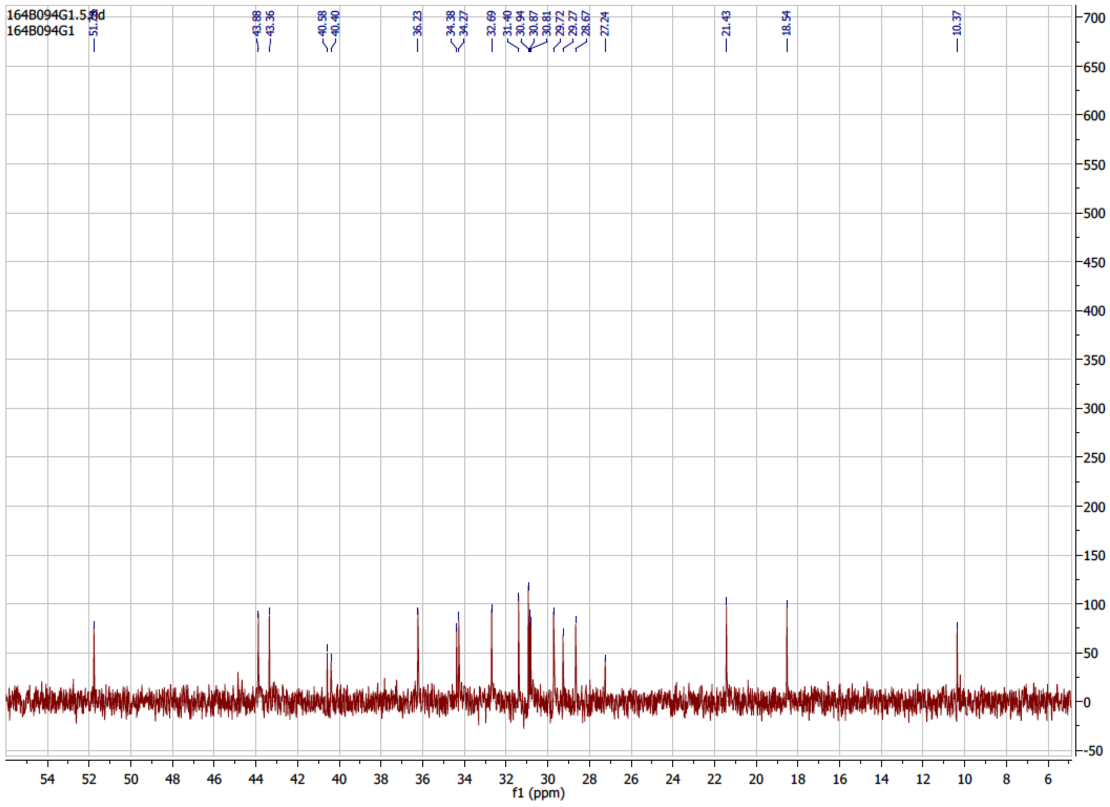

(S15) ^{13}C NMR spectrum (150 MHz) of 11*β*-hydroxypristimerin (**1**) in $CDCl_3$.

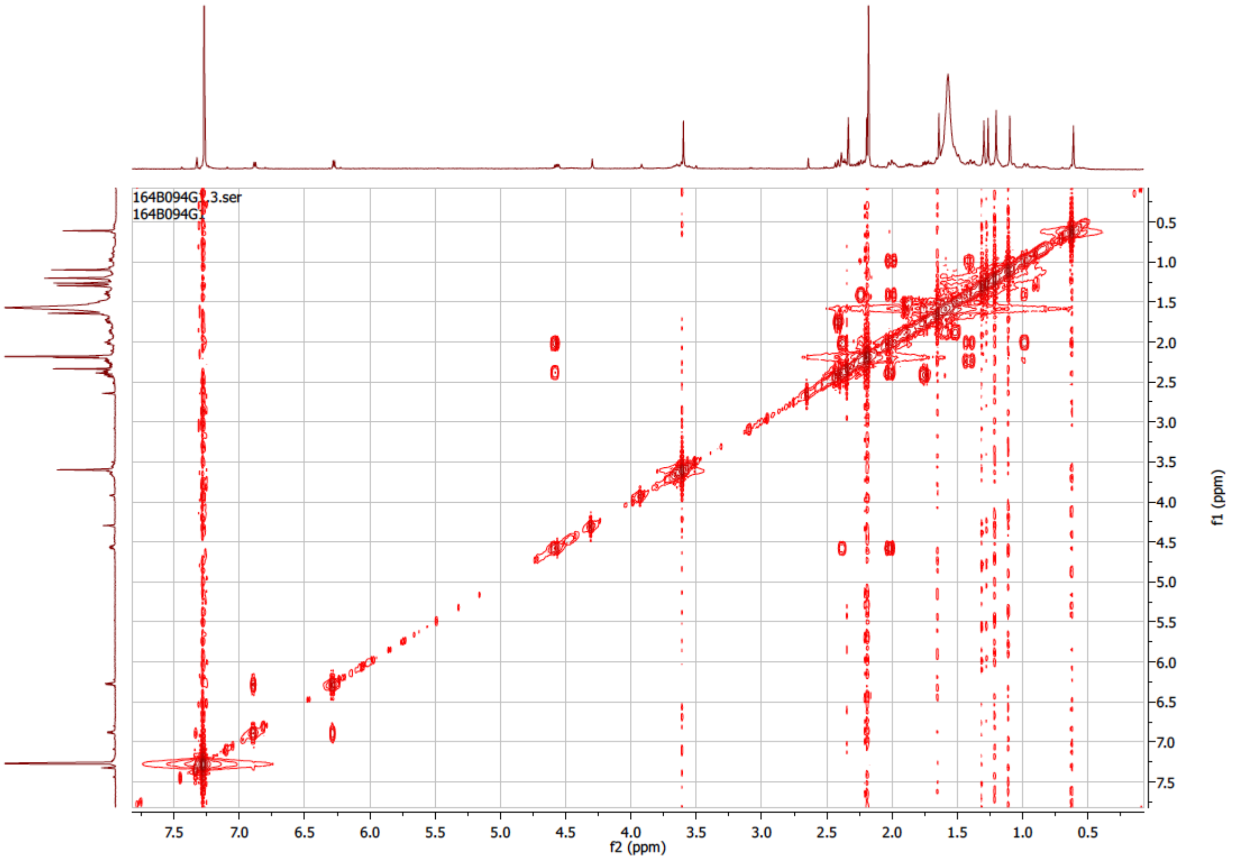
164B094G1.3.ser
164B094G1
f2 (ppm)
f1 (ppm)

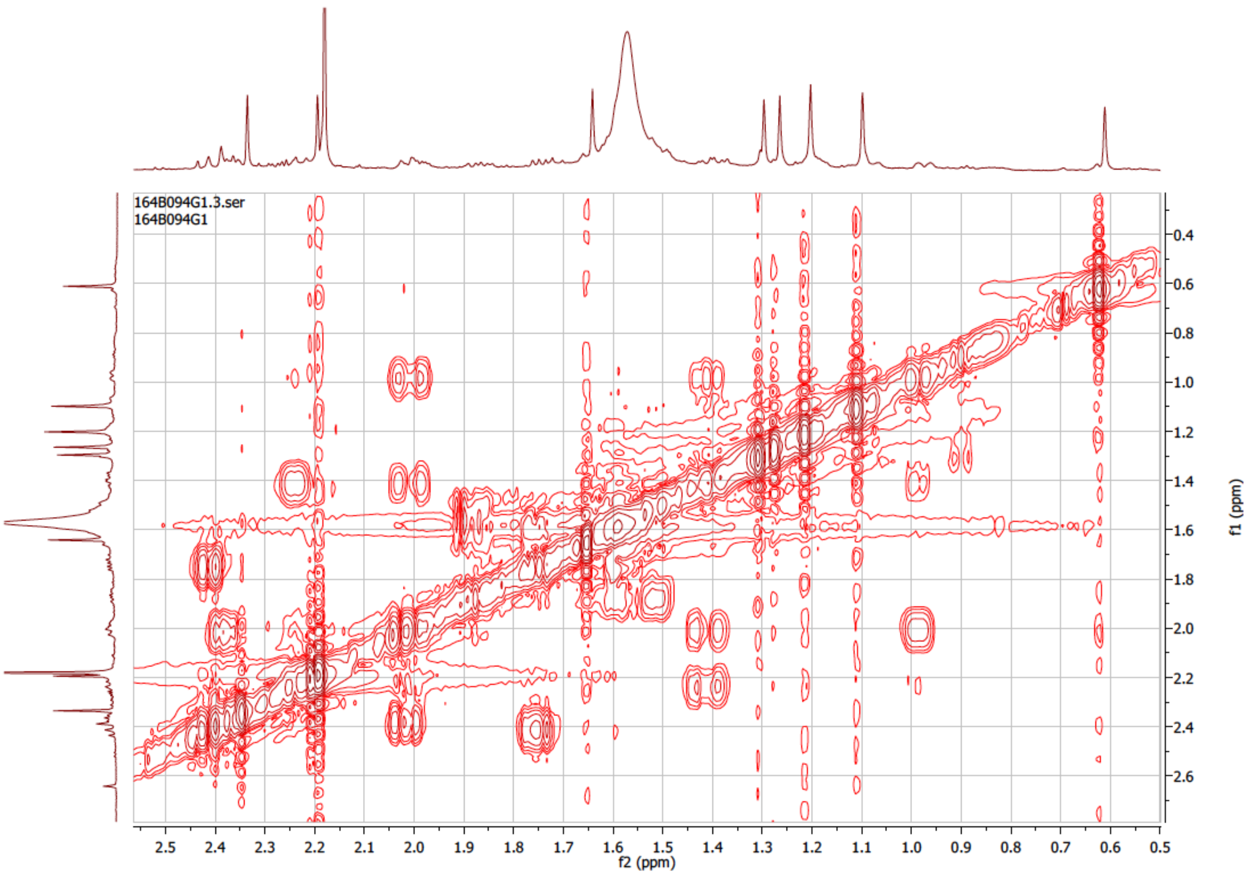

(S16) COSY spectrum of 11*β*-hydroxypristimerin (**1**) in $CDCl_3$.

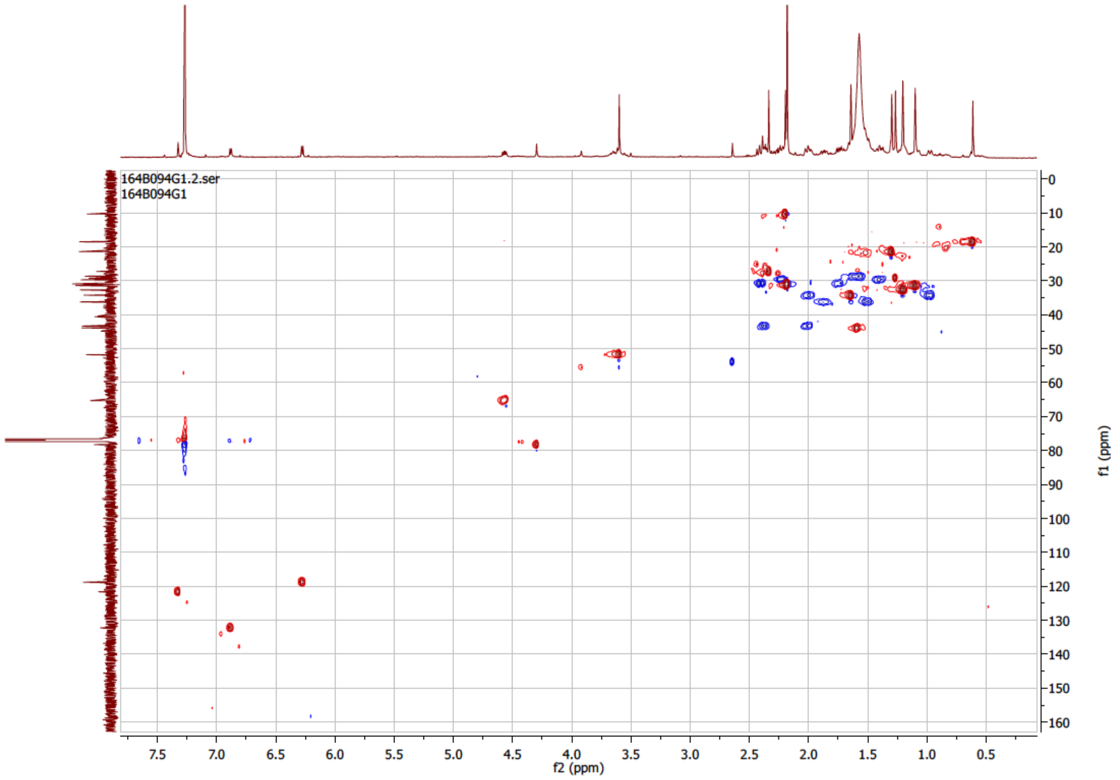

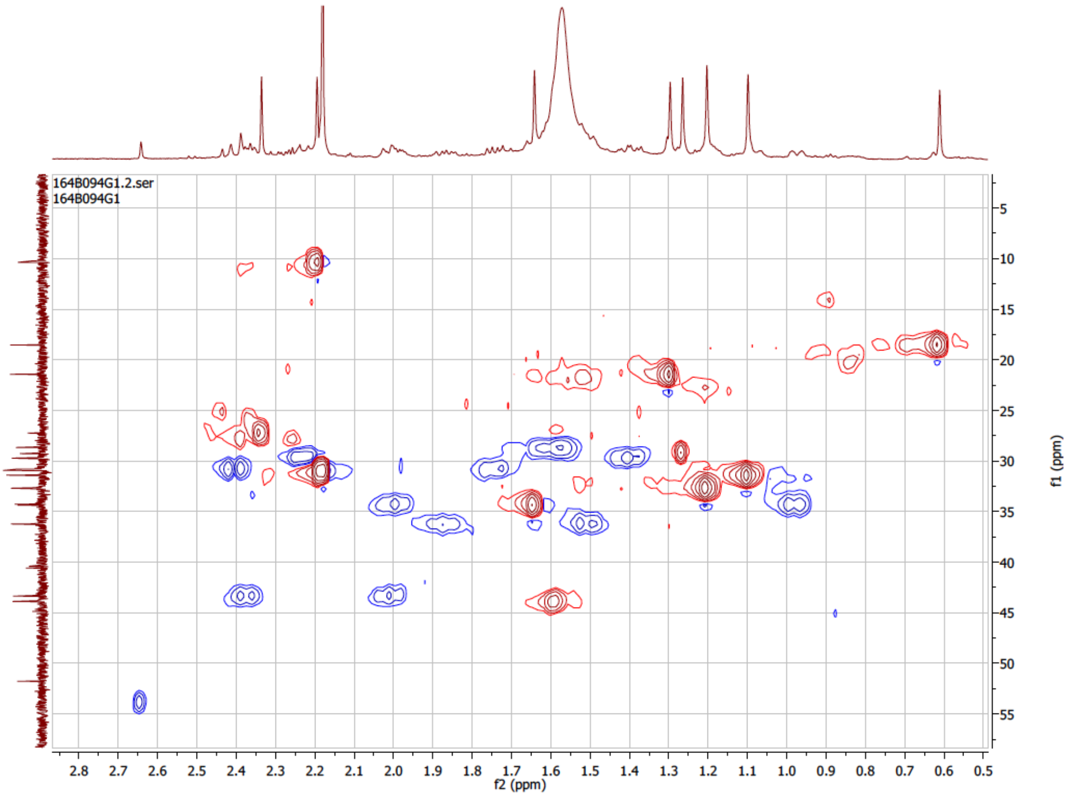

(S17) HSQC spectrum of 11*β*-hydroxypristimerin (**1**) in $CDCl_3$.

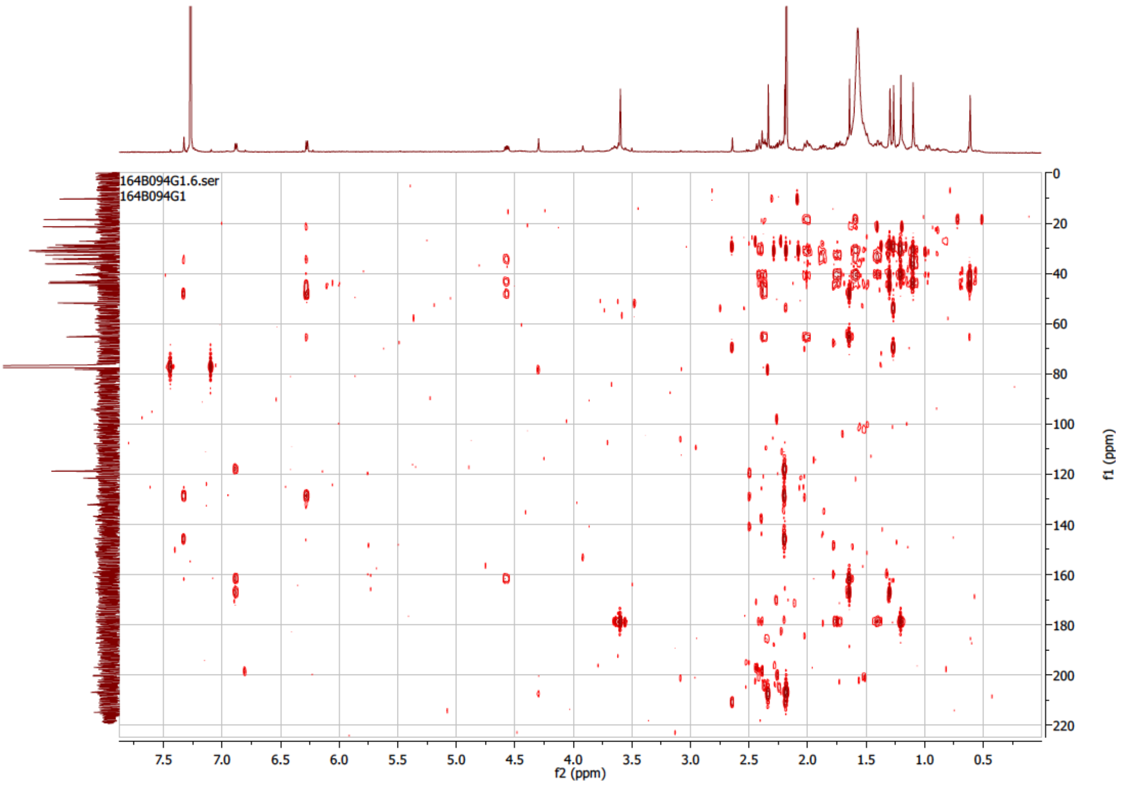

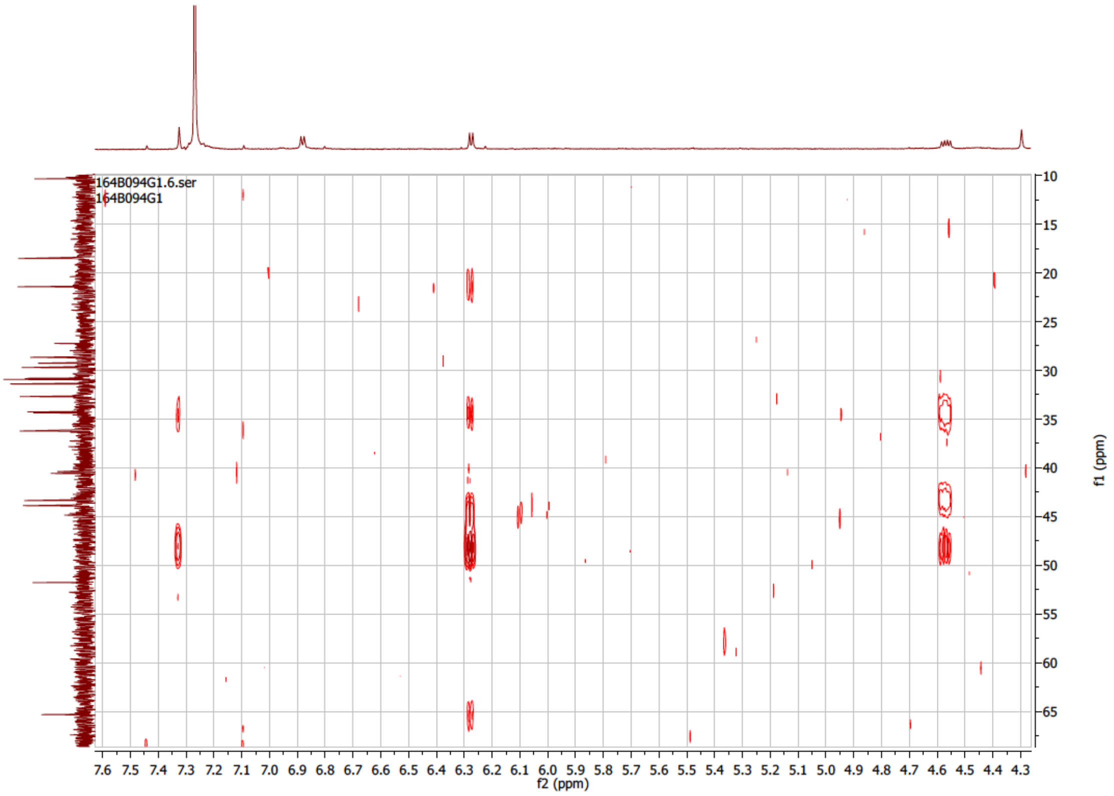

(S18) HMBC spectrum of 11*β*-hydroxypristimerin (**1**) in $CDCl_3$.

	**11*β*-Hydroxypristimerin (Compound 1)**	
	C	H
**C**		
2	178.8	-
3	145.9	-
4	118.0	-
5	128.6	-
8	167.2	-
9	48.1	-
10	161.6	-
13	40.6	-
14	44.9	-
17	30.87	-
20	40.4	-
29	178.7	-
**CH**		

1	121.6	7.32 (s)
6	132.2	6.88 (d, 7.2)
7	118.8	6.28 (d, 7.2)
11	65.3	4.57 (dd, 12.2, 6.4)
18	43.9	1.59 (m)
**CH_2**		
12	43.4	2.38 (m); 2.01 (m)
15	28.7	1.62 (m); 1.58 (m)
16	36.2	1.87 (m); 1.51 (m)
19	30.81	2.40 (m); 1.74 (m)
21	29.7	2.24 (m); 1.40 (m)
22	34.3	2.00 (m); 0.98 (m)
23	10.4	2.19 (s)
**CH_3**		
25	34.4	1.64 (s)
26	21.4	1.30 (s)
27	18.5	0.61 (s)
28	31.4	1.10 (s)
30	32.7	1.20 (s)
MeO	51.8	3.60 (s)

(S19) NMR Spectroscopic Data (^{1}H 600 MHz, ^{13}C 150 MHz) for 11*β*-hydroxypristimerin (**1**) in $CDCl_3$.

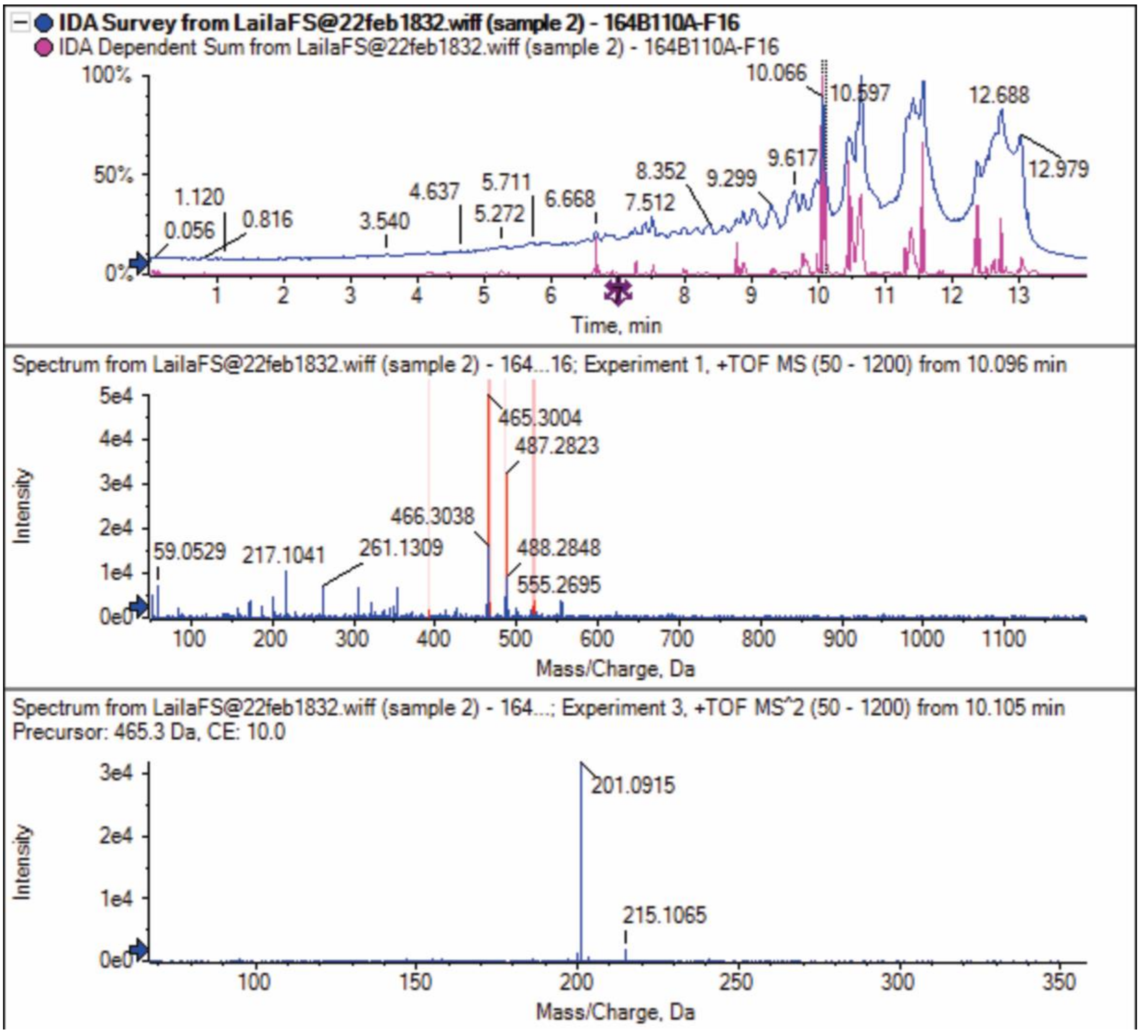

(S20) Chromatogram, MS1 and MS2 spectra of the LC-MS/MS analysis of pristimerin (**2**).

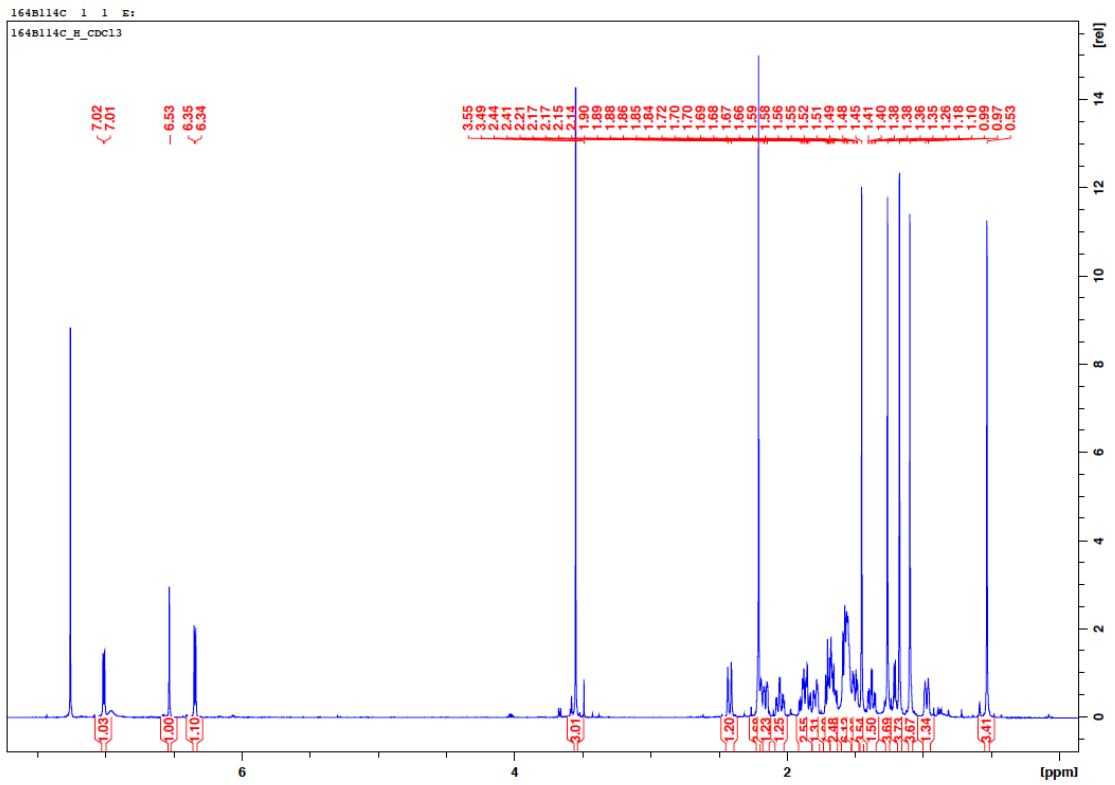

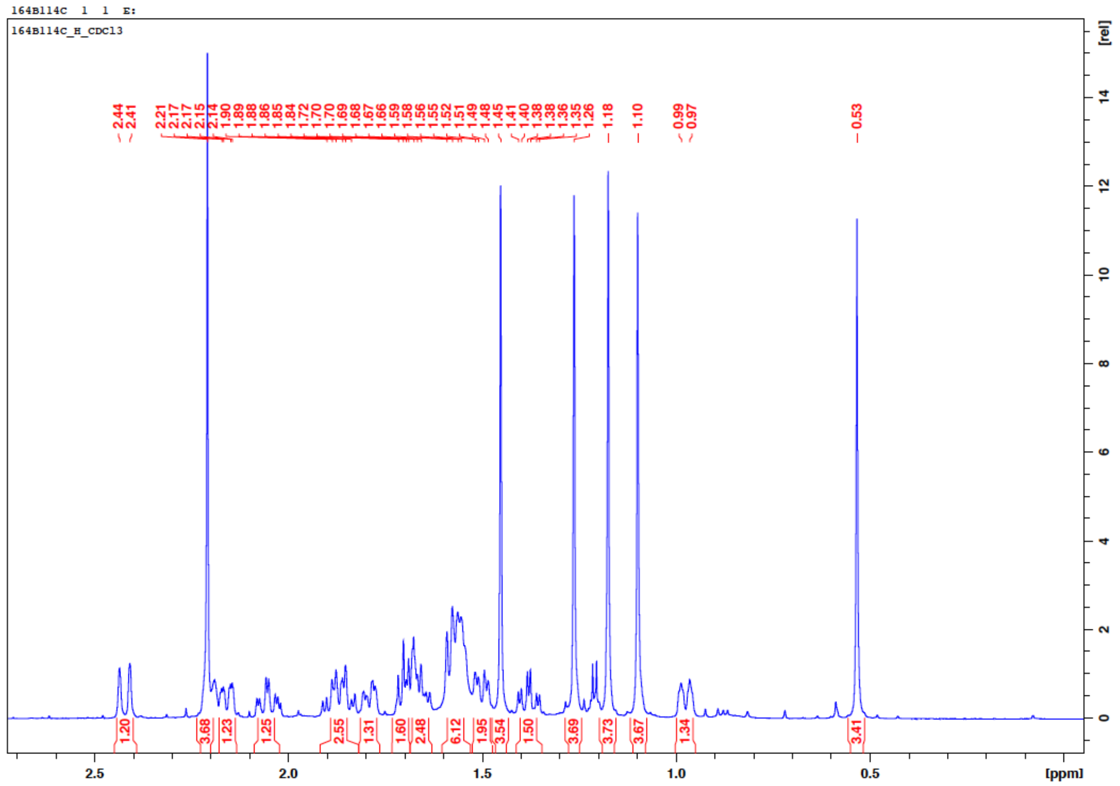

(S21) ^{1}H NMR spectrum (600 MHz) of pristimerin (**2**) in $CDCl_3$.

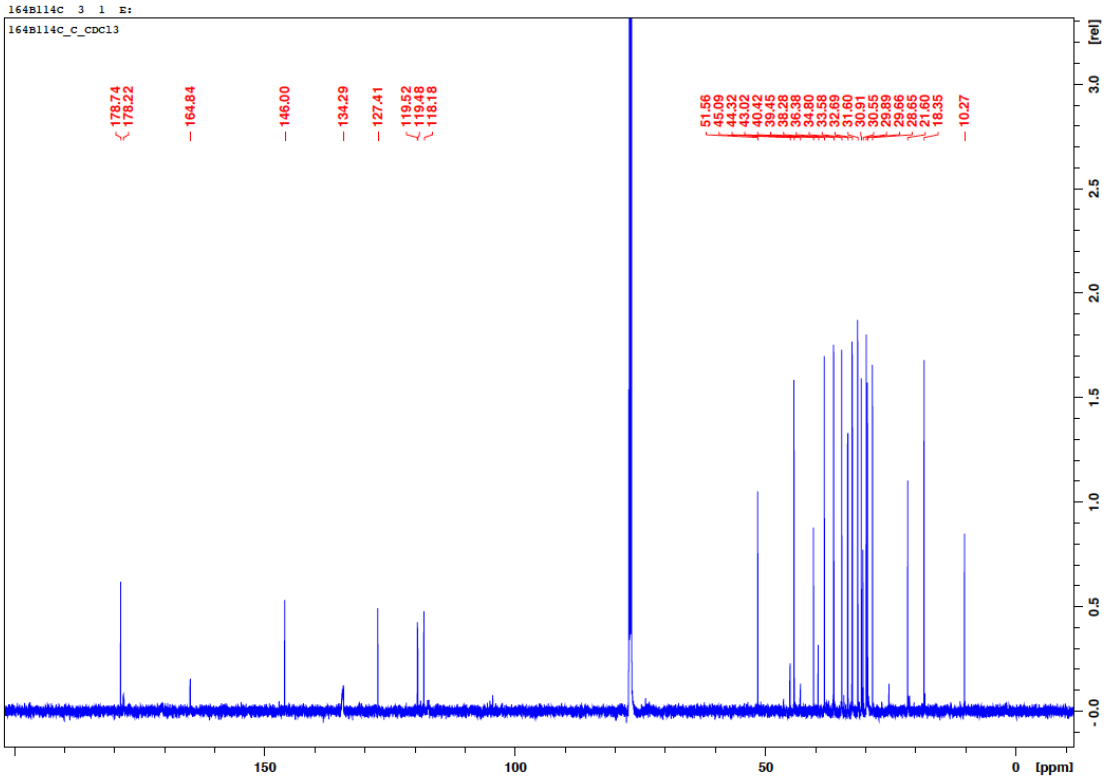

(S22) ^{13}C NMR spectrum (150 MHz) of pristimerin (**2**) in $CDCl_3$.

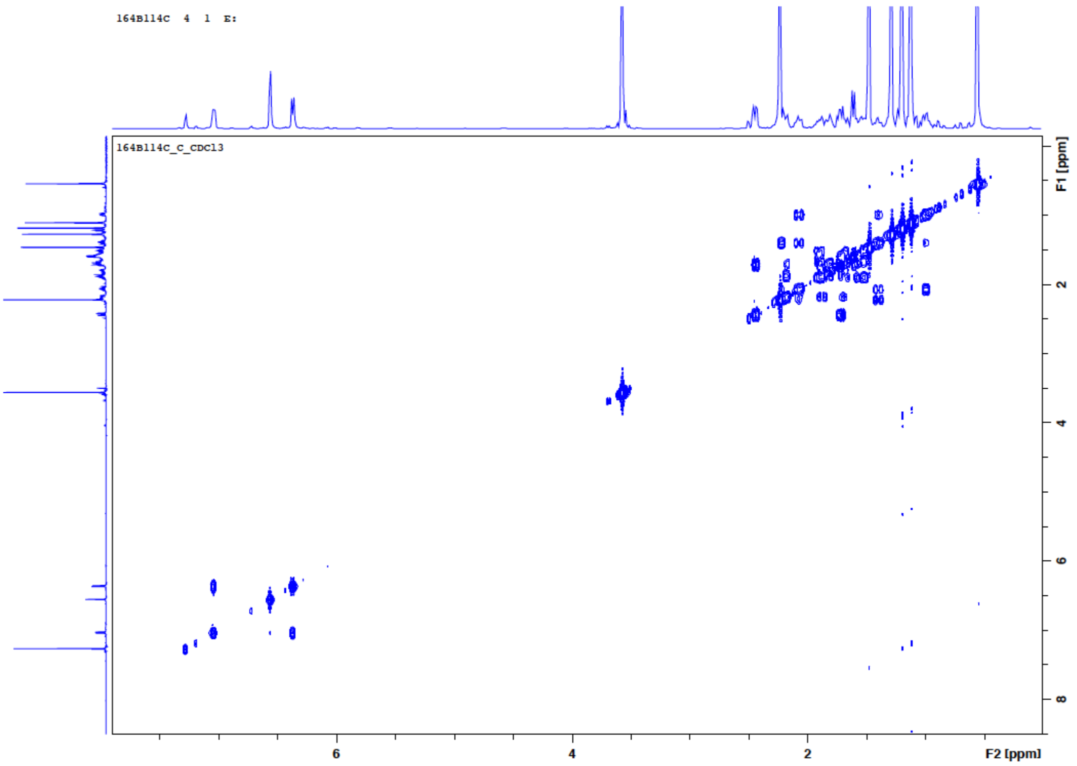

(S23) COSY spectrum of pristimerin (**2**) in $CDCl_3$.

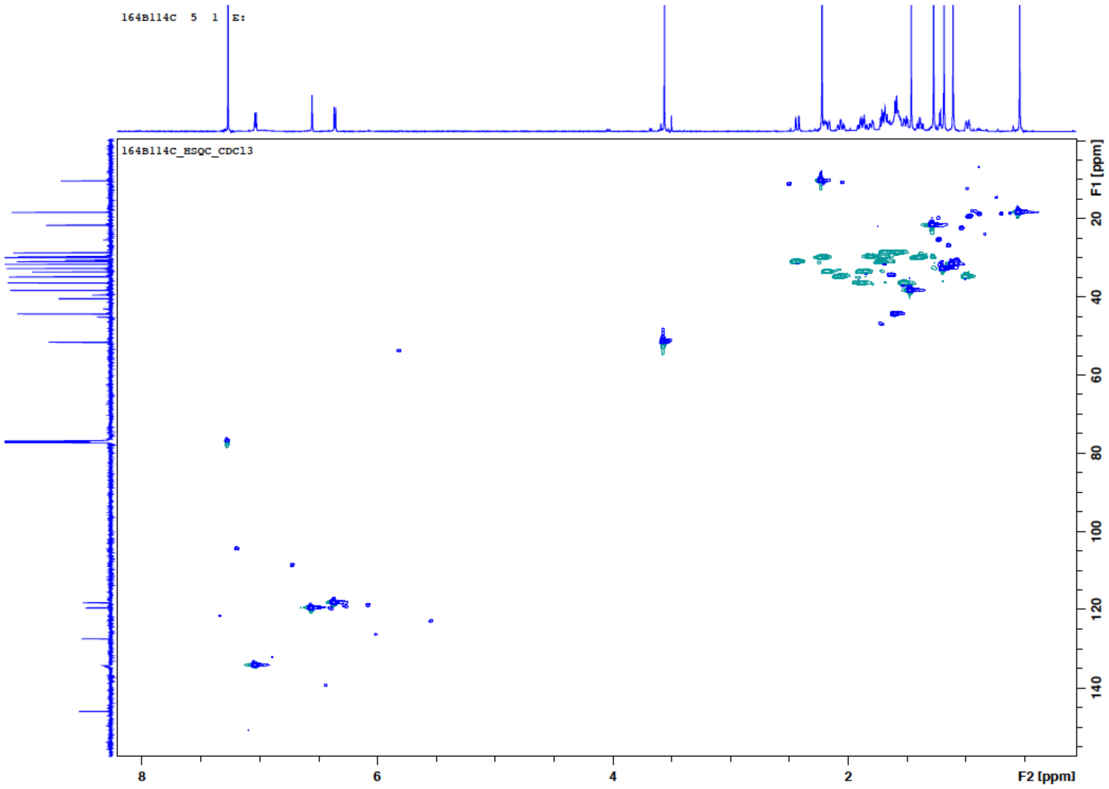

(S24) HSQC spectrum of pristimerin (**2**) in $CDCl_3$.

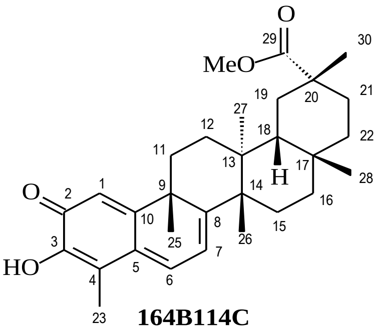

164B114C

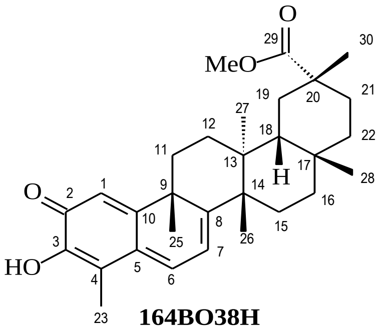

164BO38H

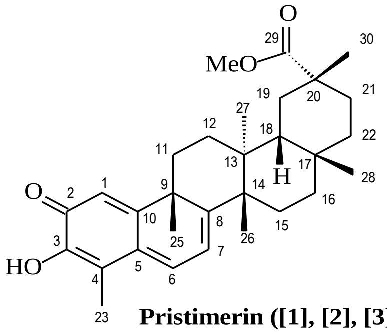

Pristimerin ([1], [2], [3])

	HSQC		**Pristimerin** [1]		**Pristimerin** [2]		**F164BO38H**		**[3],** CDCl3
	C	H	C	H	C	H	C		C
**C**									
2	178.76	-	178.4	-	178.4	-	178.91	-	188.3
3	146.02	-	146.1	-	146.1	-	145.81	-	147.8
4	117	-	117.0	-	117.0	-	121.12	-	108.7
5	127.13	-	127.5	-	127.5	-	127.20	-	125.8
8	171.10	-	169.9	-	169.9	-	182.11	-	151.1
9	43.02	-	42.9	-	42.9	-	45.56	-	40.5
10	164.93	-	164.7	-	164.7	-	166.66	-	172.3
13	39.45	-	39.4	-	39.4	-	40.47	-	39.0
14	45.10	-	45.0	-	45.0	-	46.44	-	44.6
17	30.55	-	30.6	-	30.6	-	29.92	-	30.4
20	40.42	-	40.4	-	40.4	-	40.39	-	40.1
29	178.27	-	178.7	-		-	178.81	-	179.7
**CH**									
1	119.53	6.54 (s)	119.6	6.53 (d, 1.3)	119.6	6.53 (d, 1.3)	119.78	6.83 (s)	125.2
6	134	7.02 (d, 7.2)	133.9	7.02 (dd,7.0, 1.0)	133.9	7.02 (dd,7.0, 1.3)	142.30	7.32 (d, 7.2)	141.1
7	118.25	6.35 (d, 7.2)	118.1	6.35 (d, 7.0)	118.1	6.35 (d, 7.0)	117.70	6.52 (d, 7.2)	122.4
18	44.32	1.59 (d, 8.2)	44.4	1.58 (d, 8.0)	44.4		44.37	1.61 (m)	44.2
**CH2**									
11	33.58	2.16 (br dd, 13.9; 4.1)<br>1.87 (td, 13.9, 5.6)	33.6	2.16 (ddd, 13.0, 4.5, 3.0)<br>1.86 (td, 13.0, 5.0)	33.6		33.81	2.26 (m)<br>1.90 (m)	33.9
12	29.66	1.80 (dd, 13.9, 4.1)<br>1.68 (m)	29.7	1.80 (ddd, 13.0, 5.0, 2.0)<br>1.68 (td, 13.0, 4.5)	**35.15?!**		29.47	1,88 (m)<br>1.71 (m)	29.6
15	28.65	1.67 (td, 13.9, 5.6)<br>1.56 (m)	28.7	1.66 (td, 14.0, 5.5)<br>1.56 (td, 14.0, 6.0, 2.0)	28.7		28.68	1.70 (m)<br>1.60 (m)	28.4
16	36.39	1.89 (td, 14.4, 6.1)<br>1.51 (dd, 14.4, 5.2)	36.4	1.88 (td, 14.0, 6.0)<br>1.50 (ddd, 14.0, 5.5, 2.0)	36.4		36.25	1.88 (m)<br>1.53 (m)	36.3
19	30.91	2.43 (br d, 15.9)<br>1.71 (dd, 15.9, 8.2)	30.9	2.43 (br d, 15.0, 5.0?)<br>1.69 (dd, 15.0, 8.0)	30.9		30.94	2.24 (dl, 15.9)<br>1.70 (m)	30.9
21	29.89	2.21 (m)<br>1.39 (td, 14.4, 4.6)	29.9	2.20 (ddd, 14.0, 4.5, 2.0)<br>1.38 (td, 14.0, 4.0)	29.9		29.86	2.21 (dl, 13.0)<br>1,38 (dt)	29.8
22	34.81	2.06 (td, 14.4, 3.6)<br>0.98 (br d)	34.8	2.05 (td, 14.0, 4.5)<br>0.98 (ddd, 14.0, 4.0, 2.0)	34.8		34.77	2.04 (m)<br>0.99 (dl, 13.9)	34.8
**CH3**									

23	10.23	2.22 (s)	10.2	2.21 (s)	10.2	2.21 (s)	10.70	2.29 (s)	13.6
25	38.29	1.46 (s)	38.3	1.45 (s)	38.3	1.45 (s)	38.29	1.48 (s)	37.6
26	21.60	1.27 (s)	21.6	1.26 (s)	21.4	1.26 (s)	21.07	1.29 (s)	20.6
27	18.35	0.54 (s)	18.3	0.53 (s)	18.3	0.53 (s)	18.53	0.52 (s)	18.3
28	31.61	1.11 (s)	31.6	1.10 (s)	31.6	1.10 (s)	31.60	1.11 (s)	31.5
30	32.69	1.19 (s)	32.7	1.18 (s)	32.7	1.18 (s)	32.59	1.18 (s)	32.9
MeO	51.57	3.56 (s)	51.6	3.55 (s)			51.67	3.56 (s)	51.1

(S25) NMR Spectroscopic Data (^{1}H 600 MHz, ^{13}C 150 MHz) for pristimerin (**2**) in $CDCl_3$.

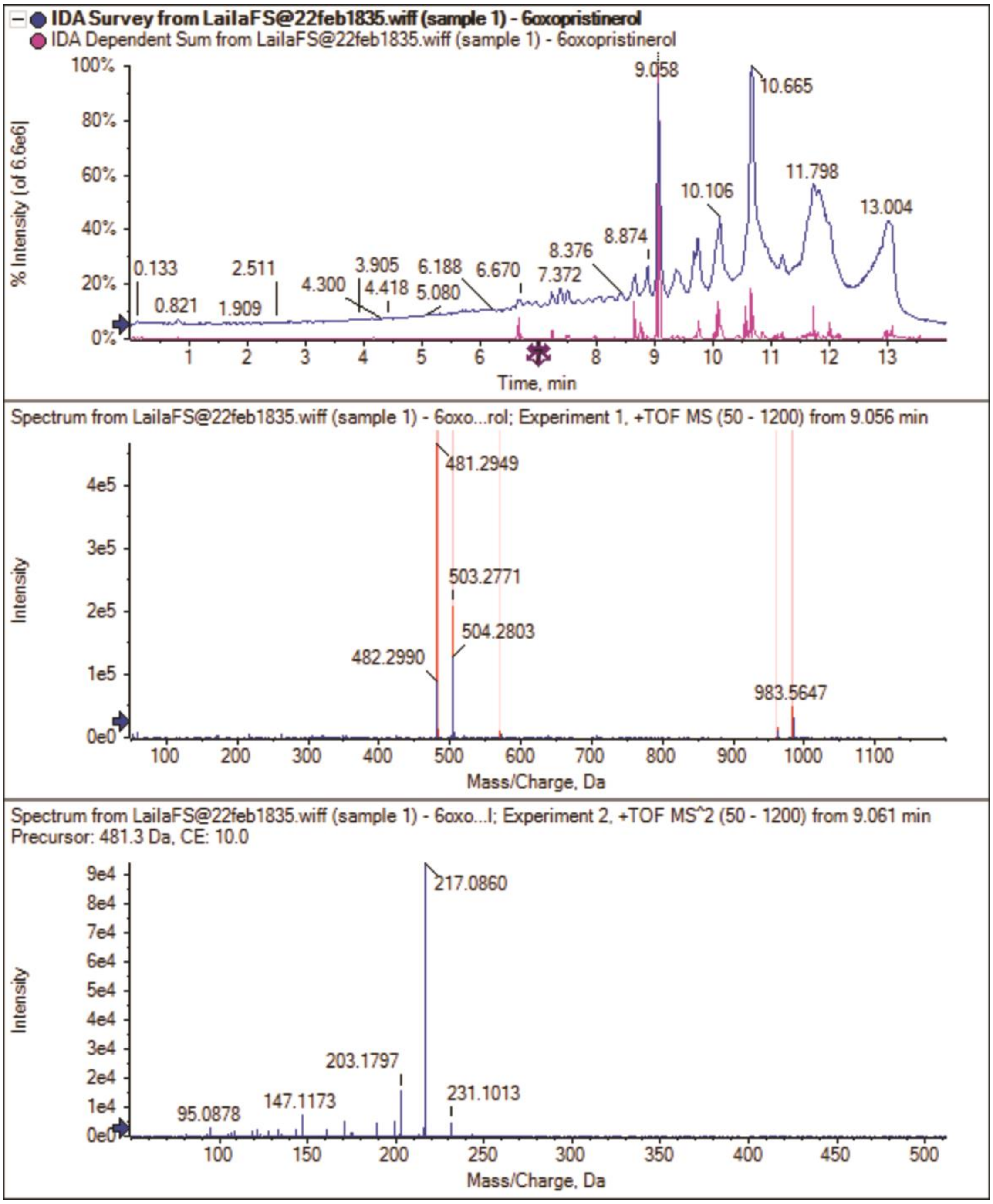

(S26) Chromatogram, MS1 and MS2 spectra of the LC-MS/MS analysis of 6-oxopristimerol (**3**).

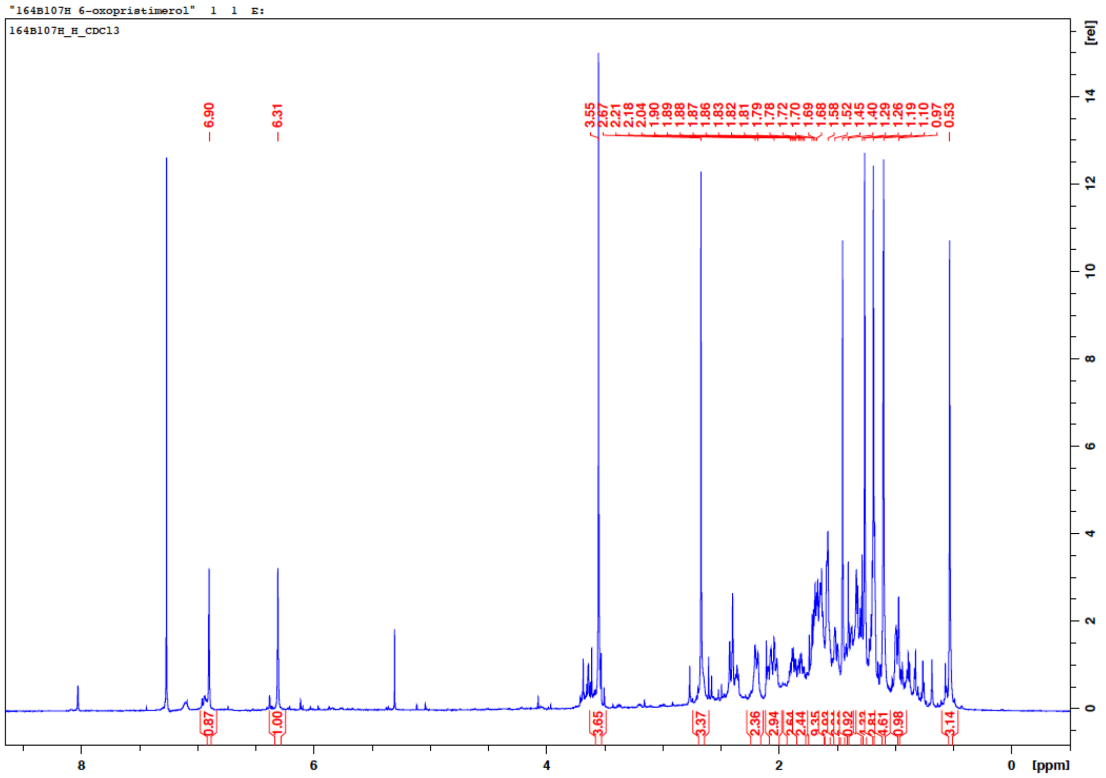

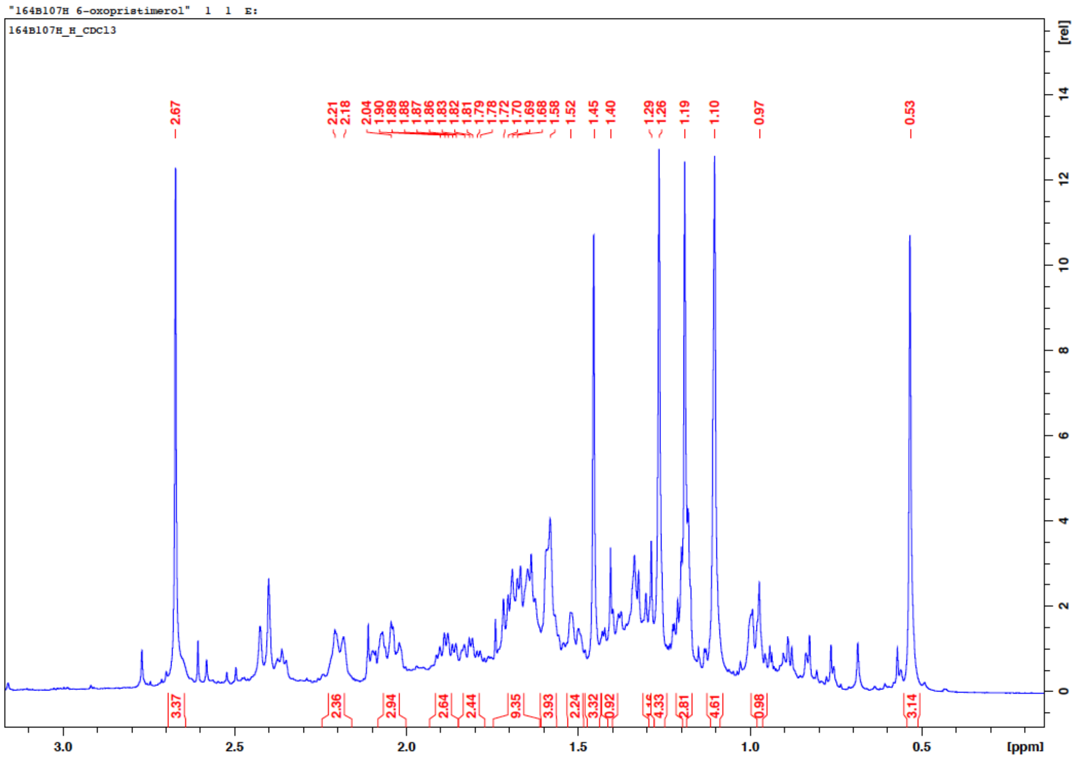

(S27) ^{1}H NMR spectrum (600 MHz) of 6-oxopristimerol (**3**) in $CDCl_3$.

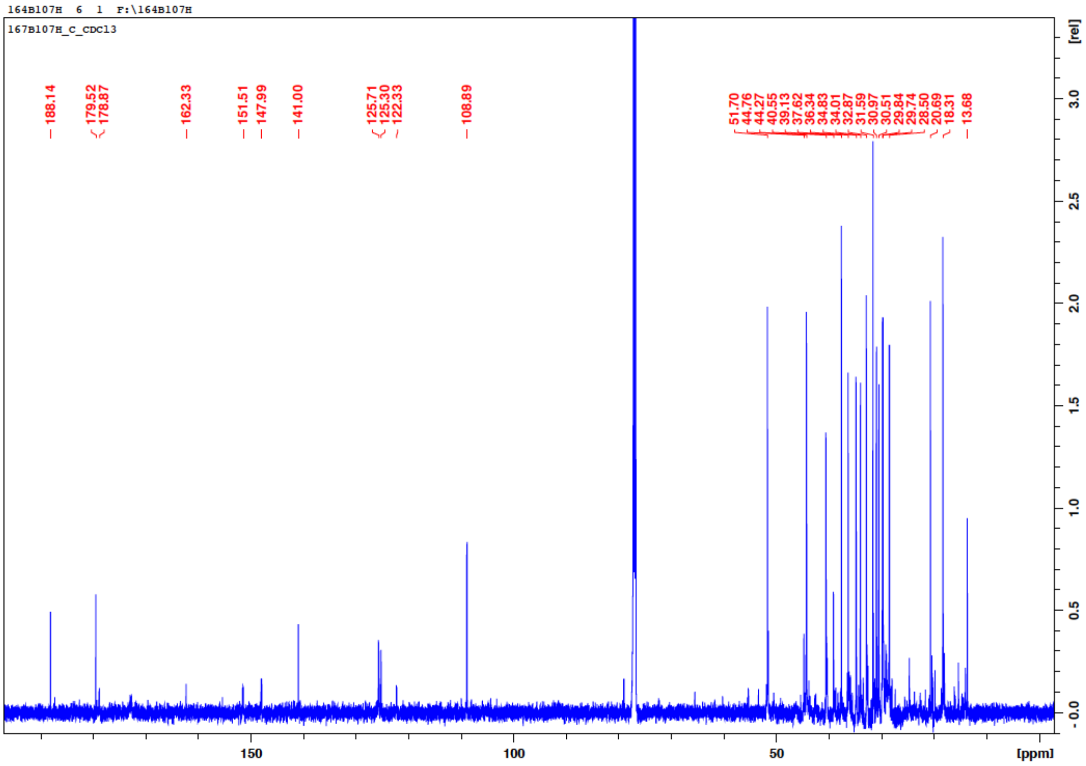

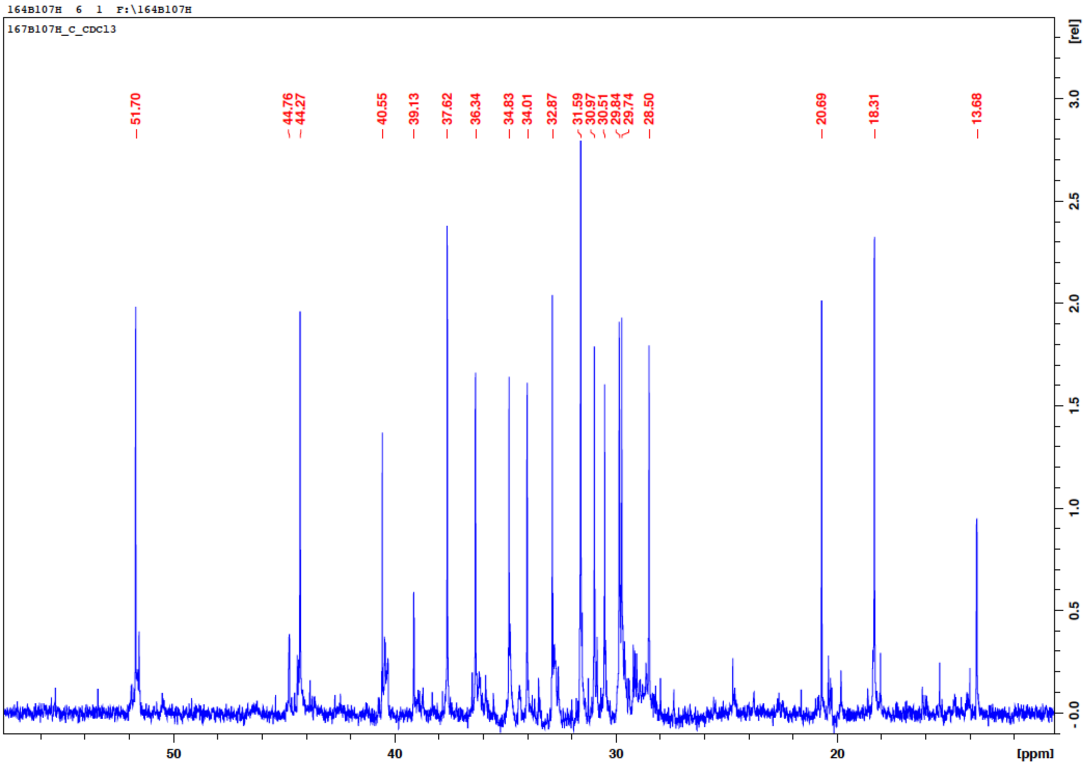

(S28) ^{13}C NMR spectrum (150 MHz) of 6-oxopristimerol (**3**) in $CDCl_3$.

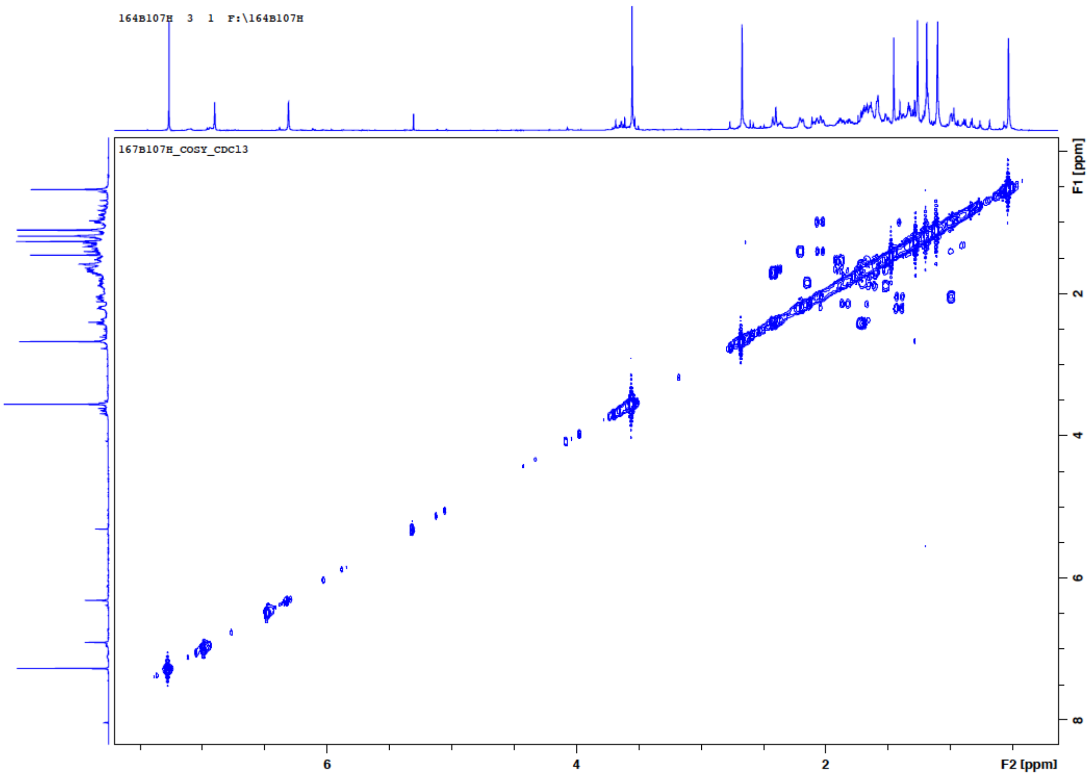

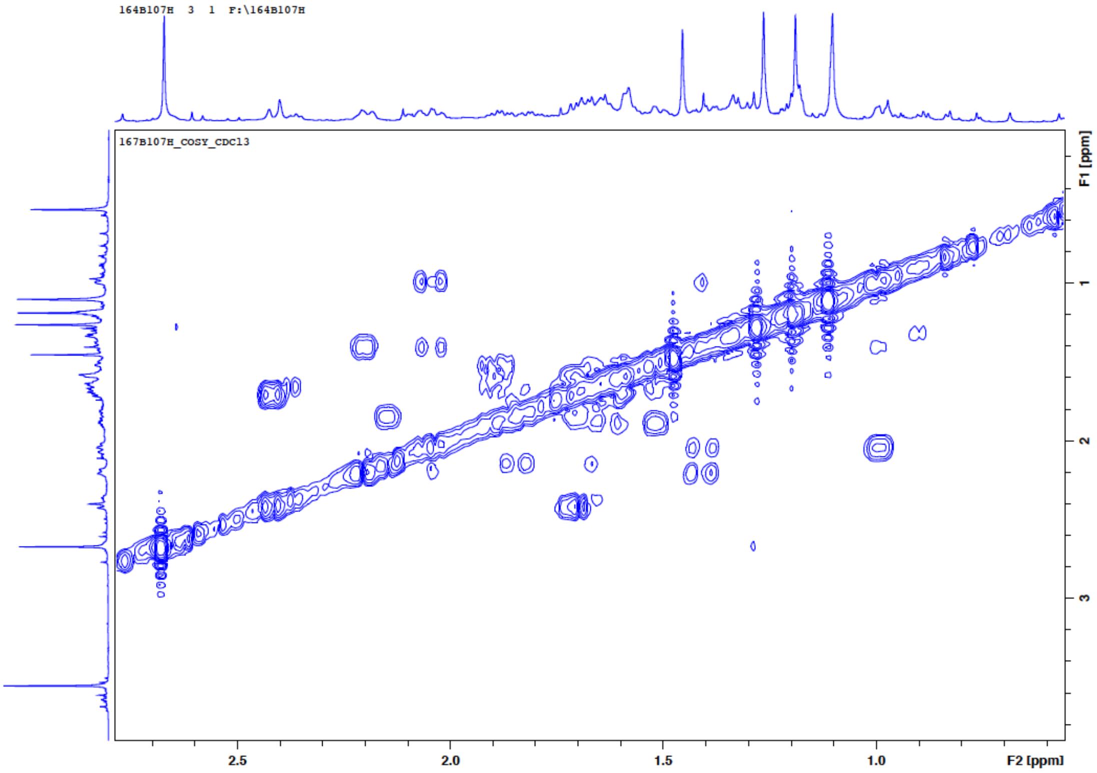

(S29) COSY spectrum of 6-oxopristimerol (**3**) in $CDCl_3$.

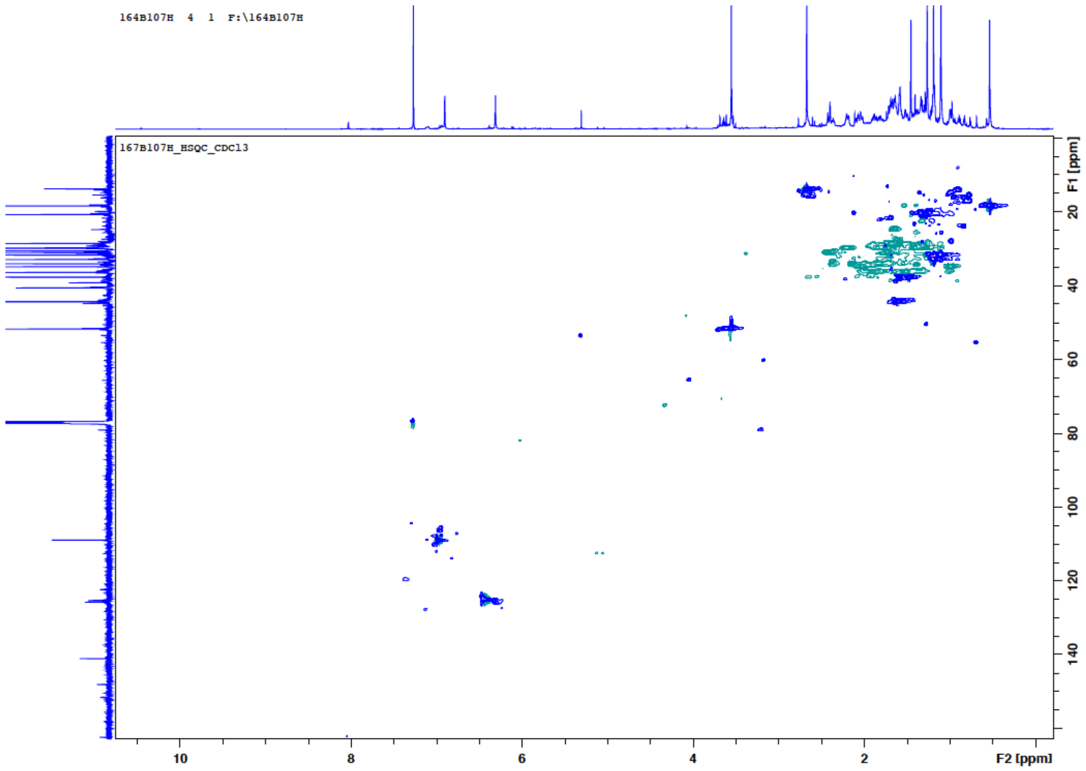

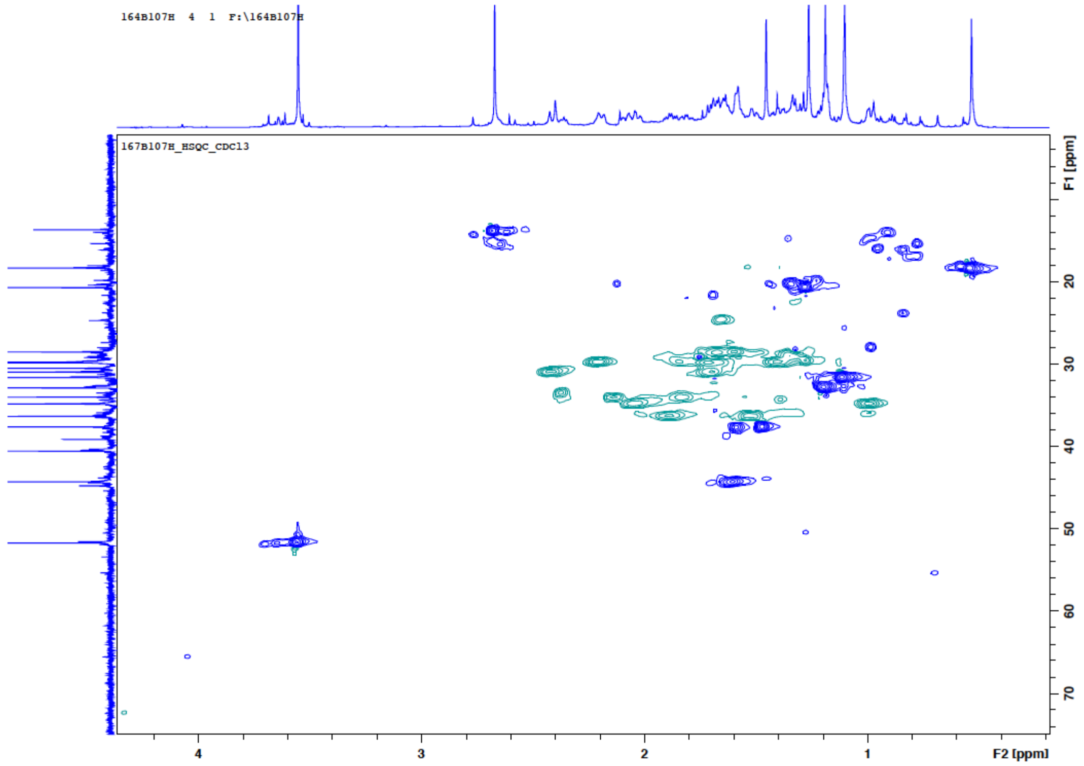

(S30) HSQC spectrum of 6-oxopristimerol (**3**) in $CDCl_3$.

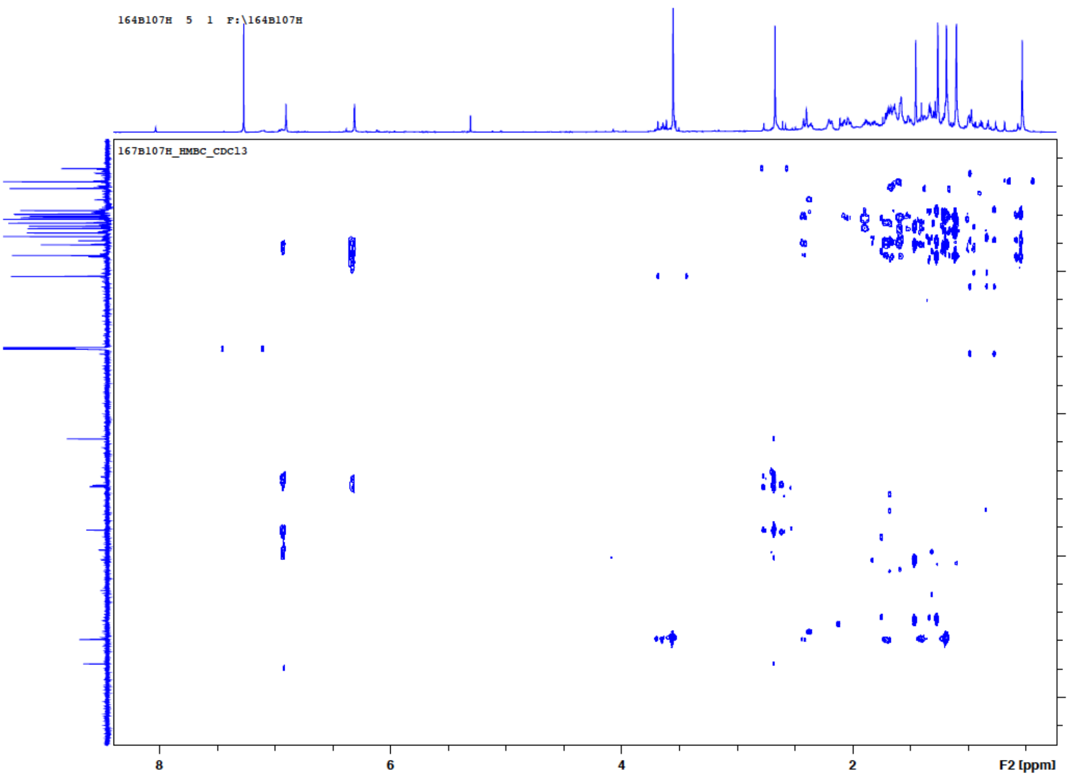

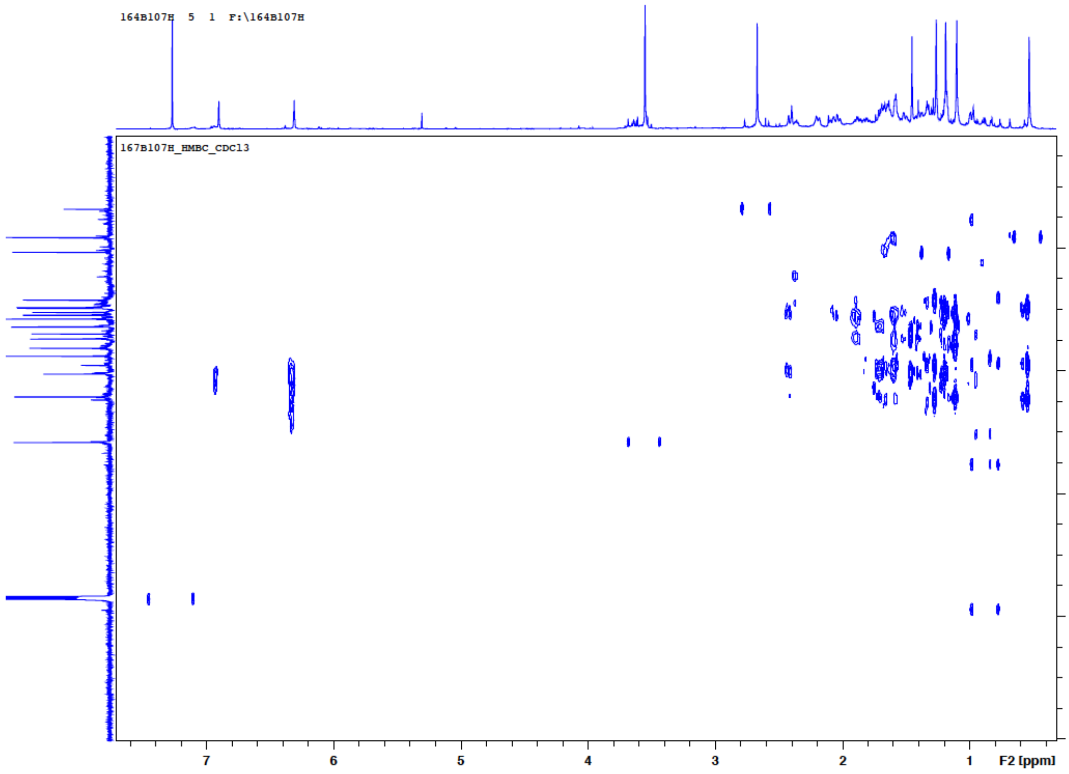

(S31) HMBC spectrum of 6-oxopristimerol (**3**) in $CDCl_3$.

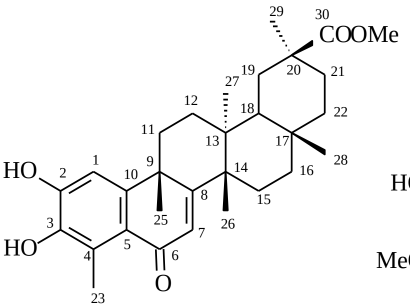

6-Oxopristimerol
J. Nat. Prod. **1994**, *57*(12), 1675

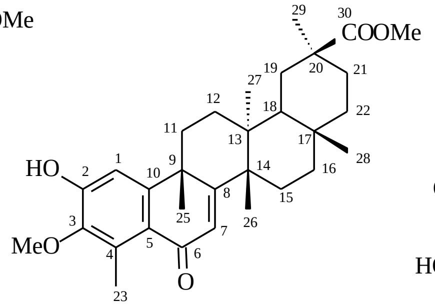

3-*O*-methyl-6-oxopristimerol
Chemistry & biodiversity **2011**, *8*, 2291

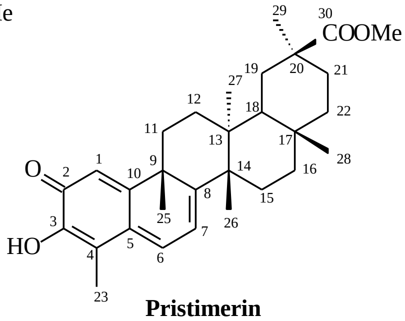

Pristimerin

	HSQC		HMBC		**6-Oxopristimerol,** em C_5D_5N		**3-O-methyl-6-Oxopristimerol,** $CDCl_3$	
	C	H	$^2J_{CH}$	$^3J_{CH}$	C	H	C	H
**C**								
2	147.99	-	H-1		144.04	-	144.2	-
3	141.00	-		H-1; 3H-23	126.80	-	132.9	-
4	125.30	-	3H-23		122.67	-	132.0	-
5	122.33	-		H-1; H-7; 3H-23	151.38	-	154.9	-
6	188.44	-			187.32	-	187.2	-
8	172.99	-		3H-25; 3H-26	150.67	-	151.9	-
9	40.55	-	3H-25		40.58	-	40.4	-
10	151.51	-	H-1	3H-25	170.77	-	171.2	-
13	39.12	-	3H-27	3H-26	39.30	-	38.9	-
14	44.75	-	3H-26	3H-27	44.61	-	44.7	-
17	30.96	-	3H-28		30.64	-	30.8	-
20	40.55	-	3H-30		40.14	-	40.3	-
29	179.52	-		3H-30; MeO-29	178.66	-	178.8	-
**CH**								
1	108.89	6.98 (s)			109.99	7.25 (s)	109.1	6.95 (s)
6	-	-	-	-	-	-	-	-
7	125.12	6.46 (s)			126.88	6.46 (s)	126.1	6.14 (s)
18	44.27	1.60		3H-27; 3H-28	44.38	1.58	44.3	
**CH_2**								
11	34.00	2.14, 1.83		3H-25	34.59		34.0	2.21, 1.94
12	29.73	1.84, 1.27		3H-27	30.21		29.8	1.72, 1.66
15	28.50	1.70, 1.59		3H-26	28.86		28.5	1.80, 1.40
16	36.34	1.88, 1.52		3H-28	36.62		36.4	1.80, 1.50
19	30.51	2.14, 1.69		3H-30	31.08		30.5	2.91 (d, 15.9), 1.66

21	29.89	2.20, 1.40		3H-30	30.11		29.7	2.19, 1.37
22	34.82	2.04, 0.98		3H-28	35.13		34.8	2.04, 0.97
**CH_3**								
23	13.68	2.67 (s)			14.82	3.28 (s)	14.7	2.66 (s)
25	37.63	1.46 (s)			37.76	1.53 (s)	37.6	1.55 (s)
26	20.69	1.27 (s)			20.87	1.16 (s)	20.8	1.29 (s)
27	18.31	0.53 (s)			18.51	0.63 (s)	18.3	0.57 (s)
28	31.58	1.10 (s)			31.55	0.99 (s)	31.6	1.09 (s)
30	32.87	1.18 (s)			32.59	1.15 (s)	32.7	1.17 (s)
MeO-29	51.70	3.55 (s)			51.47	3.58 (s)	51.5	3.57 (s)
MeO-3	-	-	-	-	-	-	61.1	3.79 (s)

(S32) NMR Spectroscopic Data (^{1}H 600 MHz, ^{13}C 150 MHz) for 6-oxopristimerol (**3**) in $CDCl_3$.

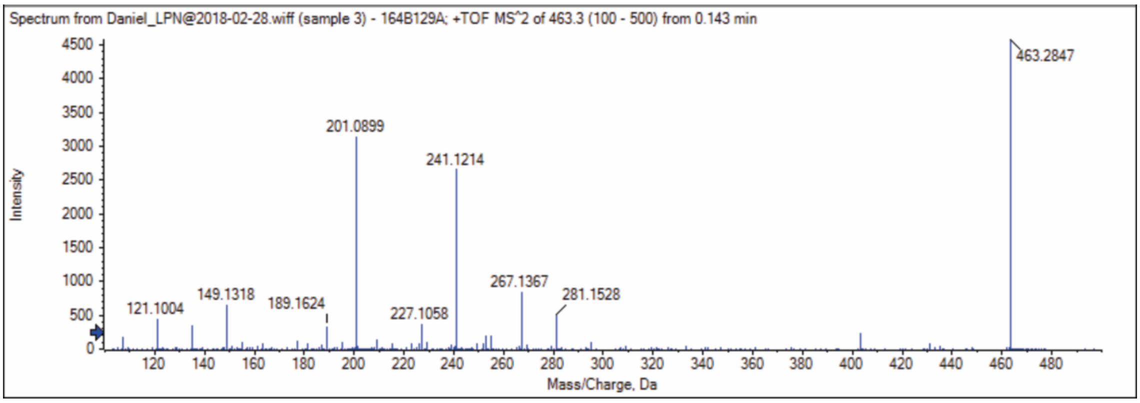

(S33) MS2 Mass spec of vitideasin (**4**).

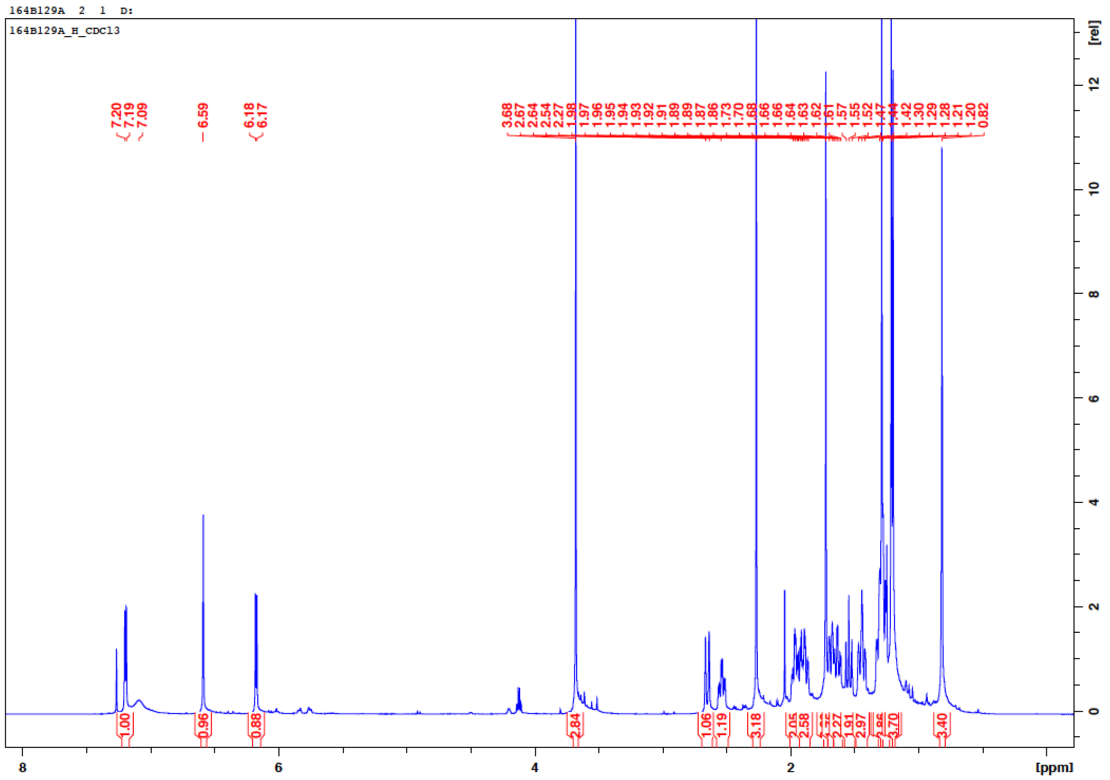
164B129A 2 1 D:
164B129A_H_CDCl3
[rel]
[ppm]

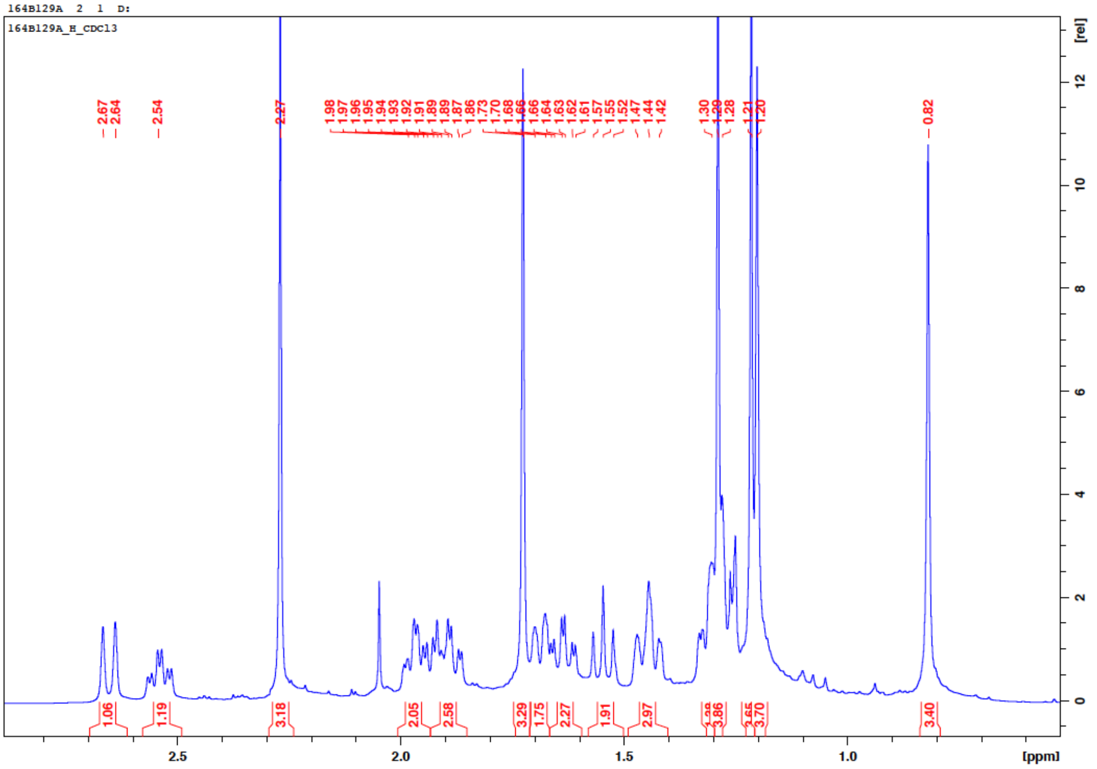
164B129A 2 1 D:
164B129A_H_CDCl3
[rel]
[ppm]

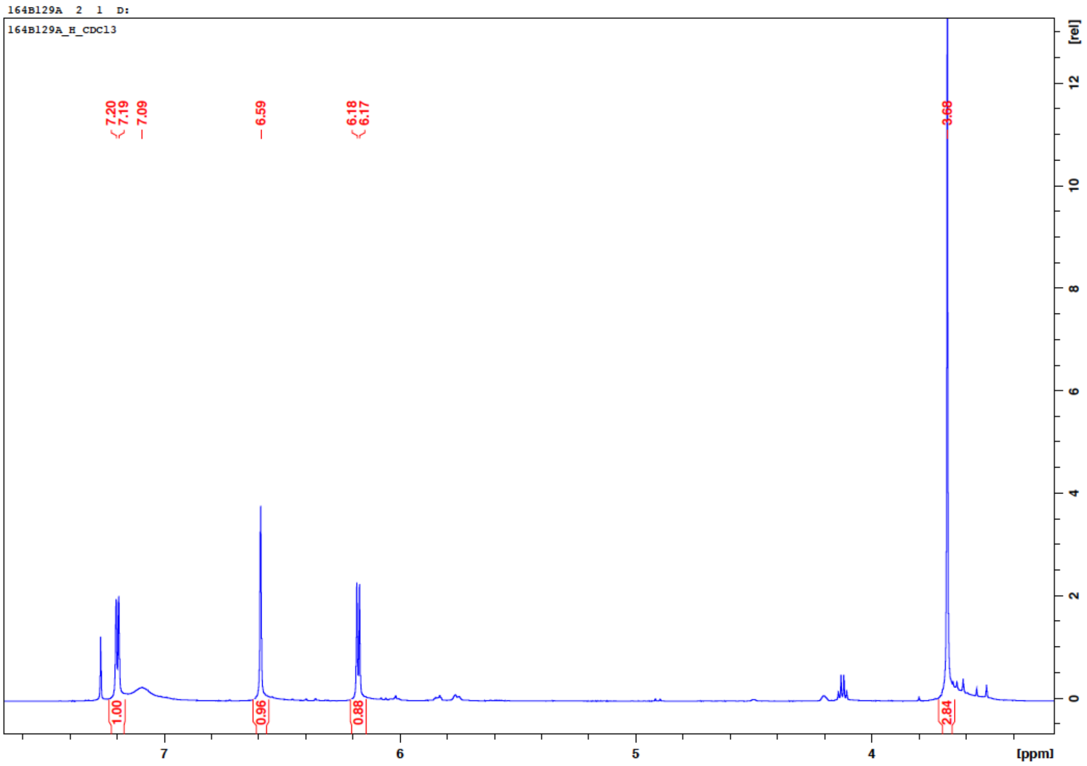

(S34) ^{1}H NMR spectrum (600 MHz) of vitideasin (**4**) in $CDCl_3$.

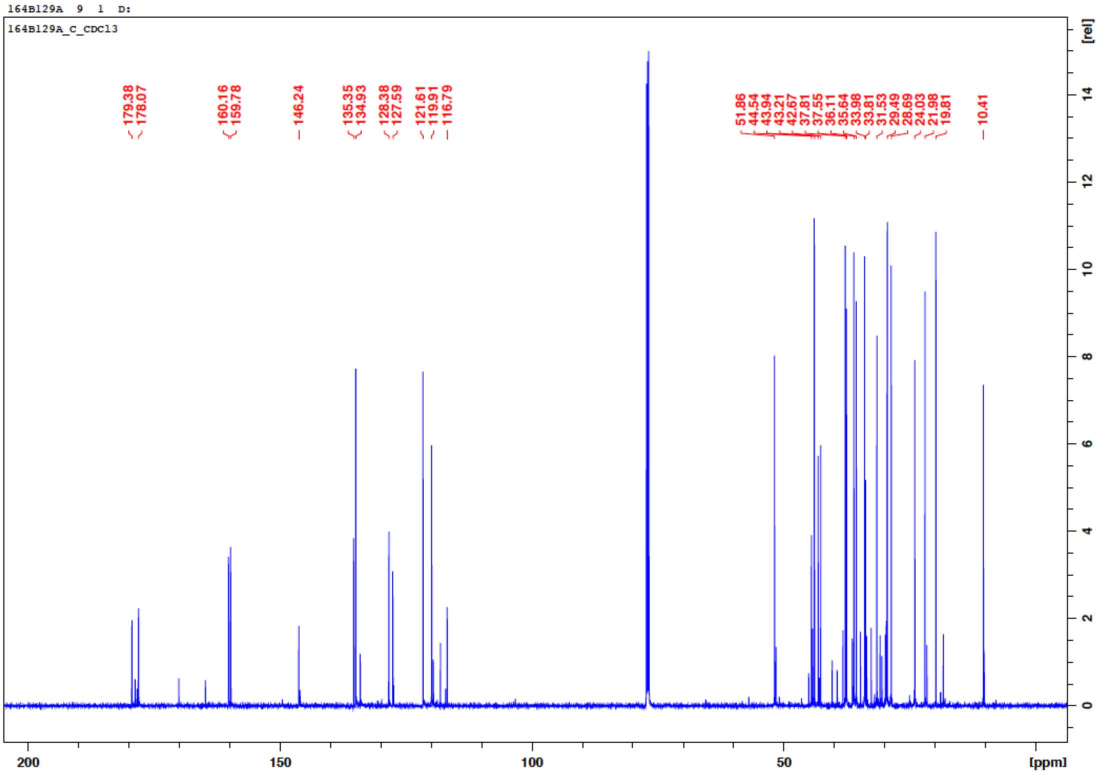

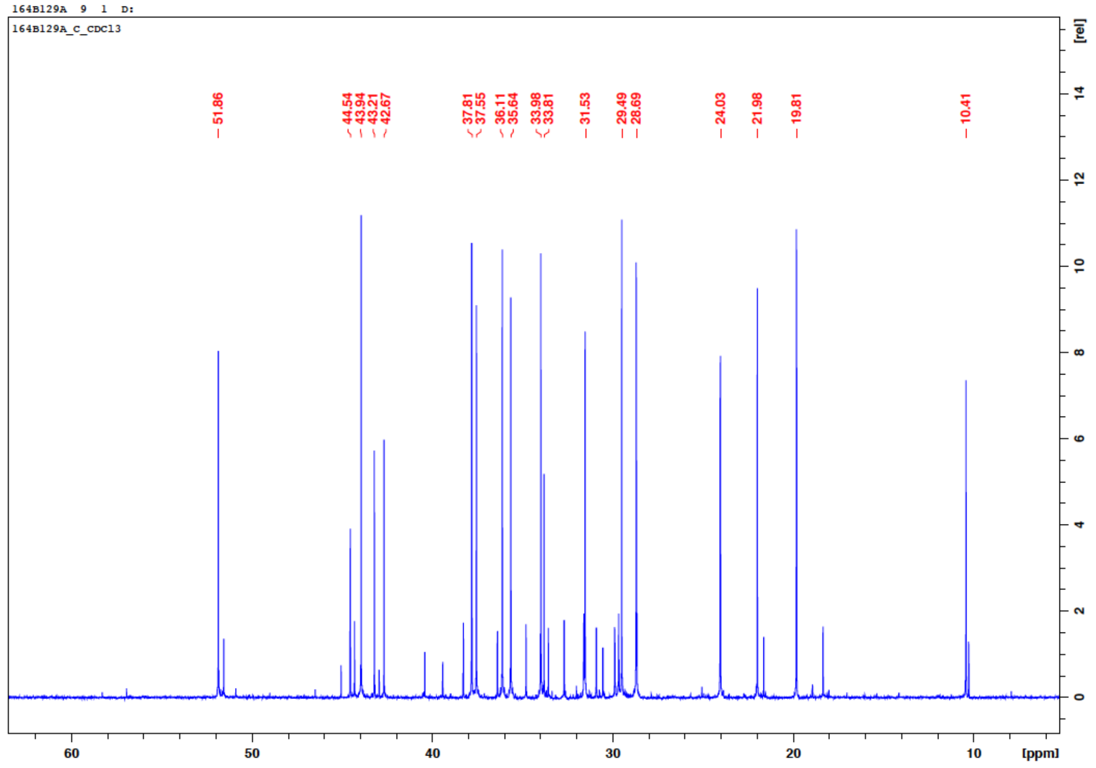

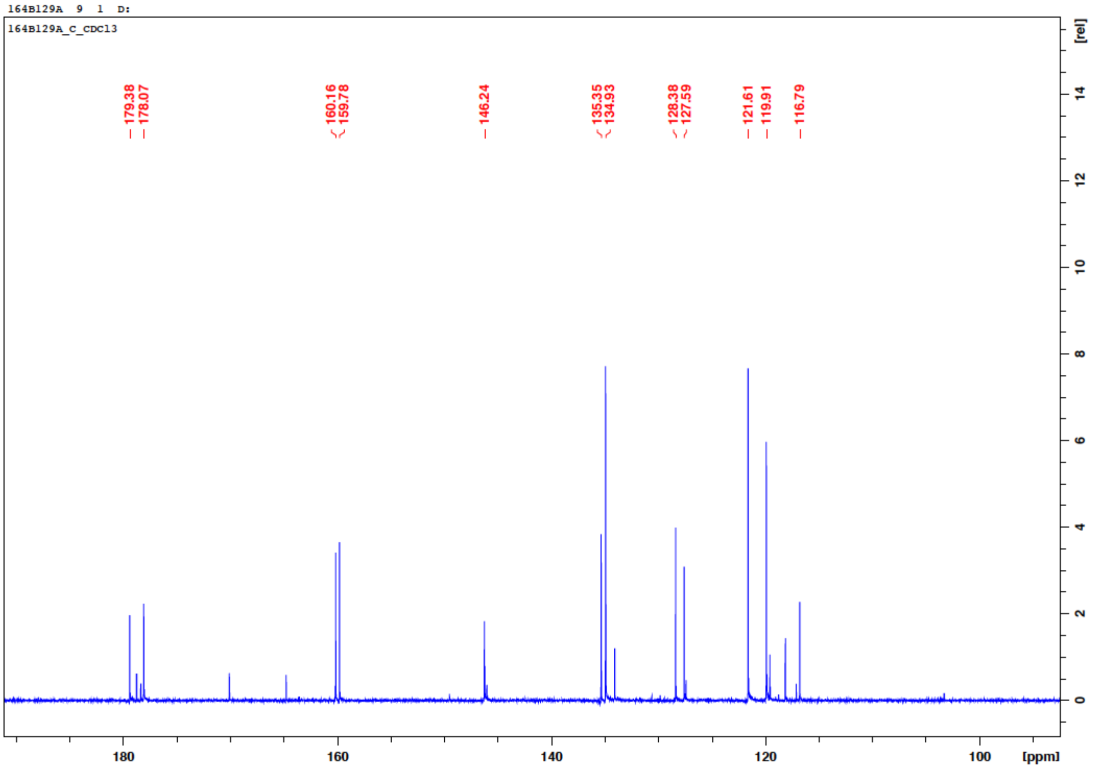

(S35) ^{13}C NMR spectrum (150 MHz) of vitideasin (**4**) in $CDCl_3$.

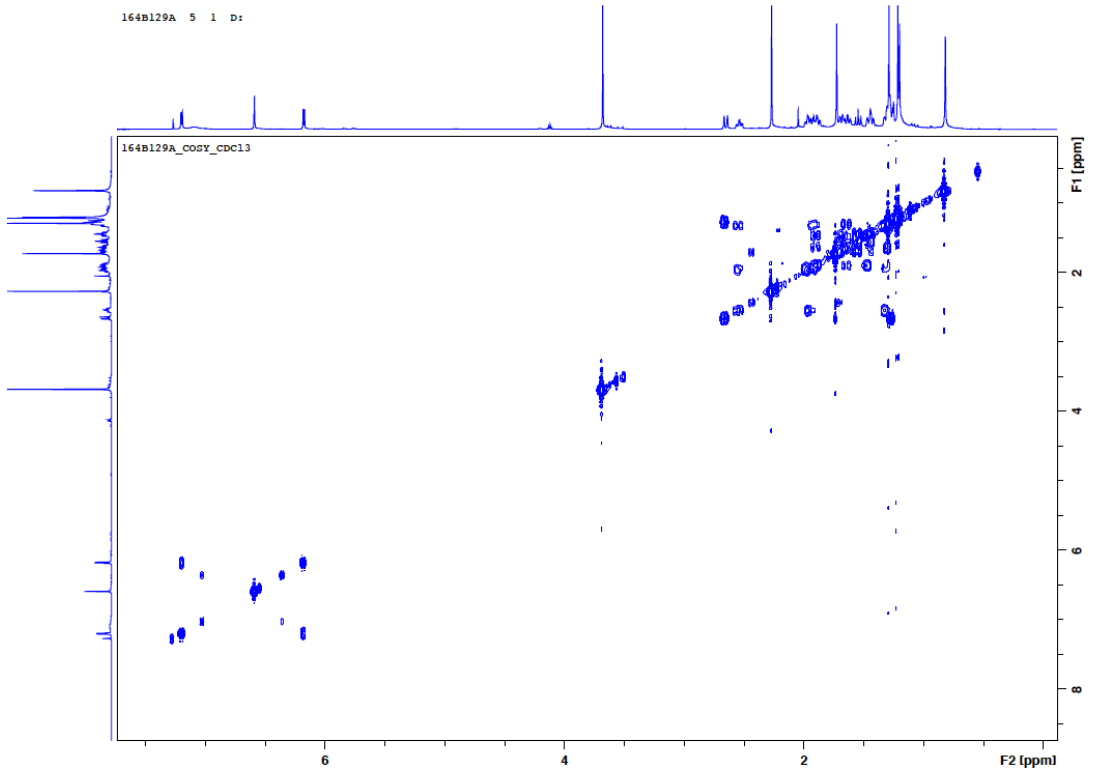

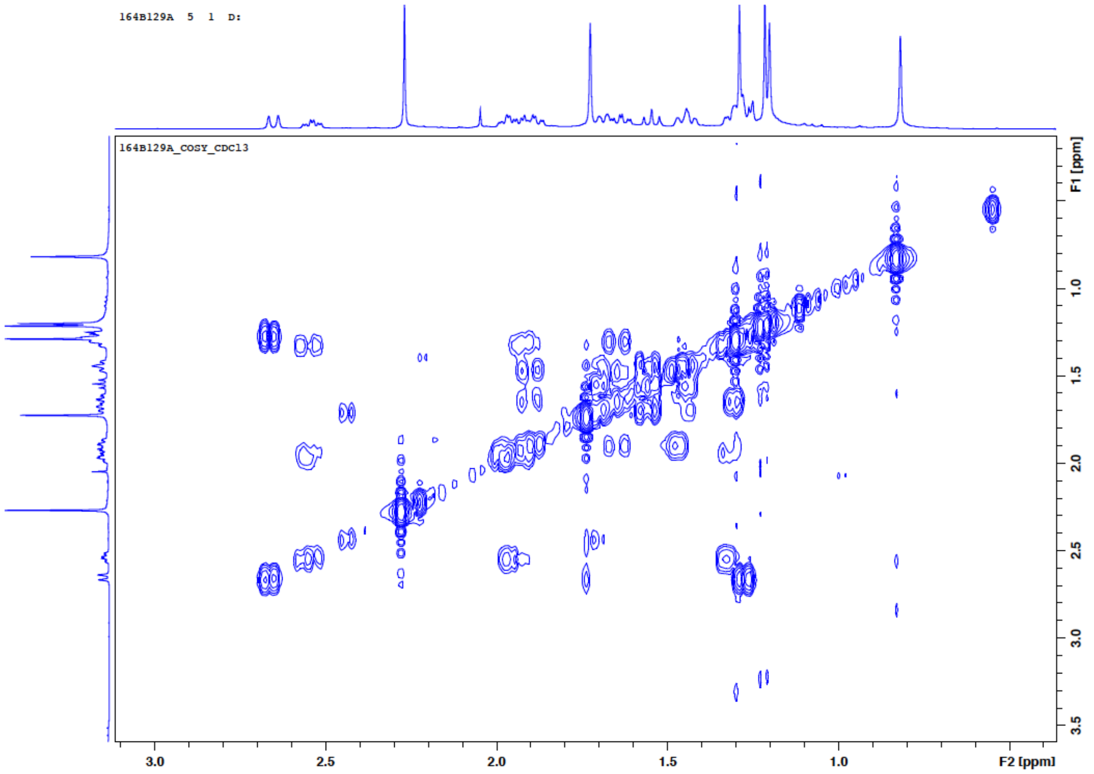

(S36) COSY spectrum of vitideasin (**4**) in $CDCl_3$.

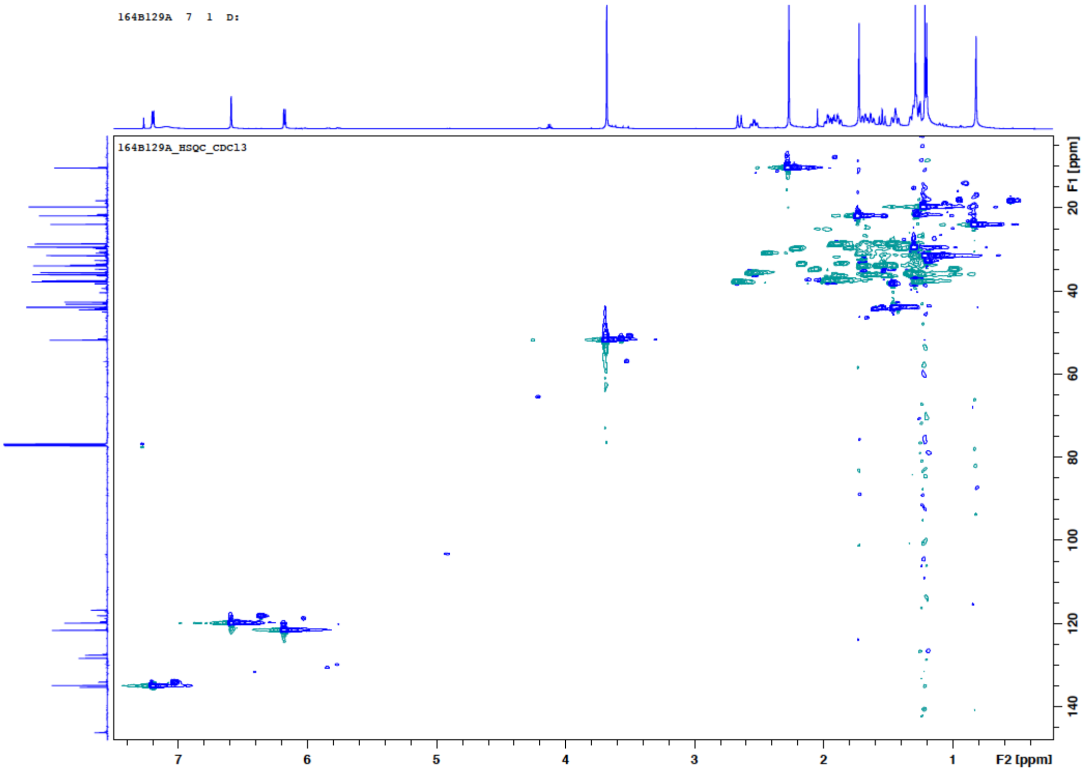

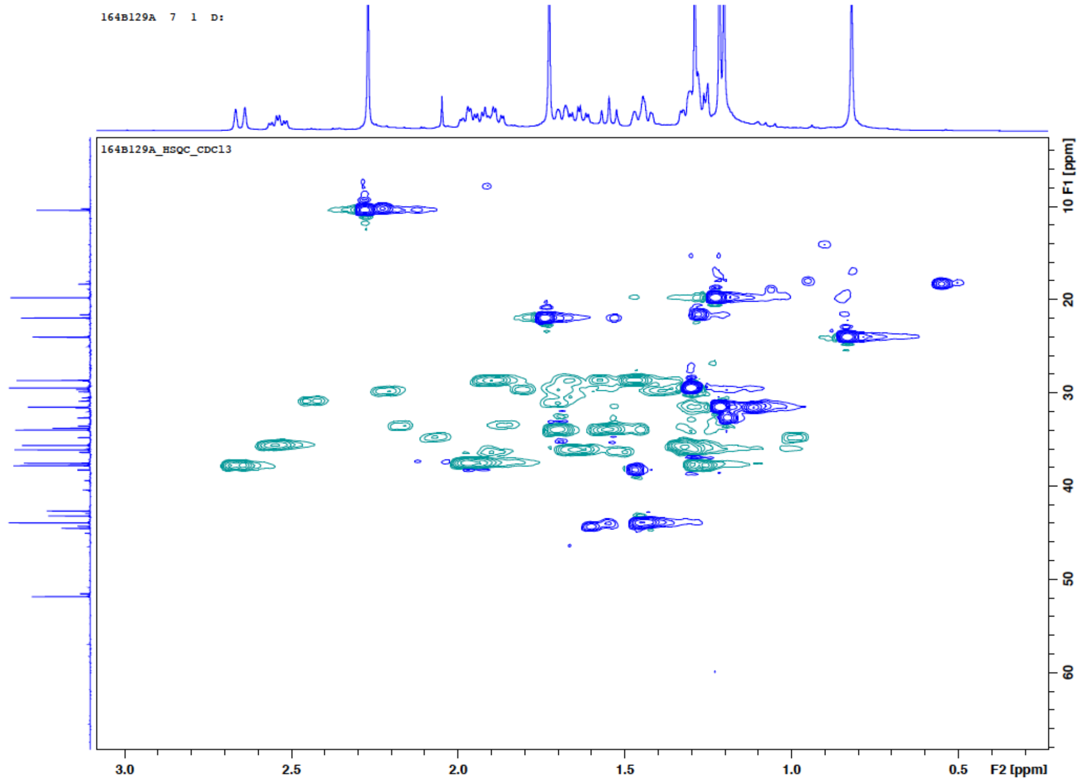

(S37) HSQC spectrum of vitideasin (**4**) in $CDCl_3$.

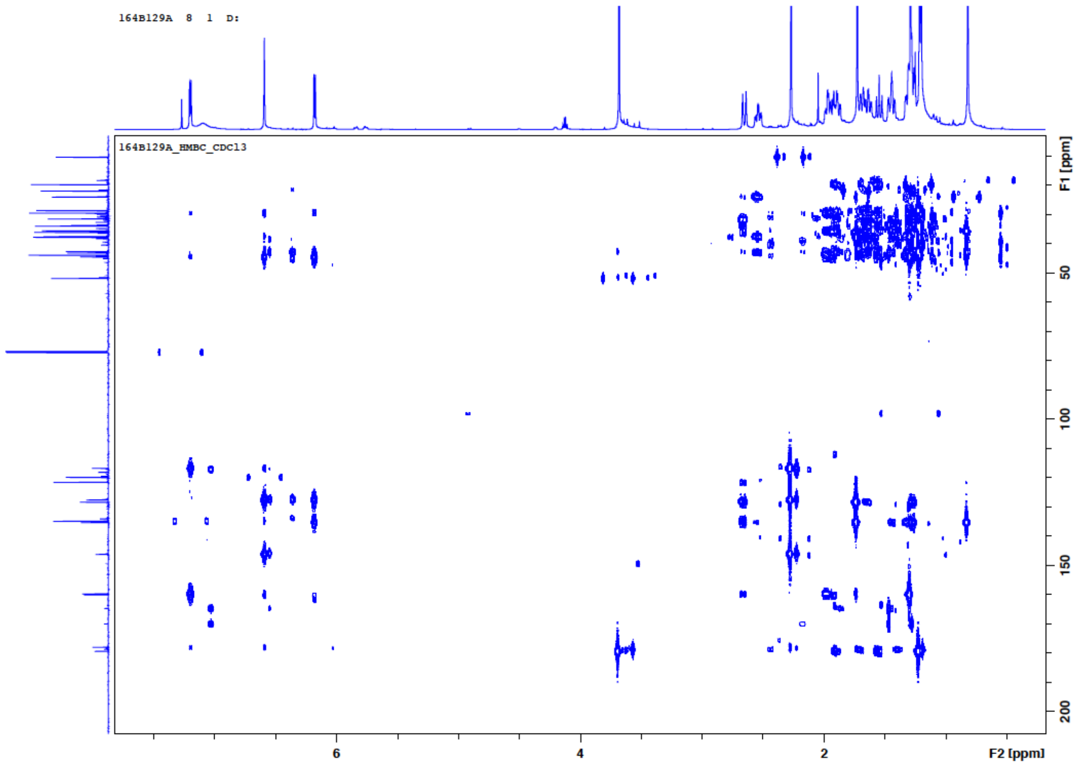

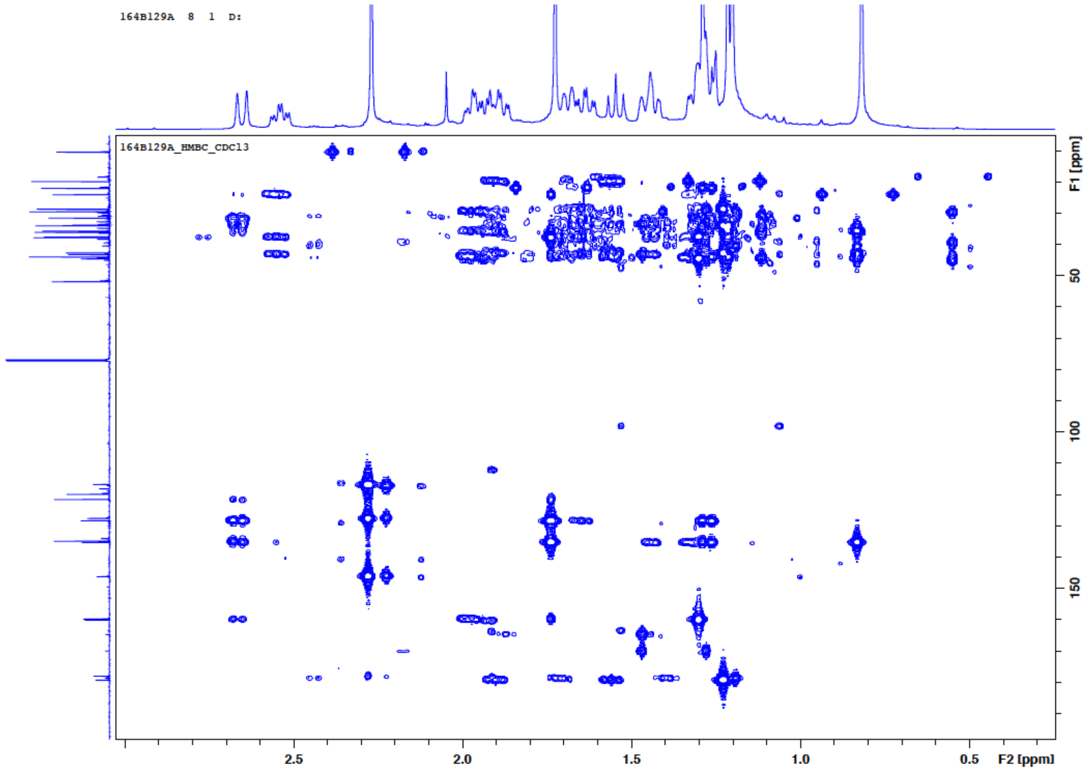

(S38) HMBC spectrum of vitideasin (**4**) in $CDCl_3$.

	Vitideasin (Compound 4)	
	C	H
**C**		
2	178.0	-
3	146.2	-
4	116.7	-
5	127.5	-
8	159.7	-
9	44.5	-
10	160.13	-
13	43.1	-
14	135.3	-
15	128.3	-
17	33.7	-
20	42.6	-
29	179.3	-
**CH**		
1	119.9	6.59 (s)
6	134.9	7.19 (d, 7.0 Hz)
7	121.6	6.17 (d, 7.0 Hz)
11	37.5	1.93 - 2.00 (m)
18	43.9	1.40 - 1.48 (m)
**CH_2**		
12	35.6	2.54 (ddd); 1.3 (overlapped)
16	37.8	2.65 (d); 1.28 (overlapped)
19	33.9	1.54 (t); 1.68-1.71 (m)
21	28.6	1.46 - 1.48 (m); 1.85 - 1.93 (m)
22	36.1	1.64 (m)
**CH_3**		
23	10.3	2.2 (s)
25	29.4	1.3 (m)
26	21.9	1.73 (s)
27	24.0	0.82 (s)
28	31.5	1.20 (s)
30	19.8	1.21 (s)
MeO	51.8	3.68 (s)
OH		7.09 (bs)

(S39) NMR Spectroscopic Data (^{1}H 600 MHz, ^{13}C 150 MHz) for vitideasin (**4**) in $CDCl_3$.

	S. crassifolia NSC:N192803 Endpt:GI50 ExpId:AVGDATA hiConc:2.0	*S. elliptica* NSC:N192805 Endpt:GI50 ExpId:AVGDATA hiConc:2.0
S. crassifolia NSC:N192803 Endpt:GI50 ExpId:AVGDATA hiConc:2.0	1.0	0.749
S. elliptica NSC:N192805 Endpt:GI50 ExpId:AVGDATA hiConc:2.0	0.749	1.0

	S. crassifolia NSC:N192803 Endpt:TGI ExpId:AVGDATA hiConc:2.0	*S. elliptica* NSC:N192805 Endpt:TGI ExpId:AVGDATA hiConc:2.0
S. crassifolia NSC:N192803 Endpt:TGI ExpId:AVGDATA hiConc:2.0	1.0	0.807
S. elliptica NSC:N192805 Endpt:TGI ExpId:AVGDATA hiConc:2.0	0.807	1.0

	S. crassifolia NSC:N192803 Endpt:LC50 ExpId:AVGDATA hiConc:2.0	*S. elliptica* NSC:N192805 Endpt:LC50 ExpId:AVGDATA hiConc:2.0
S. crassifolia NSC:N192803 Endpt:LC50 ExpId:AVGDATA hiConc:2.0	1.0	0.765
S. elliptica NSC:N192805 Endpt:LC50 ExpId:AVGDATA hiConc:2.0	0.765	1.0

(S40) COMPARE between *S. crassifolia* and *S. elliptica* extracts.

	PRISTIMERIN NSC:S791208 Endpt:GI50 ExpId:AVGDATA hiConc:-4.0	11B-HYDROXY-PRISTAMERIN NSC:S797253 Endpt:GI50 ExpId:AVGDATA hiConc:-4.1	*S. elliptica* NSC:N192805 Endpt:GI50 ExpId:AVGDA TA hiConc:2.0
PRISTIMERIN NSC:S791208 Endpt:GI50 ExpId:AVGDATA hiConc:-4.0	1.0	0.518	0.692
11B-HYDROXY-PRISTAMERIN NSC:S797253 Endpt:GI50 ExpId:AVGDATA hiConc:-4.1	0.518	1.0	0.642
S. elliptica NSC:N192805 Endpt:GI50 ExpId:AVGDATA hiConc:2.0	0.692	0.642	1.0

	PRISTIMERIN NSC:S791208 Endpt:TGI ExpId:AVGDATA hiConc:-4.0	11B-HYDROXY-PRISTAMERIN NSC:S797253 Endpt:TGI ExpId:AVGDATA hiConc:-4.1	*S. elliptica* NSC:N192805 Endpt:TGI ExpId:AVGDA TA hiConc:2.0
PRISTIMERIN NSC:S791208 Endpt:TGI ExpId:AVGDATA hiConc:-4.0	1.0	0.557	0.813
11B-HYDROXY-PRISTAMERIN NSC:S797253 Endpt:TGI ExpId:AVGDATA hiConc:-4.1	0.557	1.0	0.578
S. elliptica NSC:N192805 Endpt:TGI ExpId:AVGDATA hiConc:2.0	0.813	0.578	1.0

	PRISTIMERIN NSC:S791208 Endpt:TGI ExpId:AVGDATA hiConc:-4.0	11B-HYDROXY-PRISTAMERIN NSC:S797253 Endpt:TGI ExpId:AVGDATA hiConc:-4.1	*S. elliptica* NSC:N192805 Endpt:TGI ExpId:AVGDA TA hiConc:2.0
PRISTIMERIN NSC:S791208 Endpt:LC50 ExpId:AVGDATA hiConc:-4.0	1.0	0.745	0.86
11B-HYDROXY-PRISTAMERIN NSC:S797253 Endpt:LC50 ExpId:AVGDATA hiConc:-4.1	0.745	1.0	0.749
S. elliptica NSC:N192805 Endpt:LC50 ExpId:AVGDATA hiConc:2.0	0.86	0.749	1.0

(S41) COMPARE between *S. elliptica* extracts, pristimerin (**2**) and 11*β*-hydroxypristimerin (**1**).

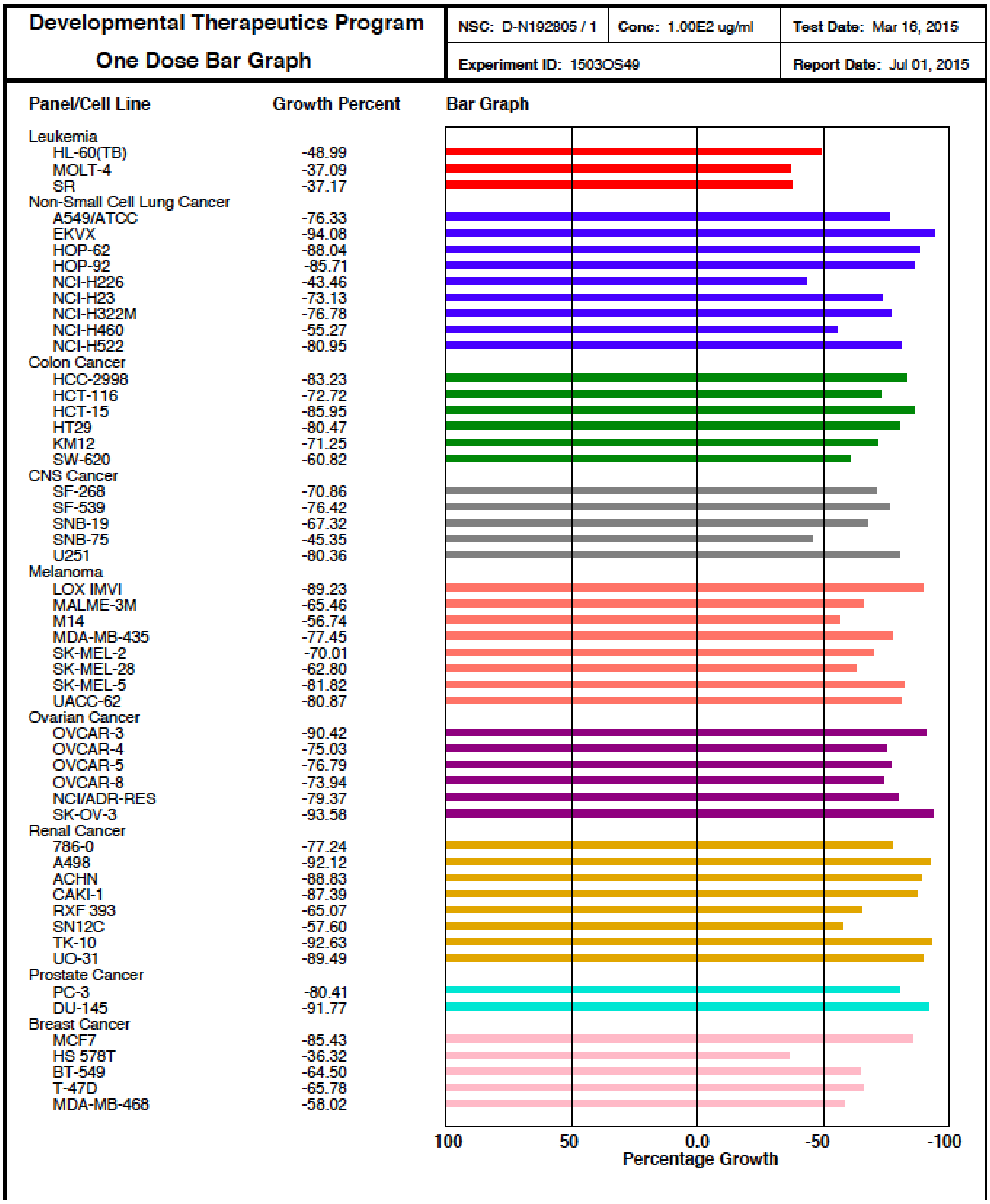

(S42) NCI-60 single dose bar graph of *S. elliptica* root wood extract.

Developmental Therapeutics Program One Dose Mean Graph	NSC: D-N192805 / 1	Conc: 1.00E2 ug/ml	Test Date: Mar 16, 2015
	Experiment ID: 1503OS49		Report Date: Jul 01, 2015

Panel/Cell Line	Growth Percent
**Leukemia**	
HL-60(TB)	-48.99
MOLT-4	-37.09
SR	-37.17
**Non-Small Cell Lung Cancer**	
A549/ATCC	-76.33
EKVX	-94.08
HOP-62	-88.04
HOP-92	-85.71
NCI-H226	-43.46
NCI-H23	-73.13
NCI-H322M	-76.78
NCI-H460	-55.27
NCI-H522	-80.95
**Colon Cancer**	
HCC-2998	-83.23
HCT-116	-72.72
HCT-15	-85.95
HT29	-80.47
KM12	-71.25
SW-620	-60.82
**CNS Cancer**	
SF-268	-70.86
SF-539	-76.42
SNB-19	-67.32
SNB-75	-45.35
U251	-80.36
**Melanoma**	
LOX IMVI	-89.23
MALME-3M	-65.46
M14	-56.74
MDA-MB-435	-77.45
SK-MEL-2	-70.01
SK-MEL-28	-62.80
SK-MEL-5	-81.82
UACC-62	-80.87
**Ovarian Cancer**	
OVCAR-3	-90.42
OVCAR-4	-75.03
OVCAR-5	-76.79
OVCAR-8	-73.94
NCI/ADR-RES	-79.37
SK-OV-3	-93.58
**Renal Cancer**	
786-0	-77.24
A498	-92.12
ACHN	-88.83
CAKI-1	-87.39
RXF 393	-65.07
SN12C	-57.60
TK-10	-92.63
UO-31	-89.49
**Prostate Cancer**	
PC-3	-80.41
DU-145	-91.77
**Breast Cancer**	
MCF7	-85.43
HS 578T	-36.32
BT-549	-64.50
T-47D	-65.78
MDA-MB-468	-58.02
Mean	-73.04
Delta	21.04
Range	57.76

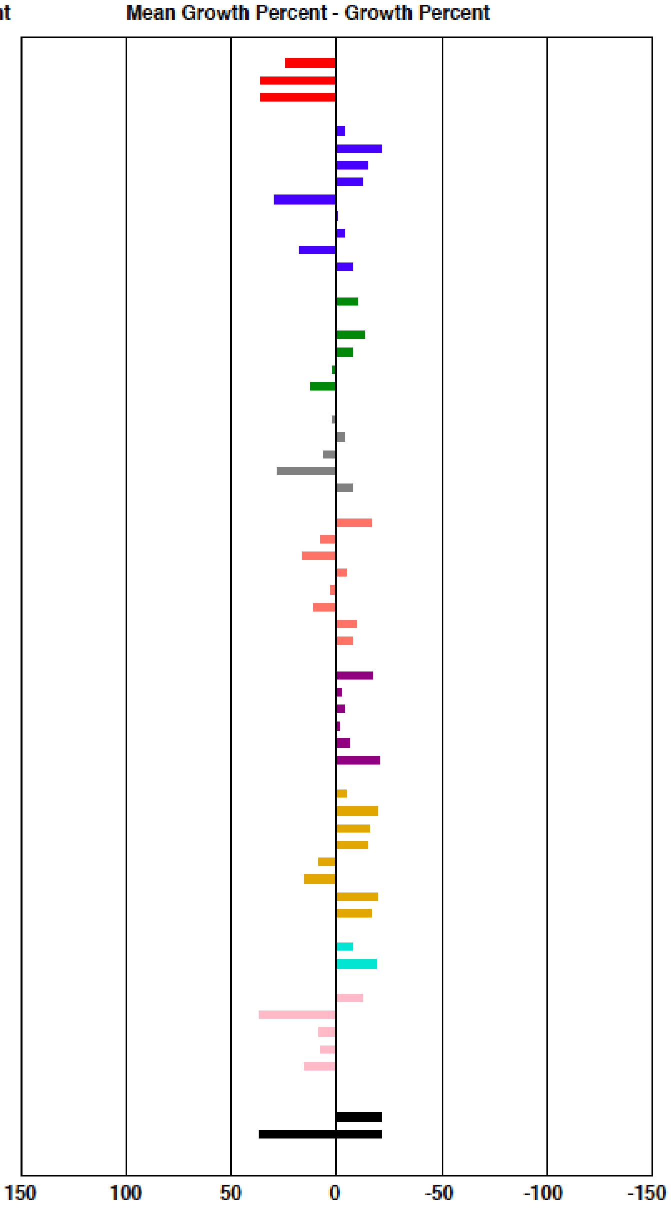

(S43) NCI-60 single dose mean bar graph of *S. elliptica* root wood extract.

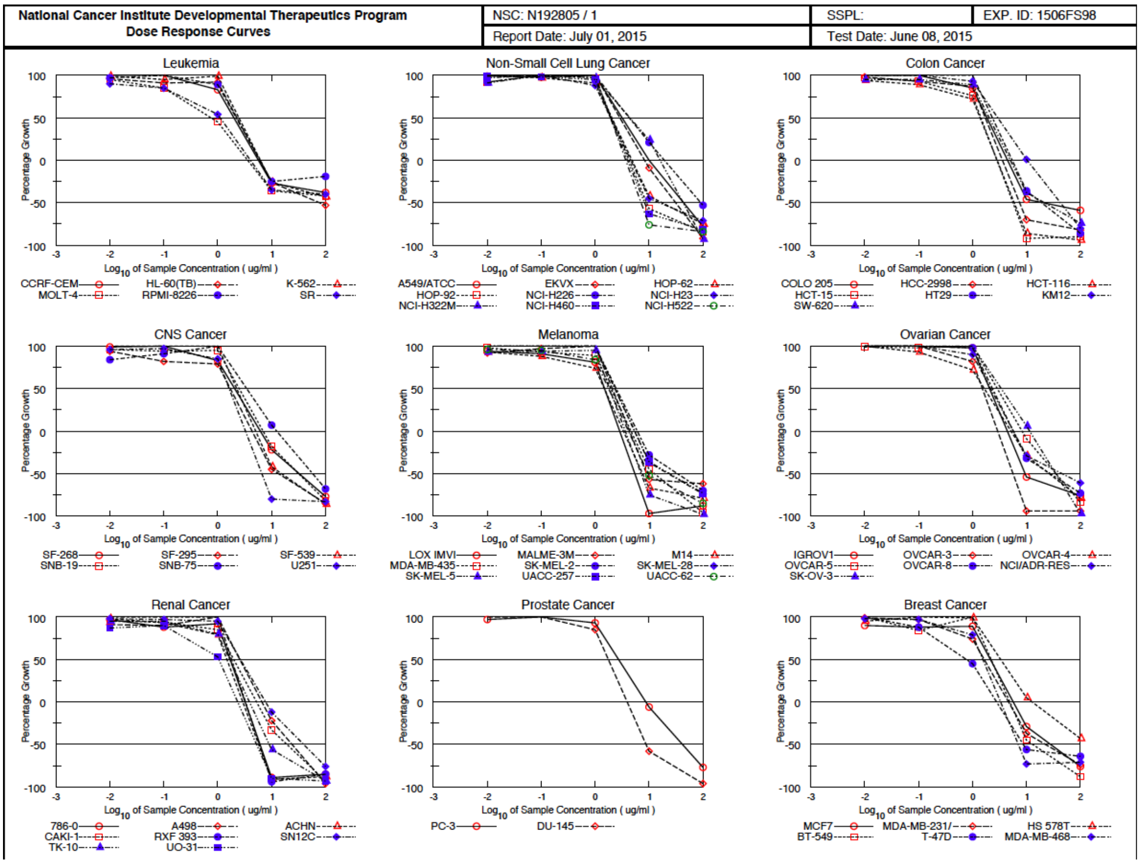

(S44) NCI-60 dose response curves for *S. elliptica* root wood extract.

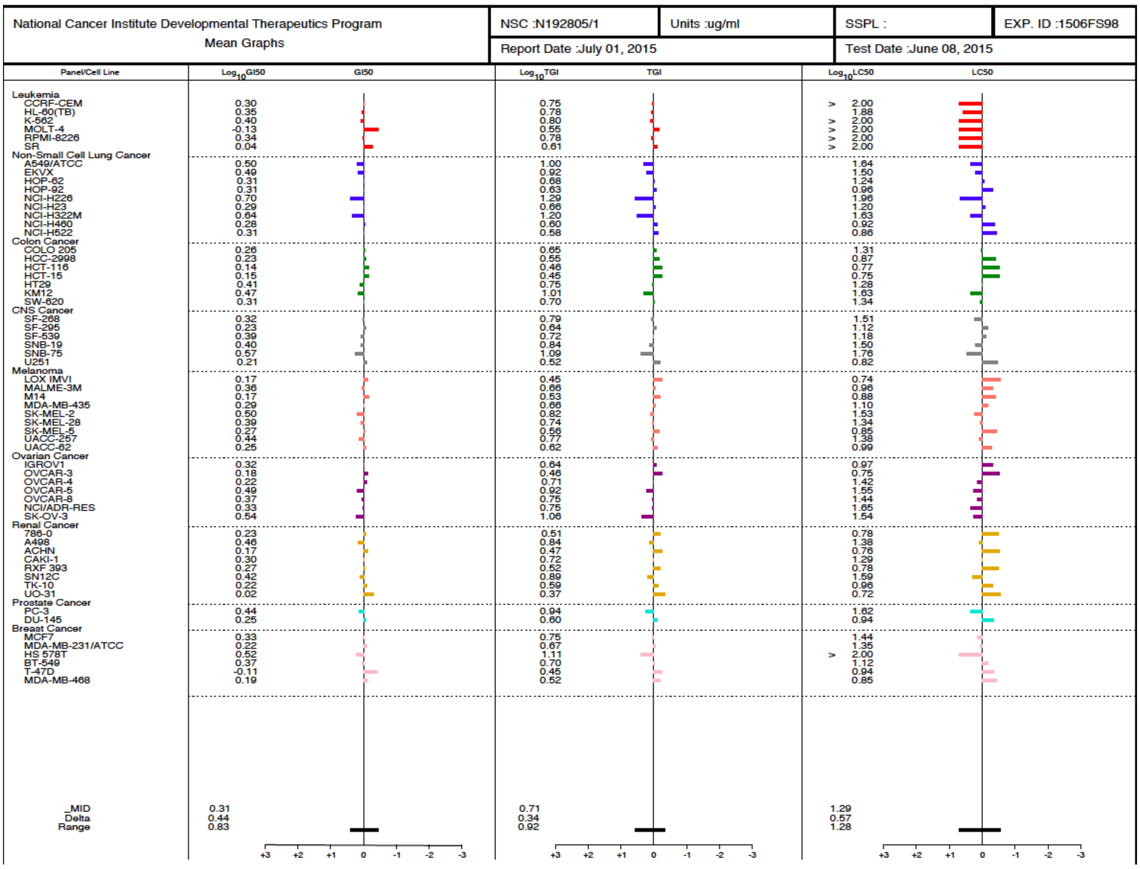

(S45) NCI-60 5-dose mean bar graph of *S. elliptica* root wood extract.

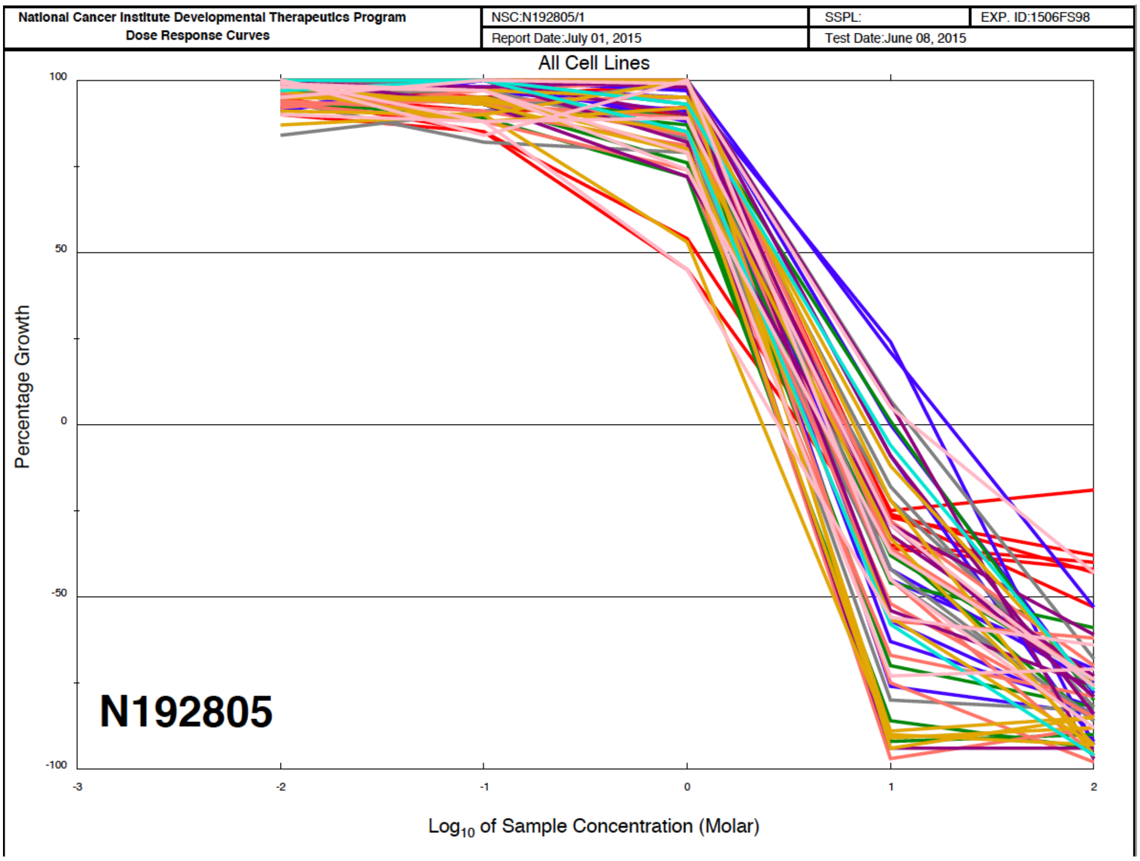

(S46) NCI-60 cumulative dose response curves for *S. elliptica* root wood extract.

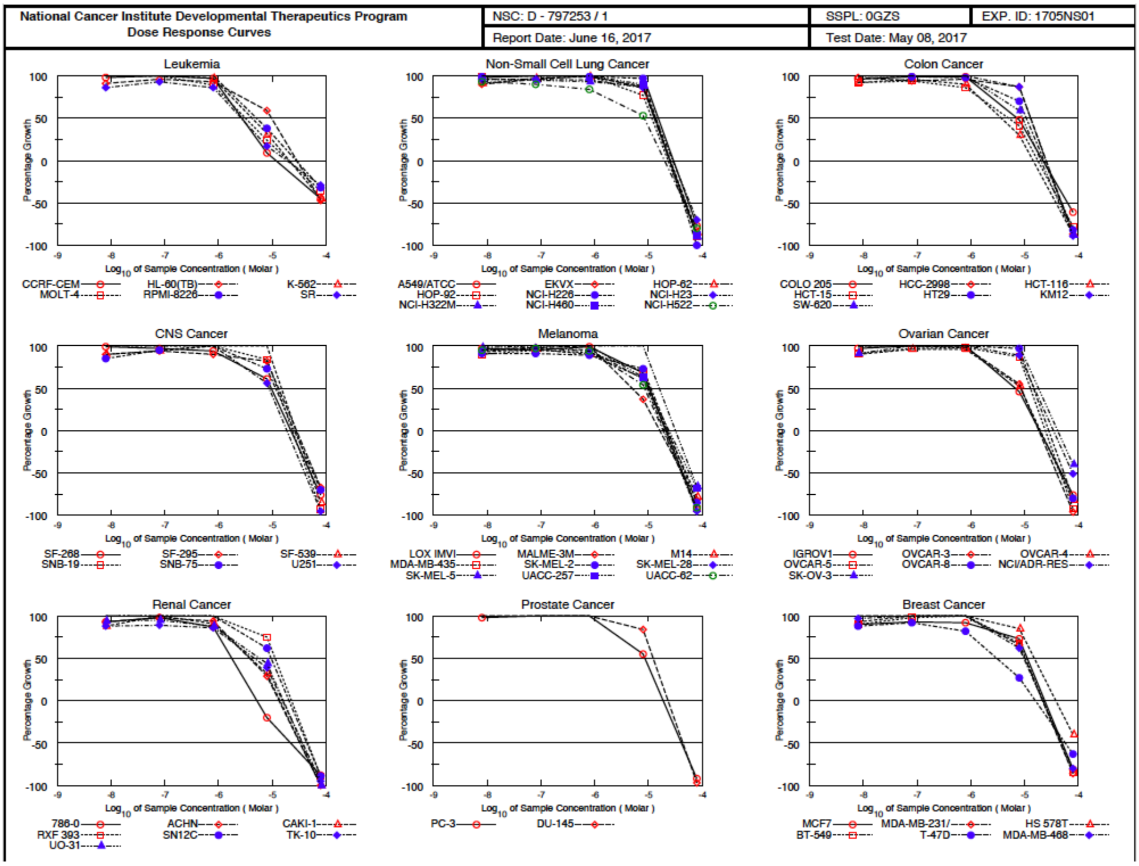

(S47) NCI-60 dose response curves for 11β-hydroxypristimerin (**1**).

National Cancer Institute Developmental Therapeutics Program
Mean Graphs

NSC : D - 797253/1 | Units :Molar | SSPL :0GZS | EXP. ID :1705NS01
Report Date :June 16, 2017 | Test Date :May 08, 2017

Panel/Cell Line	Log_{10}GI50	Log_{10}TGI	Log_{10}LC50
**Leukemia**			
CCRF-CEM	-5.56	-4.93	> -4.10
HL-60(TB)	-5.01	-4.54	> -4.10
K-562	-5.37	-4.68	> -4.10
MOLT-4	-5.50	-4.70	> -4.10
RPMI-8226	-5.29	-4.56	> -4.10
SR	-5.57	-4.72	> -4.10
**Non-Small Cell Lung Cancer**			
A549/ATCC	-4.80	-4.52	-4.24
EKVX	-4.89	-4.60	-4.31
HOP-62	-4.88	-4.59	-4.30
HOP-92	-4.94	-4.63	-4.33
NCI-H226	-4.86	-4.60	-4.35
NCI-H23	-4.87	-4.55	-4.23
NCI-H322M	-4.88	-4.60	-4.32
NCI-H460	-4.87	-4.59	-4.31
NCI-H522	-5.08	-4.70	-4.32
**Colon Cancer**			
COLO 205	-5.13	-4.65	-4.19
HCC-2998	-4.89	-4.60	-4.31
HCT-116	-5.44	-4.84	-4.42
HCT-15	-5.29	-4.75	-4.33
HT29	-4.97	-4.64	-4.31
KM12	-4.89	-4.60	-4.32
SW-620	-5.03	-4.69	-4.36
**CNS Cancer**			
SF-268	-5.02	-4.65	-4.29
SF-295	-4.88	-4.55	-4.21
SF-539	-4.80	-4.54	-4.28
SNB-19	-4.91	-4.62	-4.34
SNB-75	-4.93	-4.59	-4.24
U251	-5.06	-4.73	-4.40
**Melanoma**			
LOX IMVI	-4.98	-4.67	-4.36
MALME-3M	-5.31	-4.79	-4.37
M14	-5.01	-4.65	-4.30
MDA-MB-435	-4.98	-4.67	-4.36
SK-MEL-2	-4.95	-4.63	-4.32
SK-MEL-28	-5.03	-4.71	-4.39
SK-MEL-5	-4.78	-4.49	-4.19
UACC-257	-5.00	-4.61	-4.23
UACC-62	-5.07	-4.72	-4.38
**Ovarian Cancer**			
IGROV1	-5.17	-4.72	-4.31
OVCAR-3	-5.06	-4.73	-4.40
OVCAR-4	-5.08	-4.71	-4.33
OVCAR-5	-4.89	-4.61	-4.33
OVCAR-8	-4.83	-4.55	-4.26
NCI/ADR-RES	-4.82	-4.46	-4.10
SK-OV-3	-4.72	-4.37	> -4.10
**Renal Cancer**			
786-0	-5.75	-5.28	-4.66
ACHN	-5.42	-4.87	-4.48
CAKI-1	-5.40	-4.86	-4.48
RXF 393	-4.95	-4.64	-4.34
SN12C	-5.02	-4.68	-4.35
TK-10	-5.32	-4.80	-4.43
UO-31	-5.24	-4.79	-4.44
**Prostate Cancer**			
PC-3	-5.06	-4.72	-4.38
DU-145	-4.91	-4.63	-4.36
**Breast Cancer**			
MCF7	-4.95	-4.62	-4.30
MDA-MB-231/ATCC	-4.99	-4.66	-4.33
HS 578T	-4.82	-4.42	> -4.10
BT-549	-4.98	-4.65	-4.32
T-47D	-5.52	-4.80	-4.24
MDA-MB-468	-5.01	-4.66	-4.31
MID	-5.06	-4.66	-4.3
Delta	0.69	0.62	0.36

(S48) NCI-60 5-dose mean bar graph of 11*β*-hydroxypristimerin (**1**).

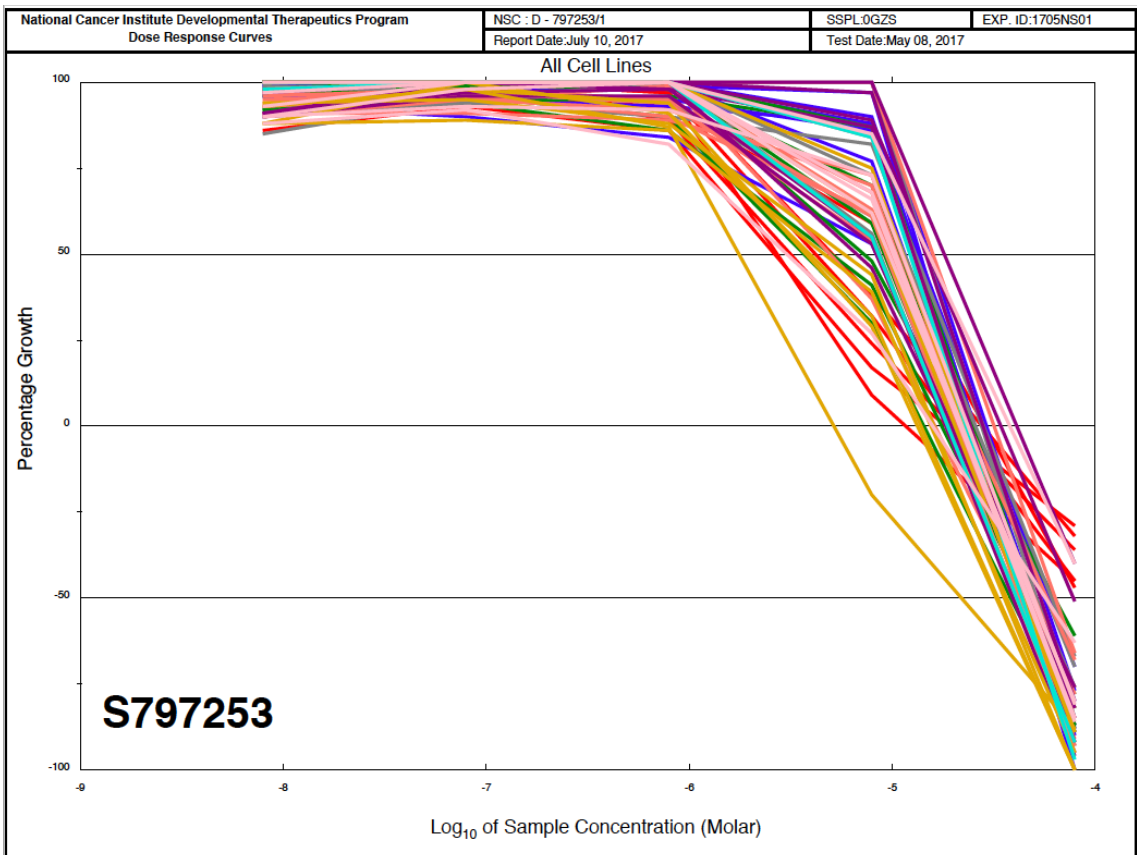

(S49) NCI-60 cumulative dose response curves for 11*β*-hydroxypristimerin (**1**).

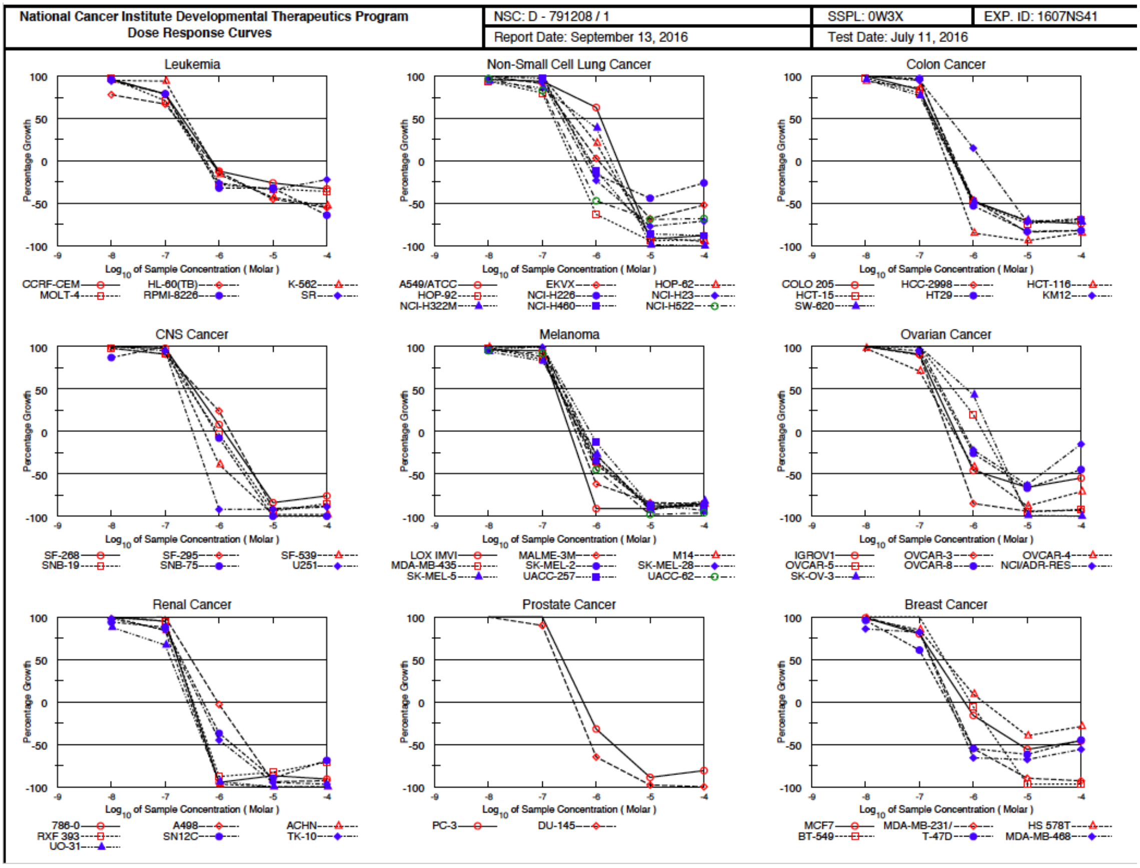

(S50) NCI-60 dose response curves for pristimerin (**2**).

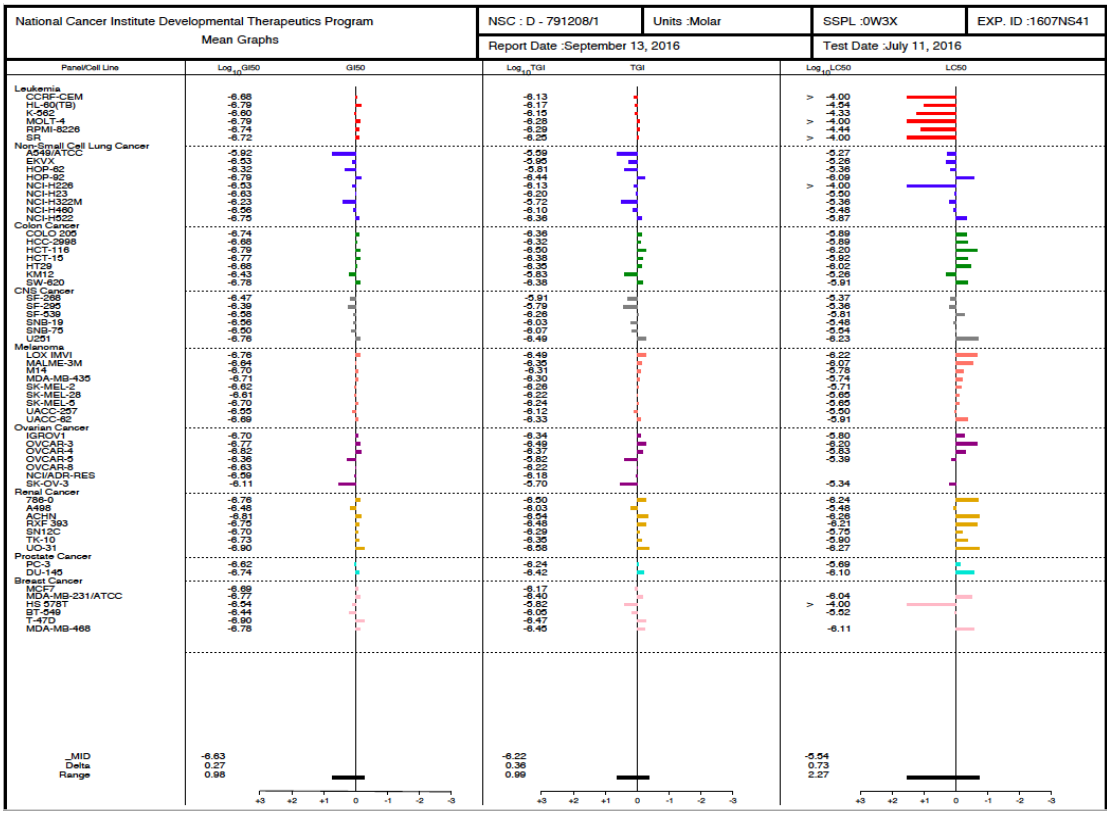

(S51) NCI-60 5-dose mean bar graph of pristimerin (**2**).

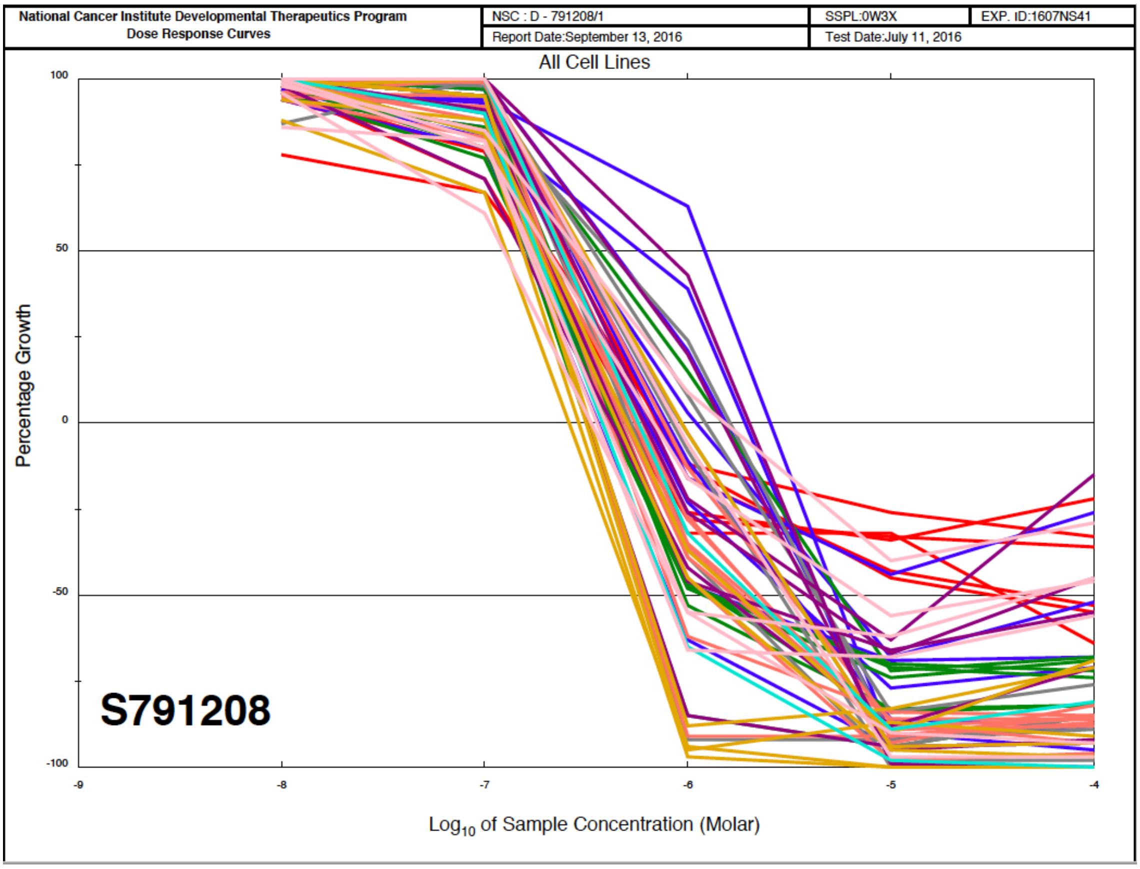

(S52) NCI-60 cumulative dose response curves for pristimerin (**2**).

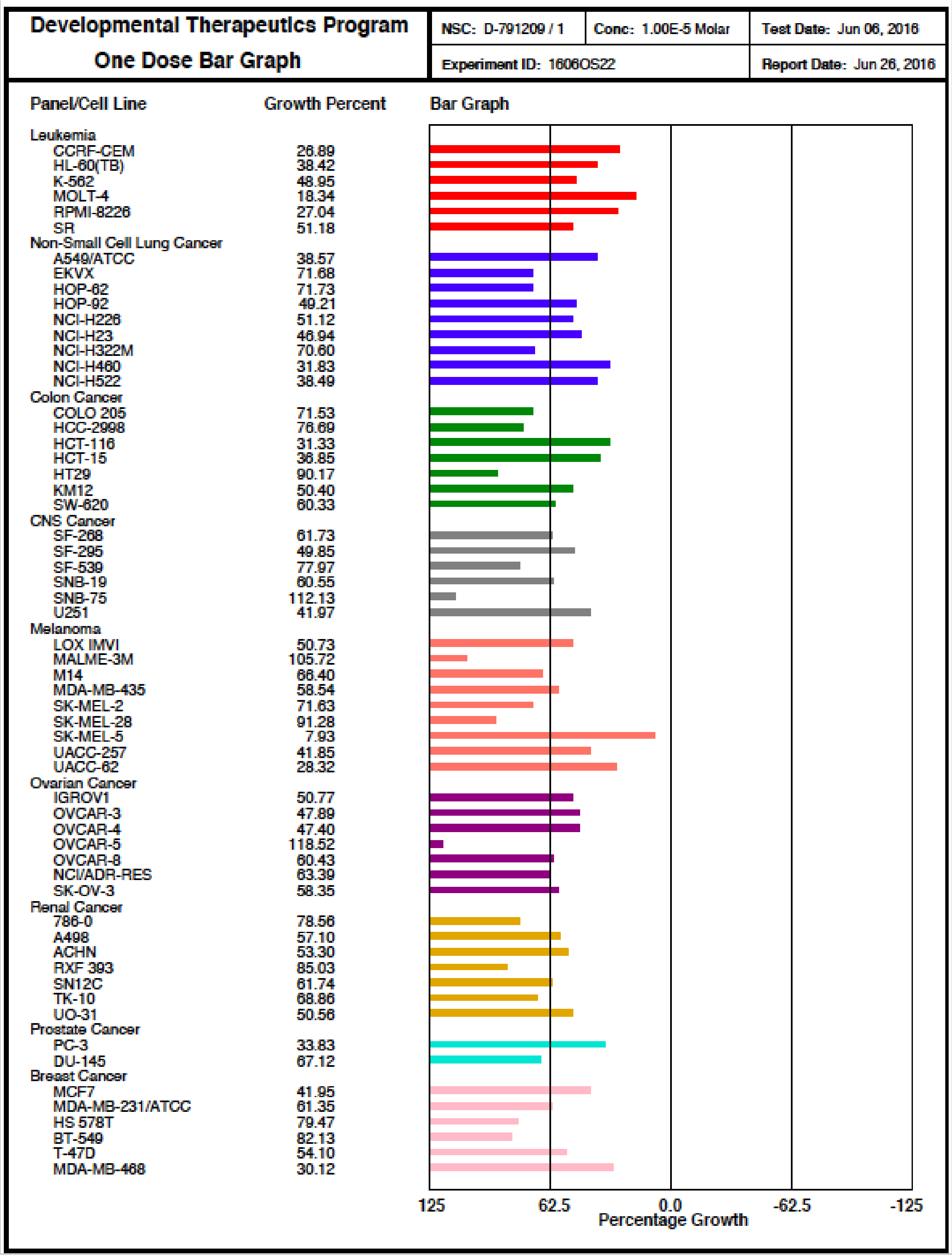

(S53) NCI-60 single dose bar graph of 6-oxopristimerol (**3**).

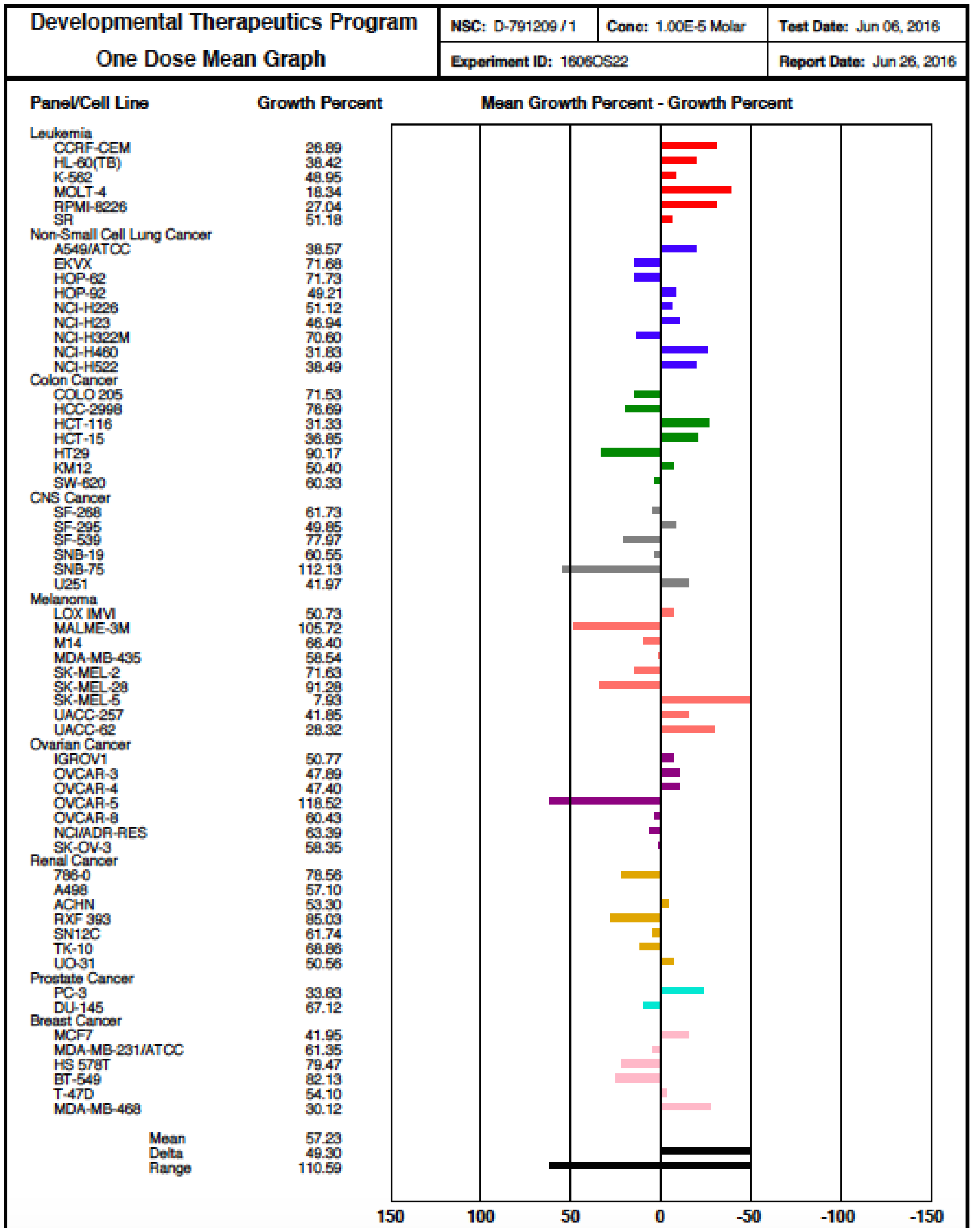

(S54) NCI-60 singe dose mean bar graphs of 6-oxopristimerol (**3**).
